# A General Model for the Seasonal to Decadal Dynamics of Leaf Area

**DOI:** 10.1111/gcb.70125

**Published:** 2025-03-21

**Authors:** Boya Zhou, Wenjia Cai, Ziqi Zhu, Han Wang, Sandy P. Harrison, I. Colin Prentice

**Affiliations:** ^1^ Georgina Mace Centre for the Living Planet, Department of Life Sciences Imperial College London Ascot UK; ^2^ Department of Earth System Science, Ministry of Education Key Laboratory for Earth System Modelling, Institute for Global Change Studies Tsinghua University Beijing China; ^3^ School of Archaeology, Geography and Environmental Science (SAGES) University of Reading Reading UK

**Keywords:** eco‐evolutionary optimality theory, gross primary production, land‐atmosphere exchanges, land‐surface modeling, leaf area index, seasonal leaf phenology

## Abstract

Leaf phenology, represented at the ecosystem scale by the seasonal dynamics of leaf area index (LAI), is a key control on the exchanges of CO_2_, energy, and water between the land and atmosphere. Robust simulation of leaf phenology is thus important for both dynamic global vegetation models (DGVMs) and land‐surface representations in climate and Earth System models. There is no general agreement on how leaf phenology should be modeled. However, a recent theoretical advance posits a universal relationship between the time course of “steady‐state” gross primary production (GPP) and LAI—that is, the mutually consistent LAI and GPP that would pertain if weather conditions were held constant. This theory embodies the concept that leaves should be displayed when their presence is most beneficial to plants, combined with the reciprocal relationship of LAI and GPP via (a) the Beer's law dependence of GPP on LAI, and (b) the requirement for GPP to support the allocation of carbon to leaves. Here we develop a global prognostic LAI model, combining this theoretical approach with a parameter‐sparse terrestrial GPP model (the P model) that achieves a good fit to GPP derived from flux towers in all biomes and a scheme based on the P model that predicts seasonal maximum LAI as the lesser of an energy‐limited rate (maximizing GPP) and a water‐limited rate (maximizing the use of available precipitation). The exponential moving average method is used to represent the time lag between leaf allocation and modeled steady‐state LAI. The model captures satellite‐derived LAI dynamics across biomes at both site and global levels. Since this model outperforms the 15 DGVMs used in the TRENDY project, it could provide a basis for improved representation of leaf‐area dynamics in vegetation and climate models.

## Graphical abstract

We extend the theoretical basis for understanding how climate affects ecosystem processes and provide an optimality‐based model to predict the seasonal dynamics of leaf phenology in response to climate change that could improve land‐surface models.

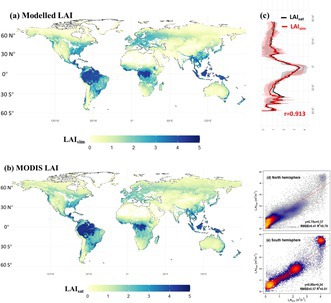

## 1 Introduction

Leaf phenology refers to the annual life cycle of leaves, including budburst and unfolding, growth, senescence, and abscission. At the ecosystem level, these processes give rise to the seasonal cycle in leaf area (Xie et al. 2018). The seasonal cycle of leaf area, as measured by the leaf area index (LAI), imposes constraints on the geographic distribution of tree species (Chuine 2010) and has a strong influence on the global carbon and water cycles (Piao et al. 2019). Thus, leaf phenology is important for understanding and predicting the interactions between terrestrial ecosystems and climate (Zhao et al. 2013) and predicting the seasonal time course of LAI is an essential element of land surface models.

The global controls of LAI have gained increasing attention in recent years (Tang et al. 2016; Piao et al. 2019) but there is still no accepted way to represent the seasonal changes in LAI in global models. Most land surface models predict LAI as outcomes of carbon allocation. For example, the Community Land Model Version 5 (CLM5.0) first estimates the carbon flux to leaves, taking account of the effects of climate‐related variables, including soil temperature and moisture, then converts leaf carbon mass to the equivalent LAI, assuming a fixed Specific Leaf Area (SLA) for each vegetation type (Lawrence et al. 2011). The Community Atmosphere Biosphere Land Exchange (CABLE) model (Xia et al. 2017) and the Canadian Terrestrial Ecosystem Model (CTEM) (Arora and Boer 2005) divide vegetation growth into phenophases and prescribe different proportions of photosynthate to be allocated to leaves in each phase. Leaf area is then derived based on empirical relationships with biophysical variables, including SLA. In most land surface models, biophysical variables are set to fixed values for each vegetation type (Kato et al. 2013; Meiyappan et al. 2015; Vuichard et al. 2019; Walker et al. 2017; Lienert and Joos 2018). This approach requires many empirical values to be specified, introducing considerable uncertainty for global‐scale simulations (Xia et al. 2017). Some other models, such as the Integrated BIosphere Simulator (IBIS) model (Yuan et al. 2014) and the Interactions between Soil, Biosphere, and Atmosphere (ISBA) model (Gibelin et al. 2006; Delire et al. 2020), model SLA more dynamically, allowing it to be modified by leaf nitrogen concentration and atmospheric carbon dioxide. However, all of these approaches are ad hoc, lacking an explicit theoretical basis; and indeed, theory for the seasonal dynamics of carbon allocation is still incomplete (Franklin et al. 2012; Hartmann et al. 2020) and thus unable to provide a well‐founded basis for modeling leaf phenology.

An extensive literature deals with the timing of phenological transitions, most commonly the start of season (SOS) and end of season (EOS), and many models have been published describing the controls of these transitions. Current models for spring phenology in cold‐winter climates are of two main types: one‐phase models only consider the need for plants to accumulate heat during the ecodormancy phase, when bud regrowth is hampered by unfavorable external conditions (Reaumur 1735; McMaster and Wilhelm 1997; Zhou and Wang 2018); two‐phase models also consider the amount of chilling during the (preceding) endodormancy phase, which is an internally regulated process, whereby growth is suppressed even in favorable environmental conditions (Cannell and Smith 1983; Chuine et al. 2000; Caffarra et al. 2011; Fu et al. 2020). Some other models include the influence of environmental factors on more complex aspects of phenology, such as the DormPhot model (Caffarra et al. 2011) which considers the effects of chilling, forcing, and photoperiod on dormancy induction and phase of forcing simultaneously. The lack of consensus about how to model these key phenological transitions, coupled with the fact that these phenological models are largely species‐ or plant type‐specific, means that they are not well suited to application in a global modeling context. This is the major reason for focusing on modeling seasonal changes in LAI in order to improve the representation of vegetation phenology in LSMs.

Seasonal changes in incoming solar radiation are an important driver of the seasonal cycle of LAI in extratropical regions. However, experiments have shown that air temperature is the main driver of interannual phenological variability in northern temperate and high‐latitude regions (Fu et al. 2019; Meng et al. 2021), while precipitation is the dominant factor influencing phenological changes in drylands (Currier and Sala 2022) with a major impact on spring phenology (Castillioni et al. 2022). The cyclical patterns of solar radiation and vapor pressure deficit are the two most important environmental variables linked to the production of new leaves and the shedding of old leaves in tropical evergreen forests (Chen et al. 2019). For robust global‐scale modeling, it would be useful to seek an approach that could account for such dependencies via a single mechanism applicable to all biomes. In this spirit, Jolly et al. (2005) proposed the Growing Season Index Model, which considers photoperiod, vapor pressure deficit, and air temperature as predictors. But even though this model—and others derived from it (Schaphoff et al. 2018)—have had some success in simulating seasonal leaf area dynamics, they are still limited in climate‐change applications because of their lack of theoretical underpinnings.

Xin et al. (2018) proposed a promising alternative method for simulating the seasonal dynamics of LAI, in which LAI time series are estimated from the seasonal time course of GPP via the Beer's law dependence of GPP on LAI (Swinehart 1962) and the reciprocal requirement for GPP to support LAI development. The underlying principle—that seasonal variations in LAI are coordinated with variations in GPP—can be considered an eco‐evolutionary optimality hypothesis (Harrison et al. 2021), because it implies that leaves are displayed at (or near) the time when they are able to be most productive. A “semi‐prognostic” LAI dynamics model (Xin et al. 2020) based on this approach was developed. It requires annual peak LAI data from satellite data as input to provide an upper bound for predicted LAI. This model has been shown to capture both the spatial pattern and seasonal variation of satellite‐derived LAI on a global scale, albeit with some limitations in high‐latitude and tropical areas. Zhou et al. (2023) further developed the model of Xin et al. (2020) by adding a scheme to predict seasonal maximum LAI, thus allowing a fully prognostic (i.e., independent of satellite data) simulation of the seasonal time course of LAI. This model can generally capture the seasonal variation of LAI, although it performs less well for biomes with typically high LAI such as evergreen broadleaf forests and closed shrublands, and still requires nine parameters to be estimated separately for each biome.

Here we adopt the theoretical approach proposed by Xin et al. (2018, 2020) but in addition, we build on the development of a universal, first principles‐based model for GPP (the P model: Prentice et al. 2014; Wang, Prentice, et al. 2017; Stocker et al. 2020; Mengoli et al. 2022). The P model rests on eco‐evolutionary optimality hypotheses that have been tested one‐by‐one using independent data. When forced by satellite‐derived LAI data, it achieves good representations of the seasonal cycle of GPP across all biomes (Stocker et al. 2020) and a realistic simulation of site‐based GPP trends (Cai and Prentice 2020), without requiring biome‐specific parameters. We also apply a top‐down algorithm for predicting seasonal maximum LAI (Zhu et al. 2023; Cai et al. 2025), which makes use of the P model. This algorithm is based on the principle that this quantity is limited either by available energy (in which case a profit‐maximizing criterion applies) or by water availability (when it is limited by the transpiration demands of photosynthesis). This allows us to build a fully prognostic solution for simulating LAI time series, which combines the robustness of the P model with our top‐down criteria for seasonal maximum LAI and the conceptual simplicity and generality of the approach pioneered by Xin et al. (2018, 2020).

The present study has two objectives: (1) to develop a global LAI model, independent of satellite data, that can simulate seasonal to multidecadal dynamics of LAI both at eddy‐covariance flux sites and globally; and (2) to evaluate the model across biomes, using both in situ and satellite measurements.

## 2 Data and Methods

### 2.1 Model Overview

**FIGURE 1:**
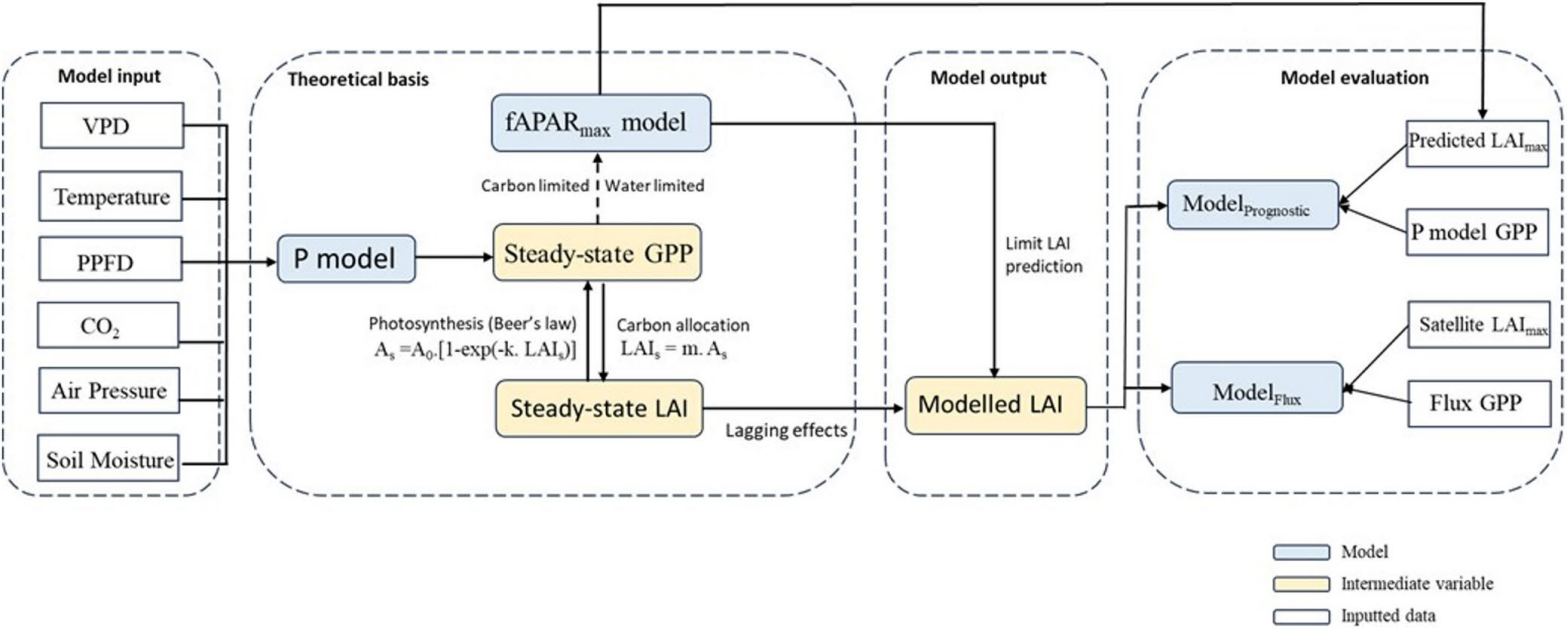
Workflow of the LAI model and its evaluation. Notation: *A*_s_, steady‐state GPP (g C m^−2^ day^−1^); *A*_0_, potential GPP (g C m^−2^ day^−1^); LAI_s_, steady‐state LAI (m^−2^ m^−2^); LAI_max_, annual peak LAI (m^2^ m^−2^). *A*_s_ and LAI_s_ are the mutually consistent GPP and LAI that would be supported if environmental conditions were held constant. *A*_0_ is the GPP that would be supported under the same conditions if all light incident on the canopy were absorbed by leaves, i.e., as LAI→∞. *k* is the light extinction coefficient (≈0.5) and *m* is a quantity (to be determined) relating *A*_s_ and LAI_s_.

The modeling workflow has two steps (Figure 1): (1) Derivation of steady‐state LAI dynamics from the time course of potential GPP through the Beer's law dependence of GPP on LAI and the requirement for GPP to support LAI development. (2) Simulation of actual LAI dynamics, allowing for the time lag of leaf allocation behind steady‐state LAI.

#### 2.1.1 Prediction of Gross Primary Production

The P model is a universal and extensively validated light use efficiency (LUE) model for predicting gross primary production (GPP) (Wang, Lu, et al. 2017; Wang, Prentice, et al. 2017; Stocker et al. 2020). It integrates the Farquhar–von Caemmerer–Berry (FvCB) model (Farquhar et al. 1980) with ecological evolutionary optimality (EEO) principles to represent the adaptation of stomatal behavior and photosynthetic capacities to environmental conditions (Prentice et al. 2014; Wang, Lu, et al. 2017; Wang, Prentice, et al. 2017). A study with a more detailed description of this model has demonstrated its ability to effectively capture observed trends in GPP across multiple eddy‐covariance flux sites (Stocker et al. 2020). Moreover, its performance in explaining GPP trends is comparable to that of more complex models (Mengoli et al. 2022).

Based on the P model, GPP (*A*) can be expressed as a product of fAPAR, Light Use Efficiency (LUE) and PPFD. Potential gross primary production (*A*_0_) is the GPP that would be achieved if fAPAR = 1 and can be calculated from Equation (1).

(1) A=fAPAR×LUE×PPFD

In Equation (1), LUE is expressed as:

(2) $$\;\mathrm{LUE}=\varphi_{0}\;m\;\sqrt{1-{\left(\frac{c*}{m}\right)}^{\frac{2}{3}}}$$

(3) $$\;m=\frac{c_{a}-\Gamma^{*}}{\left\{c_{a}+2\Gamma^{*}+3\Gamma^{*}\sqrt{\left[1.6\eta^{*}D\beta^{-1}{\left(K+\Gamma^{*}\right)}^{-1}\right]}\;\right\}}$$

Here, *φ*_0_ is the intrinsic quantum yield (mol CO_2_ mol^−1^ photon), *m* represents the impact of leaf‐internal CO_2_ on carbon assimilation. *c*_a_ is the ambient CO_2_ partial pressure (Pa), Γ* is the CO_2_ partial pressure compensation point (Pa), *K* is the effective Michaelis–Menten coefficient of Rubisco (Pa), η* is the viscosity of water relative to its value at 25°C (dimensionless), *β* is the cost factor for carboxylation and transpiration at 25°C, equaling 146 (dimensionless). c* is a dimensionless constant, equaling 41.

#### 2.1.2 Prediction of Seasonal Maximum LAI

We treated fAPAR (the fraction of incident photosynthetically active radiation that is absorbed by leaves) and LAI as interchangeable, assuming they are monotonically related by Beer's law (Swinehart 1962; Figure 1). Global spatial patterns and temporal trends of the seasonal maximum value of fAPAR (fAPAR_max_) or LAI (LAI_max_) can be predicted to first order based on eco‐evolutionary optimality principles (Zhu et al. 2023; Cai et al. 2025).

It is hypothesized that over annual and longer time scales, carbon allocation to foliage is restricted by either water supply or photosynthesis. A transpiring canopy cannot be sustained if insufficient root‐zone water causes prolonged stomatal closure, while building and maintaining leaves (and supplying them with water and nutrients) incurs a carbon cost that cannot exceed the carbon they fix indefinitely. These situations are called ‘water limited’ and ‘energy limited’ respectively (Cai et al. 2025).

Under water limitation, it is assumed that plants adjust their rooting behavior to extract a portion of annual precipitation from the soil, irrespective of its distribution throughout the year, and allocate carbon to leaves so that all this water is transpired, thereby maximizing gross primary production (GPP).

General expressions for GPP (Equation 4) and transpiration (Equation 5) are:

(4) $$A=G_{s}c_{a}\left(1-\chi \right)=\text{fAPAR}.A_{0}$$

and

(5) $$E=1.6\;G_{s}\;D$$

where *A* is GPP (leaf respiration was ignored for simplicity), *G*_s_ is the canopy conductance for CO_2_, *c*_a_ is the ambient CO_2_ partial pressure, and *χ* is the ratio of leaf‐internal CO_2_ partial pressure to *c*_a_. *A*_0_ is the potential GPP, i.e. the GPP that would be achieved if fAPAR = 1 and can be calculated from Equation (1). *E* is transpiration, and *D* is the vapor pressure deficit (VPD).

Transpiration is assumed to use a prescribed fraction (*f*_0_) of annual total precipitation (*E* = *f*_0_ *P*). In the original fAPAR_max_ framework (Zhu et al. 2023; Cai et al. 2025) *f*_0_ was assigned a single constant value, equaling 0.62. Here, we have substituted this constant value with an empirical equation that relates *f*_0_ to the climatological Aridity Index (AI) (Good et al. 2017) (Equation 6), thus allowing *f*_0_ to decline as AI increases beyond a value close to the separation between energy‐ and water‐limited regimes. The climatic AI is the ratio of mean annual potential evapotranspiration to mean annual precipitation (Good et al. 2017) calculated using climate data for a 20‐year period (2001–2019). The precipitation data were obtained directly from the CRU data set, and PET was calculated using SPLASH v1.0 with temperature, precipitation, and cloud cover data as inputs (Davis et al. 2017).

(6) $$f_{0}=0.65\times e^{-b\times \ln^{2}\left(\frac{\mathrm{AI}}{1.9}\right)}$$

where 0.65 is the maximum value, 1.9 is the AI at which this maximum occurs, and *b* = 0.604169.

Equation (6), together with Equations (4) and (5), yields Equation (11) as the expression of water‐limited fAPAR.

Under energy limitation, it is assumed that plants allocate carbon to leaves to maximize GPP after accounting for the costs of constructing and maintaining leaves and supplying them with water and nutrients. This approach leads to a clear optimum because investing in leaf tissue yields diminishing returns due to mutual leaf shading. We now define the net carbon profit (*P*_n_) as:

(7) $$P_{n}=A_{0}\times \text{fAPAR}-z\times \mathrm{LAI}$$

and assume Beer's Law:

(8) $$\begin{array}{c} \text{fAPAR}=1-\exp\left(-k\times \mathrm{LAI}\right)\; \end{array}$$

Then

(9) $$P_{n}=A_{0}\times \text{fAPAR}+\left(\frac{z}{k}\right)\ln\left(1-\text{fAPAR}\right)$$

and

(10) $$\frac{\partial P_{n}}{\partial \left(\text{fAPAR}\right)}=A_{0}-\frac{\frac{z}{k}}{1-\text{fAPAR}}$$

Setting Equation (10) to zero yields Equation (11) as the expression of energy‐limited fAPAR; the turning point is a maximum in *P*_n_. The globally fitted value for *z* was 12.227 mol^−2^ m^−2^ a^–1^, obtained by non‐linear least squares regression using the log‐sum‐exp function to approximate the minimum function.

The seasonal maximum fAPAR can then be represented by Equation (11) and the seasonal maximum LAI by Equation (12) (Cai et al. 2025):

(11) $${\text{fAPAR}}_{\max}=\min\;\left\{1–z/\left(kA_{0}\right),\left[c_{a}\;\left(1–\chi \right)/1.6\;D\right]\;\left[f_{0}\;P/A_{0}\right]\right\}$$

(12) $${\mathrm{LAI}}_{\max}=–\left(1/k\right)\;\ln\;\left(1–{\text{fAPAR}}_{\max}\right)$$

In Equation (11), the first part represents the energy‐limited equation, and the second part represents the water‐limited equation. *k* is the light extinction coefficient (set at 0.5), *D* is the mean vapor pressure deficit (VPD) during the growing season (> 0°C), *A*_0_ is the annual total potential GPP (mol m^−2^ year^−1^), and *P* is the annual total precipitation (mol m^−2^ year^−1^).

#### 2.1.3 Simulating the Dynamics of LAI

GPP is dependent on LAI (with the relationship governed by meteorological conditions) since leaves are the main organs of photosynthesis. On the other hand, GPP represents the total rate of carbon fixation, of which some fraction accrues to leaves. A general principle governing leaf phenology is that it should allow plants to achieve competitive success through maximizing photosynthesis subject to environmental constraints (Franklin et al. 2012). Based on the mutual relationship between GPP and LAI, Xin et al. (2018, 2020) proposed a criterion consistent with this principle, based on the concepts of steady‐state LAI and GPP—that is, the LAI and GPP that would be in equilibrium if weather conditions were constant (Equation 13). Given daily meteorological conditions, the steady‐state LAI is modeled on a daily basis by solving the closed system of equations (Equations 13 and 14) as follows:

(13) $$L_{s}=\min\left[\;m\;A_{s},{\mathrm{LAI}}_{\max}\right]$$

(14) $$A_{s}=A_{0}\;\left[1–\exp\;\left(–k\;L_{s}\right)\right]$$

where *L*_s_ denotes steady‐state LAI; *m* denotes the fraction of GPP allocated to LAI; and *A*_s_ denotes the steady‐state GPP. The solution is given by Equation (15):

(15) $$L_{s}=\min\left\{\;\mu +\left(\frac{1}{k}\right)W_{0}\;\left[–\mathrm{k\mu}\;\exp\;\left(–\mathrm{k\mu}\right)\right],{\mathrm{LAI}}_{\max}\right\}$$

where *μ* = *mA*_0_ and *W*_0_ is the principal branch of the Lambert W function. Further information on the solution of Equations 13 and 14 is provided in Note S1.

The processes governing canopy photosynthesis and vegetation phenology do not respond instantaneously to weather fluctuations, so there are inherent delays between the steady‐state LAI and the real‐time dynamic LAI. Although photosynthesis responds within minutes, the allocation of photosynthate to leaves and other tissues can take from days to several months (Mengoli et al. 2022; Sierra et al. 2022). Thus, it is reasonable to model GPP on an hourly to daily basis, and to simulate leaf‐area dynamics as a slower process, lagging steady‐state GPP. Without such a lag, LAI would fluctuate unrealistically from day to day. We adopt the exponential weighted moving average method (Equation 16) to incorporate this lag effect (Mengoli et al. 2022). This method is widely used in models to smooth out short‐term fluctuations and highlight longer‐term trends or cycles. It assigns exponentially decreasing weights to older LAI values, making it more responsive to recent changes in the data compared to a simple moving average (Yu et al. 2020):

(16) $${\mathrm{LAI}}_{\mathrm{sim}}=\alpha \times L_{s}\left[t\right]+\left(1-\alpha \right)\times L_{\mathrm{sim}}\left[t-1\right]$$

where the simulated actual LAI (LAI_sim_) is a weighted average of the steady‐state LAI value corresponding to the steady‐state GPP of the day (*L*_s_) and its acclimated value from the previous day, with weightings of *α* and (1−*α*) respectively. *α* is set to 0.067 here, corresponding to approximately 15 days of memory. We also tested a range of alternative values of α (0.33, 0.143, 0.1, 0.067, 0.05, 0.04, 0.033, 0.022 and 0.0167, corresponding to 3, 7, 10, 15, 20, 25, 30, 45 and 60 days) (Table S2; Figure S1). Further information on the derivation of Equation (16) and the parameter *α* is provided in Note S2.

#### 2.1.4 Estimation of *m*

Because actual LAI and GPP lag behind *L*_s_ and *A*_s_, the parameter *m* cannot be estimated directly from daily or monthly data (Xin 2016; Xin et al. 2019). However, a natural approximation for *m* is the ratio of annual average LAI to annual mean GPP:

(17) $$m=\left(\text{Annual mean}\;\mathrm{LAI}\right)/\left(\text{Annual mean}\;\mathrm{GPP}\right)$$

Xia et al. (2015) found that annual total GPP can be robustly estimated as a constant fraction (around 2/3) of the product of the length of the CO_2_ uptake period (growing season length, GSL) and the seasonal maximum of GPP (GPP_max_). Applying the same approach to LAI, we estimate *m* as follows:

(18) $$m=\left(\sigma_{1}\times {\mathrm{LAI}}_{\max}\times \mathrm{GSL}\right)/\left(\sigma_{2}\times {\mathrm{GPP}}_{\max}\times \mathrm{GSL}\right)$$

where LAI_max_ is the seasonal maximum LAI and *σ*_1_ and *σ*_2_ are parameters. We further assume that GPP_max_ is achieved when both *A*_0_ and fAPAR achieve their peak values, that is GPP_max_ = *A*_0max_ × fAPAR_max_, and that *A*_0max_ also follows the Xia et al. (2015) theory and can be represented as *A*_0max_ = *A*_0sum_/(*σ*_3_ × GSL), where *A*_0sum_ is the annual sum of *A*_0_. Thus:

(19) $$m=\left(\sigma_{1}\times {\mathrm{LAI}}_{\max}\times \mathrm{GSL}\times \sigma_{3}\times \mathrm{GSL}\right)/\left(\sigma_{2}\times A_{0\mathrm{sum}}\times {\text{fAPAR}}_{\max}\times \mathrm{GSL}\right)$$

Combining parameters (defining *σ* = *σ*_1_ × *σ*_3_/*σ*_2_), we obtain:

(20) $$m=\left(\sigma \times \mathrm{GSL}\times {\mathrm{LAI}}_{\max}\right)/\left(A_{0\mathrm{sum}}\times {\text{fAPAR}}_{\max}\right)$$

We estimate GSL as the length of the continuous above 0°C period longer than 5 days. LAI_max_ is the seasonal maximum LAI, from Equation (12); *A*_0sum_ is the annual total potential GPP; fAPAR_max_ is the seasonal maximum fAPAR, from Equation (11); and *σ* represents the extent to which seasonal LAI dynamics depart from a “square wave” whereby maximum LAI would be maintained over the whole growing season (Xia et al. 2015; Zhou et al. 2023). To estimate *σ* we used data at flux sites to obtain “observed” values of *m*, as the ratio of annual average (satellite‐derived) LAI to annual mean (flux tower‐derived) GPP (Equation 17). Then *σ* was calculated according to Equation (20) with “observed” *m* values, satellite‐derived seasonal maximum LAI and fAPAR, and “observed” *A*_0_ as inputs. The “observed” total *A*_0_ is the ratio of flux tower‐derived GPP to satellite‐based fAPAR (data sources are described below). This method yielded *σ* = 0.771 as a global estimate.

### 2.2 Data and Model Evaluation

#### 2.2.1 Flux‐Tower Data and Site‐Based Analysis

GPP and meteorological data at flux‐tower sites were obtained from the FLUXNET 2015 Tier Two and ONEFLUX datasets (http://fluxnet.fluxdata.org/) (Pastorello et al. 2020). At the site level, the sub‐daily P model (Mengoli et al. 2022) was used to calculate potential GPP with the FLUXNET datasets providing the meteorological variables required as input: incident photosynthetic photon flux density (PPFD_IN, μmol m^−2^ s^−1^), vapor pressure deficit (VPD_F, Pa), air temperature (TA_F, °C), and carbon dioxide mole fraction (CO_2__F_MDS, μmol mol^−1^) on a half‐hourly timestep. In contrast with the standard P model (Stocker et al. 2020), the sub‐daily P model (Mengoli et al. 2022) implements acclimation of photosynthetic parameters to midday conditions, when the light is greatest, and explicitly separates the subdaily (fast) responses of photosynthesis and stomatal conductance from slower acclimated responses to environmental variations over a 15‐day period. The effect of soil moisture stress on simulated GPP was considered separately by applying an additional empirical soil moisture limitation function (Mengoli et al. 2023).

Some days of climate data are missing from some sites due to quality control issues, so simulated GPP cannot be calculated for those days. Considering that the fAPAR_max_ model (Equation 11) (Zhu et al. 2023; Cai et al. 2025) requires annual total potential GPP as input, only those site‐years with > 300 daily GPP simulations were used for analysis from a total of 149 flux tower sites. We used data in the time range from 2001 to 2018 (1038 site‐years), including 47 evergreen needleleaf forest (ENF) sites, 14 evergreen broadleaf forest (EBF) sites, 25 deciduous broadleaf forest (DBF) sites, seven mixed forest (MF) sites, two closed shrubland (CSH) sites, seven open shrubland (OSH) sites, six woody savanna (WSA) sites, six savanna (SAV) sites, and 31 grassland (GRA) sites (Table S1).

#### 2.2.2 Gridded Climate Data and Global Analysis

We used the standard P model (Stocker et al. 2020) for the global analysis. Six‐hourly precipitation, maximum, minimum, and mean temperature, atmospheric pressure, and surface downwelling shortwave radiation at 0.5 resolution were downloaded from the CRU‐JRA dataset version 2.5 (Harris 2024). This is the climate forcing used in the TRENDY project (Sitch et al. 2015, 2024). The hourly data were converted to daily timescales, as inputs of the P model, by averaging the 24‐hourly data. VPD was calculated using maximum and minimum temperature and water vapor pressure. Solar radiation was converted to incident PPFD assuming a flux: energy ratio of 4.6 μmol J^−1^ and a photosynthetically active fraction of 0.5. We downloaded globally averaged monthly mean CO_2_ concentrations (μmol mol^−1^) from NOAA Global Monitoring Laboratory for 2001–2019 (NOAA/GML; https://gml.noaa.gov/ccgg/trends/; accessed November 2023) and then interpolated them into daily scale.

The effect of soil moisture stress on simulated GPP was represented using the empirical ‘penalty factor’ developed by Stocker et al. (2020), with the annual time course of soil moisture calculated using the Simple Process‐Led Algorithms for Simulating Habitats (SPLASH) model (version 1: Davis et al. 2017). As the standard P model makes separate calculations for GPP of plants following the C_3_ or C_4_ photosynthetic pathways, a dynamic C_4_ vegetation fraction was simulated based on a C_3_/C_4_ competition model (https://pyrealm.readthedocs.io/en/latest/) embedded in the P model. This model requires tree cover percentages as inputs, which were derived from MODIS MOD44B v006 during 2001–2019 (DiMiceli 2015). Areas where cropland cover is > 50% were excluded from the C_3_/C_4_ vegetation fraction map. Cropland cover data at 0.05° resolution from 2001 to 2019 were derived from MODIS MCD12C1 v006 (Friedl and Sulla‐Menashe 2015).

#### 2.2.3 Satellite LAI Data (Model Evaluation Data)

We used LAI time series derived from two alternative datasets (MODIS and Copernicus LAI) to evaluate the model's performance. MODIS LAI data were derived from the MODIS MOD15A2H Leaf Area Index/FPAR product, given at a resolution of 0.05 at a daily timestep from 2001 to 2019 (Li et al. 2023). Copernicus LAI data were derived from Copernicus Global Land Products (https://gbov.acri.fr) for the same period at 1 km resolution. The results described below are based on the MODIS data product. Results of model evaluation based on the Copernicus data are shown in Table S4; Figures S2 and S3.

#### 2.2.4 Simulated LAI Data From the TRENDY Project

We downloaded simulated LAI data during 2001–2019 from the ensemble of 15 TRENDY ecosystem models (TRENDY‐v9; Table 1). The S2 simulations were used, in which identical, time‐varying climate and CO_2_ are prescribed to all the models. The LAI datasets from different models were regridded to 0.5° resolution using the first‐order conservative remapping function (remapcon) from the Climate Data Operators (CDO) software package (https://code.mpimet.mpg.de/projects/cdo).

**TABLE 1:** Details of the models from TRENDY‐v9.

Model name	Spatial resolution	Reference
CABLE‐POP	1° × 1°	Haverd et al. (2018)
CLASSIC	2.8125° × 2.8125°	Melton et al. (2020)
CLM 5.0	0.9375° × 1.25°	Lawrence et al. (2019)
IBIS	1° × 1°	Yuan et al. (2014)
ISAM	0.5° × 0.5°	Meiyappan et al. (2015)
ISBA‐CTRIP	1° × 1°	Delire et al. (2020)
JSBACH	1.875° × 1.875°	Reick et al. (2021)
JULES‐ES	1.25° × 1.875°	Wiltshire et al. (2021)
LPJ‐GUESS	0.5° × 0.5°	Smith et al. (2014)
LPX‐Bern	0.5° × 0.5°	Lienert and Joos (2018)
OCN	1° × 1°	Zaehle and Friend (2010)
ORCHIDEEv3	0.5° × 0.5°	Vuichard et al. (2019)
SDGVM	1° × 1°	Walker et al. (2017)
VISIT	0.5° × 0.5°	Kato et al. (2013)
YIBs	1° × 1°	Yue and Unger (2015)

#### 2.2.5 Evaluation Methods

**FIGURE 2:**
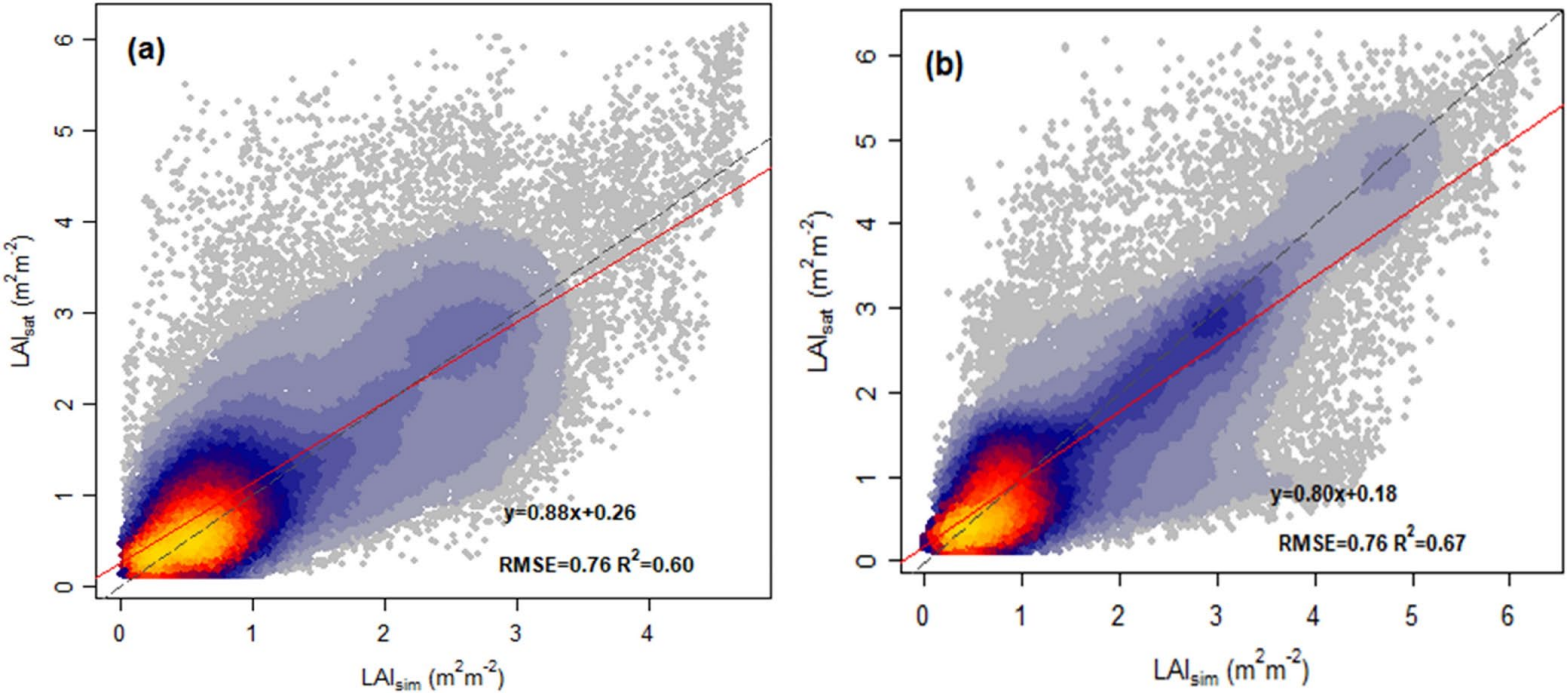
Correlation of observed and modeled 7‐day mean LAI values for all sites pooled. (a) Model_Prognostic_: Simulated LAI dynamics using P model‐derived GPP and simulated annual peak LAI from the fAPAR_max_ model as inputs. (b) Model_Flux_: Simulated LAI dynamics using flux tower‐derived GPP and annual peak satellite LAI as inputs. Grey lines are the 1:1 line; red lines are the regression lines.

**FIGURE 3:**
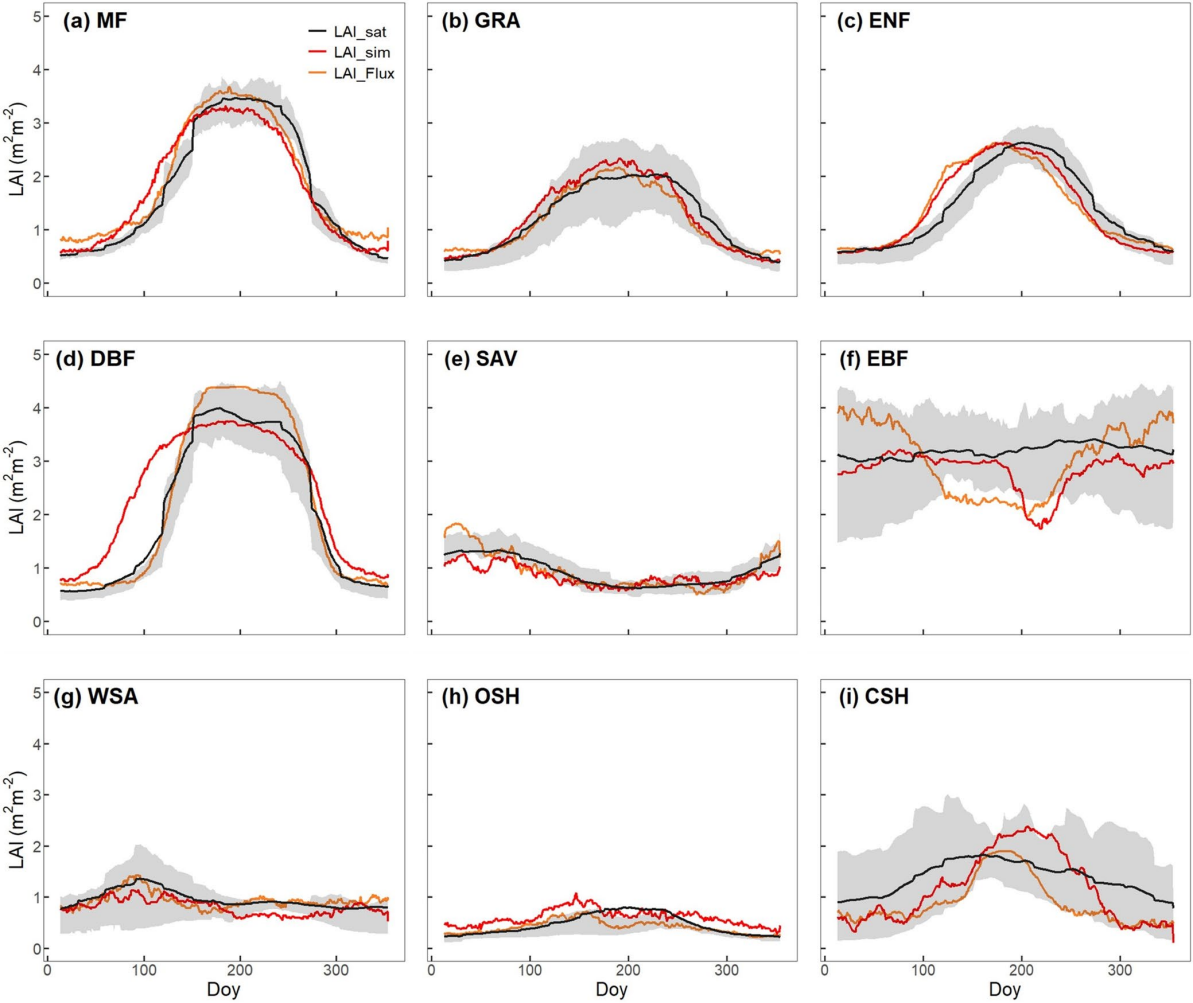
Mean seasonal cycles of LAI by biomes. Observations are shown by the black line and grey band, representing the median and 33/66% quantiles of all data (multiple sites and years) pooled by biome. The red line (LAI_sim_) is the seasonal variation of simulated LAI across all sites and years for each biome, where simulated LAI is calculated from Model_Prognostic_ using P model derived‐GPP and annual peak LAI from the fAPAR_max_ model as inputs. The orange line (LAI_Flux_) is the seasonal variation of simulated LAI across all sites and years for each biome, calculated from Model_Flux_, where simulated LAI is calculated using flux tower‐derived GPP and annual peak MODIS LAI as inputs. (a) MF, mixed forest; (b) GRA, grassland; (c) ENF, evergreen needleaf; (d) DBF, deciduous broadleaf; (e) SAV, Savanna; (f) EBF, evergreen broadleaf; (g) WSA, woody savanna; (h) OSH, open shrubland; (i) CSH, closed shrubland.

The LAI model depends on several components, including P model‐derived GPP dynamics and the prediction of seasonal maximum fAPAR and LAI. We conducted multiple sets of simulations at flux sites to investigate the dependence of model performance on alternative model setups. Two model combinations were defined: (1) Model_Flux_: Inputs are *A*_0_, which is derived by dividing flux‐derived GPP by satellite‐derived fAPAR (Figure 2b; Table 3; Figure 3) and the seasonal maximum LAI, also obtained from the remote sensing data; (2) Model_Prognostic_: Inputs are P model‐derived *A*_0_ and predicted seasonal maximum LAI from Equation (12) (Figure 2a; Table 3; Figure 3). Model_Flux_ is used to evaluate the LAI model framework separately, and Model_Prognostic_ assesses the overall performance of the integration of the LAI from the P model (Stocker et al. 2020) and the fAPAR_max_ model (Cai et al. 2025).

**FIGURE 4:**
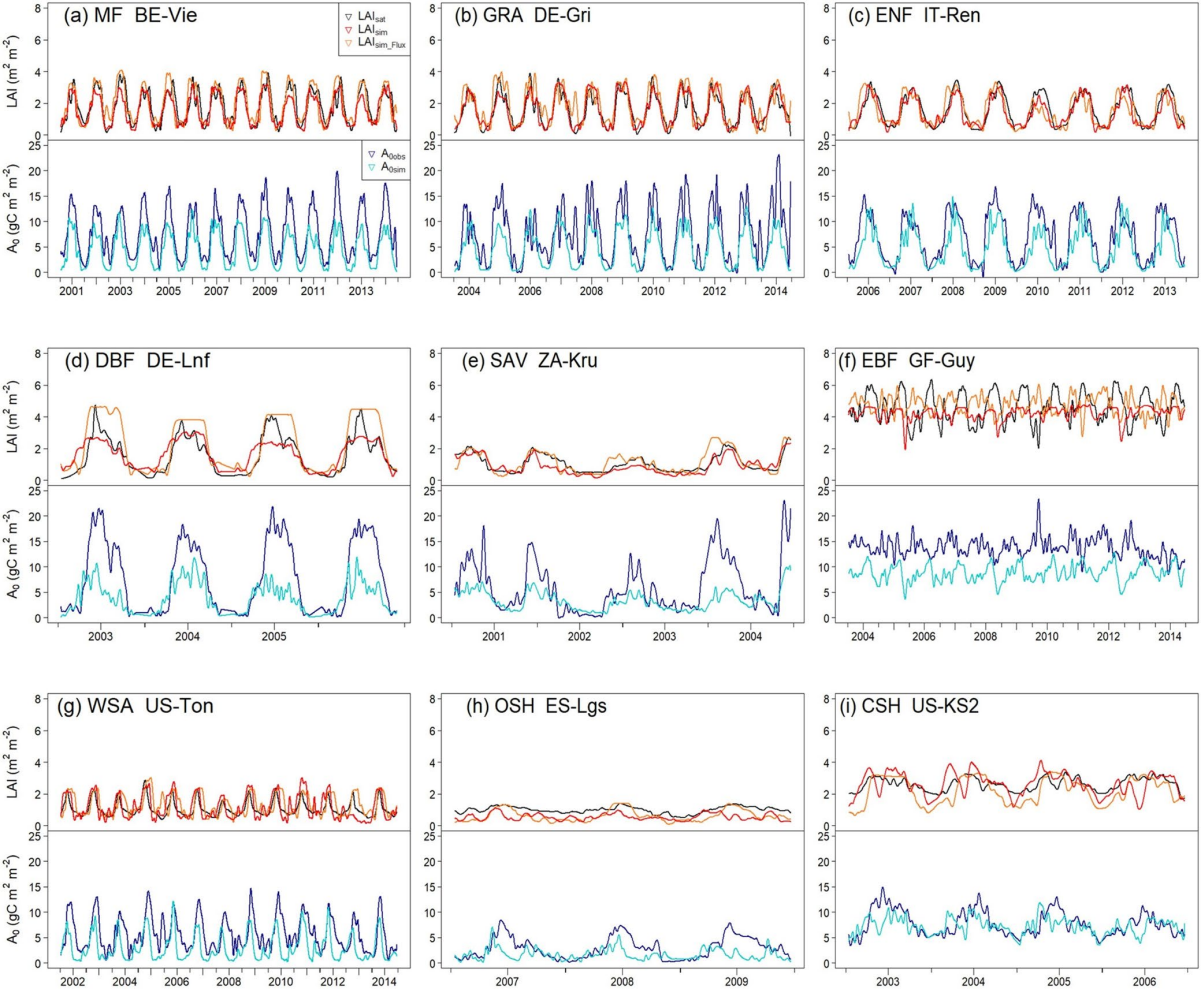
Representative time series of predicted and observed LAI and potential GPP in different biomes. LAI_sat_, Satellite LAI; LAI_sim_, Simulated LAI dynamics using P model‐derived GPP and simulated annual peak LAI from the fAPAR_max_ model as inputs (Model_Prognostic_); LAI_sim_Flux_, Simulated LAI dynamics using flux tower‐derived GPP and annual peak satellite LAI as inputs (Model_Flux_); *A*_0obs_: Observed potential GPP at flux sites (g C m^−2^ day^−1^); *A*_0sim_: Simulated potential GPP at flux sites (g C m^−2^ day^−1^). (a) MF, mixed forest; (b) GRA, grassland; (c) ENF, evergreen needleaf; (d) DBF, deciduous broadleaf; (e) SAV, savanna; (f) EBF, evergreen broadleaf; (g) WSA, woody savanna; (h) OSH, open shrubland; (i) CSH, closed shrubland.

**FIGURE 5:**
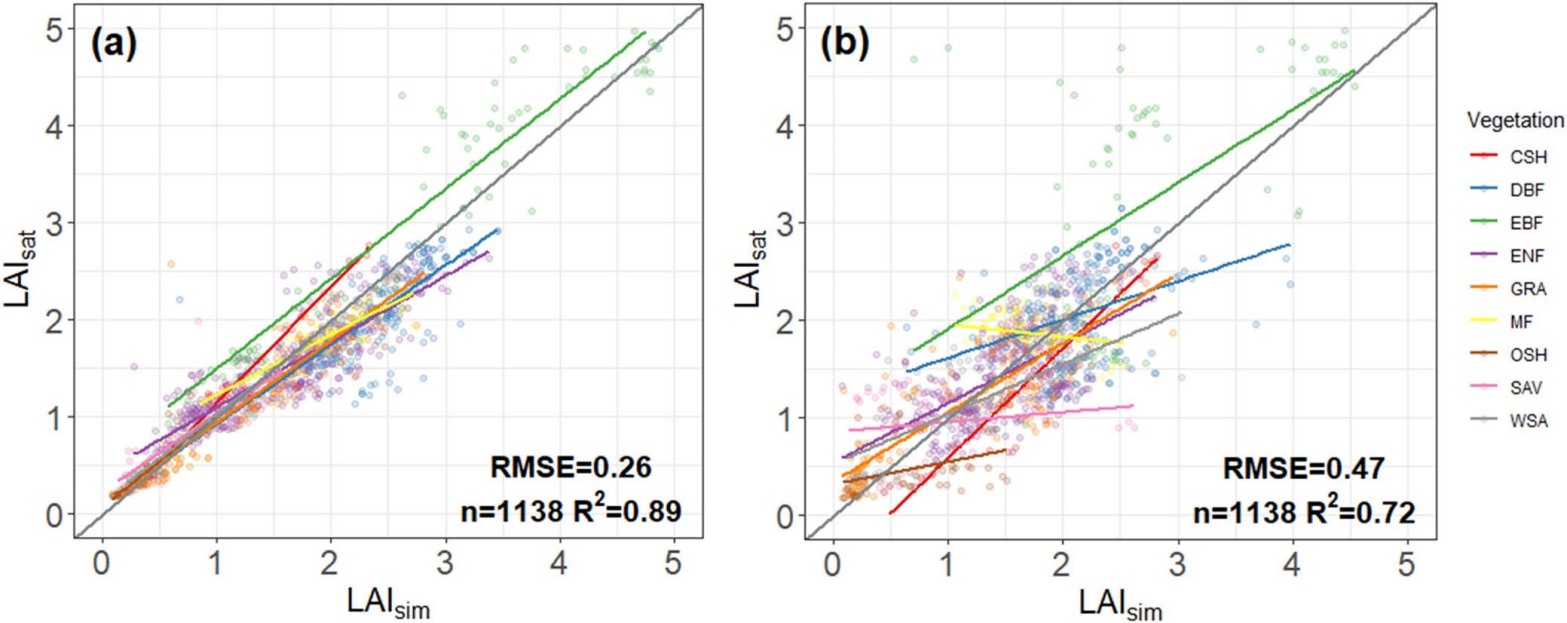
Regression analysis for (a) satellite‐derived versus simulated annual average LAI, where simulated LAI is calculated using flux tower‐derived GPP and annual peak MODIS LAI as inputs (Model_Flux_); (b) satellite‐derived versus simulated annual average LAI, where simulated LAI is calculated using P model‐derived GPP and annual peak LAI from the fAPAR_max_ model as inputs (Model_Prognostic_). Grey lines are the 1:1 lines, and coloured lines are regressions based on all sites within each biome. *R* ^2^ and RMSE are based on pooled data for all biomes. CSH, closed shrubland; DBF, deciduous broadleaf; EBF, evergreen broadleaf; ENF, evergreen needleleaf; GRA, grassland; MF, mixed forest; OSH, open shrubland; SAV, savanna; WSA, woody savanna.

We analyzed spatial (multi‐year mean values by site), annual, seasonal (mean by month of year), and weekly (mean by week of year) variability for both model combinations (Table 3), pooling data by biomes (Table S3). The models' performance in simulating intra‐ and inter‐annual LAI variability was also evaluated for different biomes (Figures 3 and 4). We separately analyzed annual mean LAI (Figure 5a,b) on an annual scale. We also evaluated the performance of our model and 15 other models participating in the TRENDY project in predicting multi‐year average LAI (2001–2019), annual average LAI time series, and seasonal LAI variation by comparing with the LAI products derived from MODIS (Li et al. 2023).

We calculated *R* ^2^, root mean squared error (RMSE), relative root mean squared error (RRMSE), the Pearson correlation coefficient (*r*), and the mean bias for model evaluation. RRMSE is the ratio of the root‐mean‐squared error to the mean of observed values, which normalizes RMSE by the target variable range and presents it as a percentage for easy cross‐biome comparison. More accurate models are expected to have higher correlation coefficients and smaller RMSEs, RRMSEs, and bias errors when compared with data. Analyses were conducted in the open‐source environments R (version 4.1.0) and Python (version 3.8.10).

#### 2.2.6 Parameter Sensitivity Tests

For simplicity, in our main analysis, we fitted a single value for the parameter *σ* across all sites and years (Table 2). However, we also investigated the variation of *σ* with the environment (Table S8). Additionally, given the potential effects of satellite data uncertainty on *σ*, we fitted *σ* at flux sites based on MODIS and Copernicus data separately (Table S7). Based on the P model derived GPP and the fAPAR and LAI products of MODIS, GLOBMAP, and AVHRR, we further explored the spatial variation of *σ* on a global scale (Figure S4) to assess its sensitivity to input GPP and satellite data.

**TABLE 2:** Definitions and expressions/values of parameters used in our LAI model.

Parameters	Definition	Values/Expressions	Variability with time	Variability with space
*f*_0_	The ratio of precipitation to transpiration (dimensionless)	$f_{0}=0.65\times e^{-b\times \ln^{2}\left(\frac{\mathrm{AI}}{1.9}\right)}$	Yes	Yes
*z*	The unit cost of constructing and maintaining leaves	12.227 mol m^−2^ a^−1^	No	No
*σ*	The extent to which seasonal LAI dynamics depart from a “square wave” whereby maximum LAI would be maintained over the whole growing season (dimensionless)	0.771	No	No
*m*	The ratio of steady‐state LAI divides steady‐state GPP	$m=\left(\sigma \times \mathrm{GSL}\times {\mathrm{LAI}}_{\max}\right)/\left(A_{0\mathrm{sum}}\times {\text{fAPAR}}_{\max}\right)$	Yes	Yes
*α*	The constant smoothing factor	0.067	No	No
*k*	Light extinction coefficient	0.5	No	No

*Note:* AI: The ratio of mean annual potential evapotranspiration to mean annual precipitation calculated using climate data for a 20‐year period. *b*: A constant value, equaling 0.604169. GSL, The length of the continuous period above 0°C longer than 5 days. LAI_max_, The seasonal maximum LAI. *A*_0sum_, The annual total potential GPP. fAPAR_max_, The seasonal maximum fAPAR.

The light extinction coefficient (*k*), used to convert from fAPAR to LAI, could be affected by canopy structure and therefore vary between vegetation types. However, Cai et al. (2025) have tested using a value that varies as a function of biome or a pixel‐specific value compared to using a constant *k* value and have shown that this has no significant impact on the prediction of the seasonal maximum LAI. We therefore use a constant *k* value of 0.5 in this analysis.

## 3 Results

### 3.1 LAI Variability Across Scales

The LAI model can capture the observed pattern of variability in MODIS‐derived LAI across temporal scales (Table 3, Figure 2). It explains 67% of the variance in LAI with data aggregated to seven‐day means (53,863 data points) when driven by flux tower‐derived GPP (Model_Flux_), and 60% of variance when driven by the P model‐derived GPP (Model_Prognostic_). Seasonal and annual variations are also well simulated (*R* ^2^: 70% to 87% for Model_Flux_ and 67%–70% for Model_Prognostic_). Still, more variance is explained for LAI across sites, with 90% for Model_Flux_ and 72% for Model_Prognostic_. Site‐level interannual variations are less well simulated (*R* ^2^: 43% for Model_Flux_, and 34% for Model_Prognostic_) (Figure S5). Our LAI model (Model_Prognostic_) performs slightly less well when compared with the alternative Copernicus‐derived LAI. Nevertheless, it still accounts for 48%, 51%, 63%, and 45% of the variance in Copernicus‐derived LAI aggregated to seven‐day means, monthly mean, annual mean, and site mean, respectively (Table 4; Table S4; Figures S2 and S3).

**TABLE 3:** Regression coefficient (Slope), *R* ^2^ and RMSE of simulated and MODIS‐derived LAI (Li et al. 2023) assessed at different timescales.

	Model_Prognostic_			Model_Flux_			*N*
	Slope	*R* ^2^	RMSE	Slope	*R* ^2^	RMSE	
7 days	0.88	0.60	0.76	0.80	0.67	0.76	53,863
Seasonal	0.99	0.67	0.79	0.84	0.70	0.68	13,184
Annual	0.96	0.70	0.61	0.90	0.87	0.29	1138
Spatial	0.98	0.72	0.47	0.93	0.90	0.26	163

*Note:* Model_Prognostic_: Simulated LAI dynamics using P model‐derived GPP and simulated annual peak LAI from the fAPAR_max_ model as inputs. Model_Flux_: Simulated LAI dynamics using flux tower‐derived GPP and annual peak satellite LAI as inputs.

**TABLE 4:** Satellite‐derived versus modeled seven‐day mean leaf area index (LAI) by biome.

Biome	(a) Model_Flux_				(b) Model_Prognostic_ (Copernicus)				(c) Model_Prognostic_ (MODIS)				*N*
	*r*	RRMSE	*R* ^2^	Bias	*r*	RRMSE	*R* ^2^	Bias	*r*	RRMSE	*R* ^2^	Bias	
ENF	0.57	30.05	0.39	0.20	0.63	25.42	0.38	0.11	0.72	24.76	0.55	0.17	18,489
EBF	0.75	16.37	0.56	0.23	0.60	20.10	0.46	0.42	0.62	22.10	0.27	0.48	3584
DBF	0.78	22.87	0.79	−0.13	0.71	32.71	0.53	0.27	0.78	28.13	0.48	−0.12	8434
MF	0.70	18.84	0.58	0.12	0.73	25.36	0.55	0.29	0.79	27.92	0.54	0.41	3855
OSH	0.59	30.87	0.51	0.07	0.35	52.92	0.11	−0.01	0.58	50.10	0.21	−0.05	2448
CSH	0.83	15.24	0.74	0.14	0.43	21.79	0.15	0.32	0.66	27.29	0.36	−0.02	950
WSA	0.69	26.01	0.44	0.06	0.58	50.46	0.41	−0.35	0.71	33.13	0.46	0.32	2771
SAV	0.52	27.36	0.38	0.15	0.32	46.11	0.15	−0.21	0.30	36.21	0.04	0.08	1934
GRA	0.72	27.30	0.69	0.04	0.70	32.05	0.51	0.06	0.77	26.33	0.63	0.15	9300
Overall	0.78	23.49	0.67	0.10	0.67	30.32	0.48	0.09	0.75	27.41	0.60	0.16	53,863

*Note:* (a) Model_Flux_ (MODIS): Inputs are flux‐derived *A*_0_ and annual peak LAI obtained from the MODIS data; (b) Model_Prognostic_: Inputs are P model‐derived *A*_0_ and predicted annual peak LAI from Equation (12), with Copernicus‐derived LAI as a benchmark. (c) Model_Prognostic_: Inputs are P model‐derived *A*_0_ and predicted annual peak LAI from Equation (12), with MODIS‐derived LAI as a benchmark. Reported metrics are the Pearson correlation coefficient (*r*), relative root mean square error (RRMSE), *R* ^2^ and bias.

Abbreviations: CSH, closed shrubland; DBF, deciduous broadleaf; EBF, evergreen broadleaf; ENF, evergreen needleleaf; GRA, grassland; MF, mixed forest; OSH, open shrubland; SAV, Savanna; WSA, woody savanna.

The LAI model also captures the seasonal and interannual variations of annual LAI for each biome (Figures 3 and 4, Table 4). The LAI simulated by Model_Flux_ (Figure 3) agrees well with MODIS‐derived LAI, especially for MF (*R* ^2^ = 58%; RRMSE = 18%), GRA (*R* ^2^ = 69%; RRMSE = 27%), DBF (*R* ^2^ = 79%; RRMSE = 22%), EBF (*R* ^2^ = 56%; RRMSE = 23%) and WSA (*R* ^2^ = 44%; RRMSE = 26%) and CSH (*R* ^2^ = 72%; RRMSE = 15%). Observed LAI dynamics for ENF lag simulated LAI, with a delay of up to 1 month in spring (*R* ^2^ = 39%; RRMSE = 15%). The ability of the model to simulate LAI in arid biomes (tropical savanna, open shrubland) was relatively poor; nonetheless, the model still accounted for 38% and 51% of the variance in satellite‐derived LAI, with RRMSEs of 27% and 30%, respectively, in these biomes. Compared to the LAI model driven by flux tower‐derived GPP (Model_Flux_), the simulated LAI using P model‐derived GPP (Model_Prognostic_) tends to overestimate LAI in early spring, especially for MF (*R* ^2^ = 54%; RRMSE = 27%), GRA (*R* ^2^ = 63%; RRMSE = 26%), ENF (*R* ^2^ = 55%; RRMSE = 2 4%) and DBF (*R* ^2^ = 48%; RRMSE = 28%). This bias reflects the P model's known tendency to overestimate GPP in the early part of the growing season, probably because it does not take into account the time required for new leaves to become fully functional under conditions of high light and low temperature (Stocker et al. 2020; Luo et al. 2023; Figure S7).

The capability of our LAI model (Model_Prognostic_) to capture seasonal changes in Copernicus‐derived LAI for each biome is inferior to that of MODIS‐derived LAI, but there are not big differences (Table 4; Table S4; Figures S2 and S3). The underestimation of *A*_0_ (but not LAI) at some savanna sites (Figure 4e and Figure S7) is likely because the sub‐daily model does not consider C_4_ photosynthesis (Mengoli et al. 2022). The underestimation of *A*_0_ at dense forests, such as DBF and EBF (Figure S7), is due to the inherent limitations of satellite data, as satellite‐derived fAPAR of different products varies greatly at dense forests (Stocker et al. 2020).

### 3.2 Annual Mean LAI at Flux Sites

**FIGURE 6:**
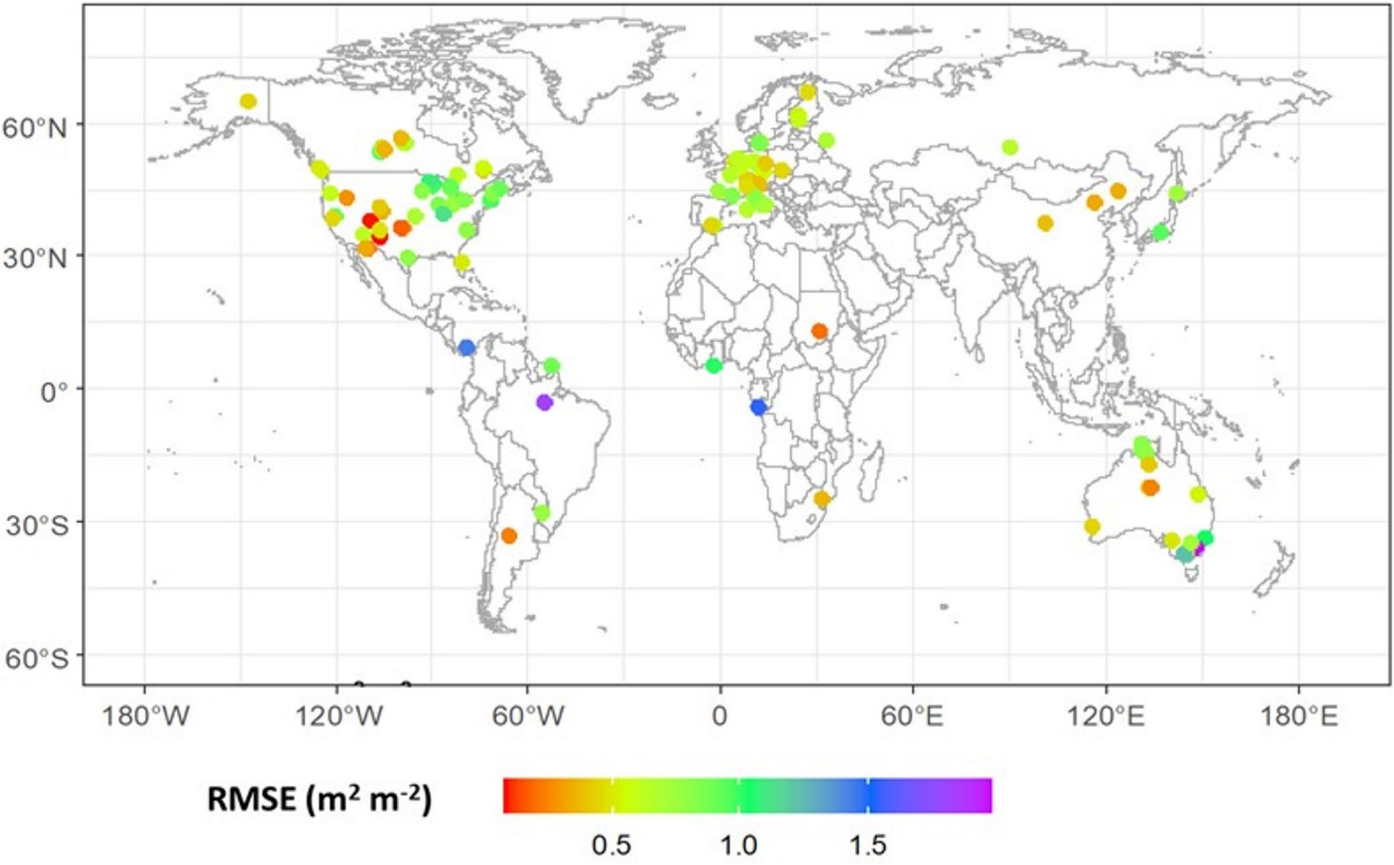
The spatial distribution of root mean square error (RMSE) between satellite‐derived and modeled LAI from Model_Prognostic_ at each flux tower site. Map lines delineate study areas and do not necessarily depict accepted national boundaries.

Model_Prognostic_ tends to overestimate annual mean LAI in more arid biomes (Figure 5b) compared to Model_Flux_ (Figure 5a), likely due to higher predicted seasonal maximum LAI in GRA and OSH (Figures 3 and 4). Conversely, the underestimation of annual mean LAI for EBF and DBF is likely caused by a lower predicted seasonal maximum LAI and *A*_0_ (Figures 3 and 4). The RMSE between satellite‐derived and modeled LAI (Figure 5) is < 0.5 m^2^m^−2^ for 45% of the sites, < 1 m^2^ m^−2^ for 90% of the sites, and < 1.5 m^2^ m^−2^ at 98% of the sites. There are four sites where the RMSE exceeds 1.5 m^2^ m^−2^: one SAV site (CG‐Tch, RMSE = 1.69 m^2^ m^−2^) and three EBF sites (AU‐Tum: 2.19 m^2^ m^−2^; BR‐Sa3: 1.87 m^2^ m^−2^; AU‐Wac: 1.58 m^2^ m^−2^; Figure 6.

### 3.3 Global‐Scale Modeling

**FIGURE 7:**
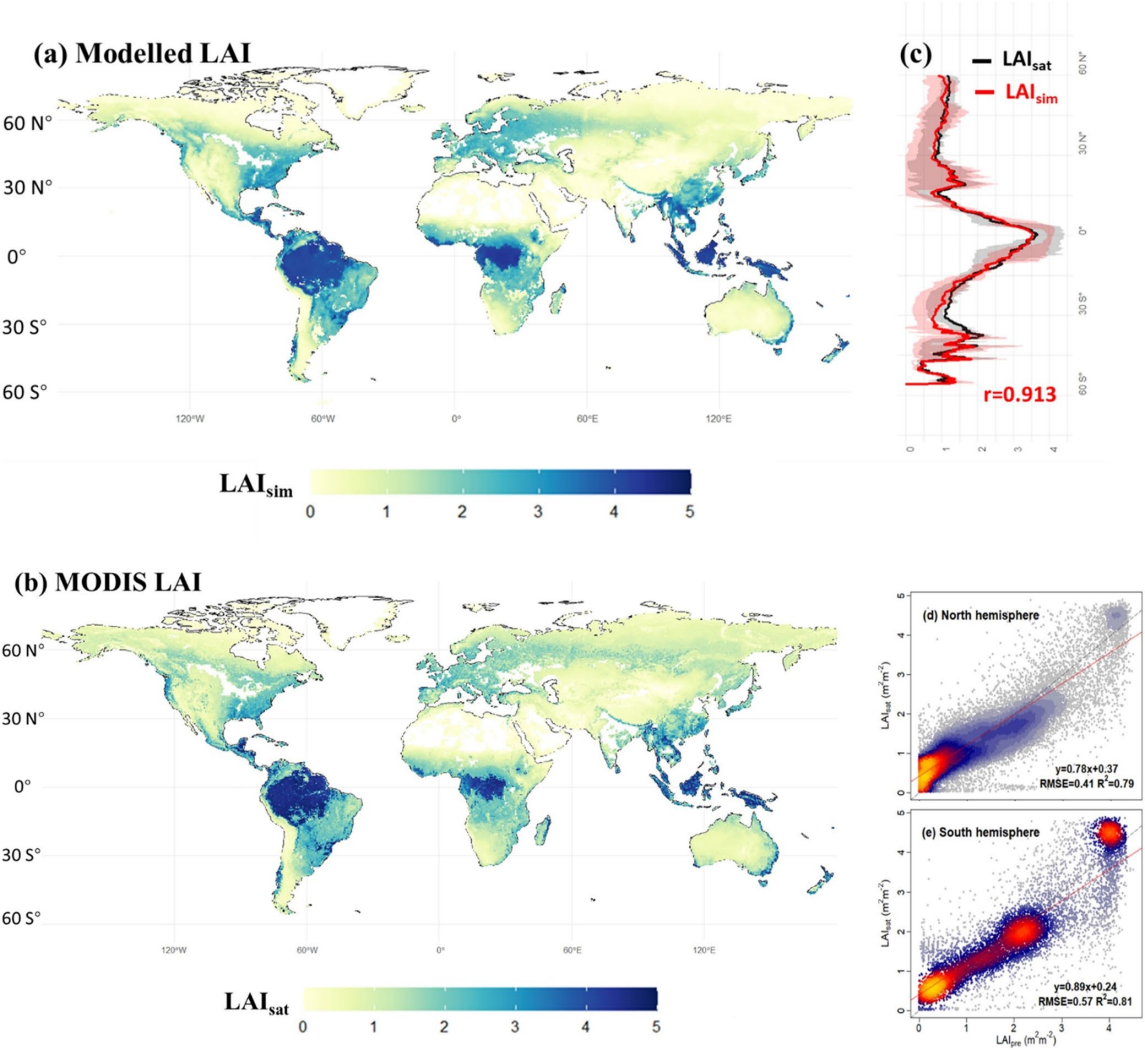
Spatial distributions of multi‐year average LAI (2000–2019) from (a) our modeled results and (b) MODIS products, and (c) their latitudinal variation. Regressions are shown for the comparisons between our modeled results and MODIS products in the northern (d) and southern (e) hemispheres. The red and grey lines in (d–e) are the regression lines and 1:1 line, respectively. Areas dominated by croplands snow/ice, and non‐vegetated areas are shown in white in panels (a, b).

The LAI model presented here, based on the close relationship between steady‐state GPP and LAI, successfully captures MODIS‐derived multi‐year average LAI over 2001–2019 across most of the global land surface covered (or partly covered) by natural vegetation. It somewhat underestimates LAI in Amazonia, North America, and northeastern Asian temperate deciduous forest, and overestimates LAI in arid regions including parts of southern Africa and South America (Figure 7 and Figures S6 and S9). The simulated results are highly consistent with the latitudinal patterns shown in MODIS‐derived products with multi‐year mean LAI, with a correlation coefficient of 0.91 (Figure 7c,d). The seasonal time course of LAI on a global scale is also well simulated, apart from the “early spring effect” in the northern hemisphere, and a slight underestimation of global LAI in January and July (Figure S10).

Our model is better than the 15 TRENDY models (Table S9) at predicting multi‐year average MODIS‐derived LAI for 2001–2019 (Figure S11), with an RMSE of 0.46 and an *R* ^2^ of 0.84 compared to RMSE values between 0.48 and 0.87 and *R* ^2^ values between 0.34 and 0.79 for the TRENDY models. ISBA‐CTRIP is the best‐performing TRENDY model in terms of RMSE (0.48) and *R* ^2^ (0.79) but has a poorer slope coefficient (Figure S11). JSBACH has a better slope coefficient than our model, but worse RMSE (0.60) and *R* ^2^ (0.68) scores (Figure S11). Our model also performs better in terms of annual average LAI time series (Figures S12–S14), with a mean RRMSE of 46.14 compared to a range from 47.30 to 256.19. The two best‐performing TRENDY models are again, ISBA‐CTRIP (RRMSE = 49.67) and JSBACH (RRMSE = 47.30). Our model also reproduces the seasonal variations of LAI well (Figures S15–S17) compared to the TRENDY models, with a mean RRMSE of 56.14 compared to the TRENDY range of 63.11–257.76. Our model also performs better than the TRENDY models when using the Copernicus‐derived LAI as a benchmark, with higher *R* ^2^ and lower RMSE and RRMSE (Figures S18–S26).

### 3.4 Effects of Parameter Variation

**FIGURE 8:**
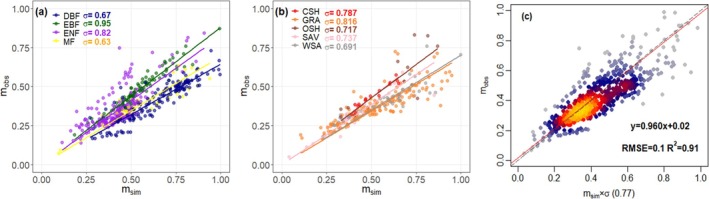
Variation of the parameter *m* for different biomes (a, b), and all biomes together (c). CSH, closed shrubland; DBF, deciduous broadleaf; EBF, evergreen broadleaf; ENF, evergreen needleleaf; GRA, grassland; MF, mixed forest; OSH, open shrubland; SAV, savanna; WSA, woody savanna.

The predicted site‐level values of *m* capture the variation of observed values well (Slope = 0.90; *R* ^2^ = 91%; RRMSE = 46%) for all biomes together when *σ* is maintained as a constant (Figure 8c). When the potential variation of *σ* between different biomes is considered, separately fitted values of *σ* for EBF and ENF are higher (EBF: *σ* = 0.95; *R* ^2^ = 96%; RRMSE = 15%; ENF: *σ* = 0.82; *R* ^2^ = 88%; RRMSE = 37%) than those for DBF (*σ* = 0.67; *R* ^2^ = 89%; RRMSE = 59%) and MF (*σ* = 0.63; *R* ^*2*^ = 92%; RRMSE = 50%) (Figure 8a). CSH, GRA, OSH, SAV, and WSA show *σ* values in a relatively narrow range, from 0.69 to 0.82 (Figure 8b). Even though *σ* values decrease with temperature and increase with PPFD, these variations are small (Table S8). The *σ* values fitted using different satellite products at flux sites and global scales also differ very little (Tables S6 and S7; Figures S4 and S6). These results show that *σ* is a relatively robust parameter and can be used as a global constant to derive a general expression for predicting the quantitative relationship between steady‐state LAI and steady‐state GPP (Equation 20), applicable to all vegetation types.

## 4 Discussion

We have developed a universal global LAI model that predicts the daily time course of LAI from climate variables alone. The model is based on the theoretical approach introduced by Xin et al. (2018, 2020), which can be considered an eco‐evolutionary optimality hypothesis as it proposes that the dynamics of leaf display are closely coupled (with some time lag) to the time course of the productive capacity of leaves, which in turn is determined by light and weather conditions. We have combined this theoretical approach with a parsimonious and well‐tested GPP model, the P model (Wang, Lu, et al. 2017; Wang, Prentice, et al. 2017; Stocker et al. 2020; Mengoli et al. 2022), and a separately validated top‐down model to predict the seasonal maximum LAI (Zhu et al. 2023; Cai et al. 2025). We have also developed a semi‐empirical equation predicting the quantitative relationship between steady‐state LAI and steady‐state GPP, expressed as the parameter *m*, that is applicable across all biomes.

Our approach differs fundamentally from that of classical mechanistic phenological models, which focus on the triggers of phenological phase transitions (Chuine et al. 2000; Chuine and Régnière 2017; Chuine 2021). Mechanistic models can achieve high accuracy for predictions of the timing of phenological transitions at the species (Chuine 2010) and regional (Chuine et al. 2000) levels, but uncertainties about the drivers of key events and the need for species‐specific information mean that they are difficult to apply at a global scale. Our optimality‐based approach focuses on the emergent behaviour of leaves at the canopy scale, such that the timing of these phenological transitions is implicit rather than explicit and is better adapted for global vegetation and land‐surface modeling.

Many studies have demonstrated a positive correlation between LAI and photosynthetic activity at various temporal and spatial scales (Wu et al. 2016; Chen et al. 2020; Bian et al. 2003). Our results provide further support for the hypothesis of interdependence between the time course of LAI and the photosynthetic potential of leaves. This hypothesis provides a robust way to predict seasonal leaf allocation from the conditions supporting photosynthetic productivity, instead of trying to predict the timing of phenological events (McMaster and Wilhelm 1997; Badeck et al. 2004; Cleland et al. 2007). The resulting model is indifferent to the specific mechanisms used by plants in different environments to achieve optimality. The model also avoids explicitly simulating the dynamics of C allocation to leaf growth. One recent study found that leaf C accumulation accelerates in the early green‐up phase (due to both increased photosynthesis and a greater fraction of GPP allocated to leaves) and declines in the late green‐up phase (Meng et al. 2023). However, the theory of dynamic C allocation is incomplete (Hartmann et al. 2020) and there is insufficient information that would allow a time‐varying allocation fraction to be modeled globally. Relying on the interdependence of LAI time series and photosynthetic potential provides an effective alternative, as shown by Zhou et al. (2023) and in the present study.

Our LAI simulation framework tends to overestimate LAI values in spring in cold‐climate regions, due to a known deficiency of the P model (Stocker et al. 2020; Mengoli et al. 2022), which may be related to the fact that full photosynthetic activity lags behind leaf development in these environments (Croft et al. 2015; Luo et al. 2023). The model also tends to overestimate LAI slightly in grasslands and open vegetation in relatively dry climates. The effect of soil moisture on GPP in the P model is taken into account through an empirical soil moisture stress function (Stocker et al. 2020; Mengoli et al. 2022), in a similar way to many LSMs. This does not capture the impact of long‐term adjustments to maximum levels of GPP in more arid climates (Mengoli et al. 2023). The underestimation of LAI in tropical forests, where EBF is the dominant biome, is likely to be caused by problems with the satellite data (Xin et al. 2020) but may also reflect the extent to which the actual seasonal LAI dynamics deviate from the “square wave” approach that maintains maximum LAI throughout the growing season. Our LAI model tends to underestimate interannual variability in LAI in some biomes, probably because it lacks any “carry‐over” mechanism, such as soil moisture “memory” (Rahmati et al. 2024) or allocation of carbon to non‐structural carbohydrates (Ninomiya et al. 2023). Nevertheless, despite these shortcomings, the model performs well overall and better than the various models used in the TRENDY project.

The model as presented here has benefited from including a climatically variable *f*_0_ parameter, compared to the original formulation for the calculation of seasonal maximum fAPAR (Zhu et al. 2023; Cai et al. 2025). The parameter *f*_0_ refers to the fraction of antecedent precipitation that is used by plants, based on the hypothesis that plants adjust their rooting profiles in such a way as to access a certain fraction (*f*_0_) of annual precipitation. This carries the implication that the share of precipitation that evaporates directly (from foliage, or from bare soil) is regulated by vegetation properties. Although this implication is inconsistent with the approach usually taken in LSMs, there is ample support for it in the ecohydrological literature (Good et al. 2017). However, even if the variability of the *f*_0_ parameter with climate change is taken into account in the LAI model presented here, it still shows some tendency to overestimate LAI in arid regions. The parameter *z* in the fAPAR_max_ model (Zhu et al. 2023; Cai et al. 2025), which represents the unit cost of constructing and maintaining leaves, was fitted according to the chosen value of the light extinction coefficient (0.5). The *z* cost could vary with temperature or canopy structure (Cai et al. 2025), but a global constant was used here as a tradeoff between model complexity and effectiveness. These dependencies warrant further investigation, either from a theoretical perspective or through analyses of remotely sensed observations.

## Conflicts of Interest

The authors declare no conflicts of interest.

## Supporting information


Data S1.

## Data Availability

The data and code that support the findings of this study are openly available in Zenodo at https://doi.org/10.5281/zenodo.14919808. The flux tower dataset was obtained from FLUXNET at (http://fluxnet.fluxdata.org/) (FLUXNET2015). LAI and fAPAR data were obtained from the Copernicus Climate Change Service (C3S) Climate Data Store (CDS) at https://doi.org/10.24381/cds.7e59b01a.

## References

[gcb70125-bib-0002] Arora, V. K. , and G. J. Boer . 2005. “A Parameterization of Leaf Phenology for the Terrestrial Ecosystem Component of Climate Models.” Global Change Biology 11: 39–59. 10.1111/gcb.14310.

[gcb70125-bib-0003] Badeck, F.‐W. , A. Bondeau , K. Böttcher , et al. 2004. “Responses of Spring Phenology to Climate Change.” New Phytologist 162: 295–309. 10.1111/j.1469-8137.2004.01059.x.

[gcb70125-bib-0005] Bian, C. , J. Xia , X. Zhang , et al. 2003. “Uncertainty and Emergent Constraints on Enhanced Ecosystem Carbon Stock by Land Greening.” Journal of Advances in Modeling Earth Systems 15: e2022MS003397. 10.1029/2022MS003397.

[gcb70125-bib-0007] Caffarra, A. , A. Donnelly , and I. Chuine . 2011. “Modelling the Timing of *Betula pubescens* Budburst. II. Integrating Complex Effects of Photoperiod Into Process‐Based Models.” Climate Research 46: 159–170. 10.3354/cr00983.

[gcb70125-bib-0008] Cai, W. , and I. C. Prentice . 2020. “Recent Trends in Gross Primary Production and Their Drivers: Analysis and Modelling at Flux‐Site and Global Scales.” Environmental Research Letters 15: 124050. 10.1088/1748-9326/abc64e.

[gcb70125-bib-0102] Cai, W. , Z. Zhu , S. P. Harrison , et al. 2025. “A Unifying Principle for Global Greenness Patterns and Trends.” Communications Earth & Environment 6, no. 1: 19. 10.1038/s43247-025-01992-0.

[gcb70125-bib-0010] Cannell, M. , and R. Smith . 1983. “Thermal Time, Chill Days and Prediction of Budburst in *Picea sitchensis* .” Journal of Applied Ecology 20: 951–963. 10.2307/2403139.

[gcb70125-bib-0011] Castillioni, K. , G. S. Newman , L. Souza , and A. M. Iler . 2022. “Effects of Drought on Grassland Phenology Depend on Functional Types.” New Phytologist 236: 1558–1571. 10.1111/nph.18462. 36068954

[gcb70125-bib-0012] Chen, X. , F. Maignan , N. Viovy , et al. 2020. “Novel Representation of Leaf Phenology Improves Simulation of Amazonian Evergreen Forest Photosynthesis in a Land Surface Model.” Journal of Advances in Modeling Earth Systems 12: e2018MS001565. 10.1029/2018MS001565.

[gcb70125-bib-0100] Chen, C. , T. Park , X. Wang , et al. 2019. “China and India Lead in Greening of the World Through Land‐Use Management.” Nature Sustainability 2, no. 2: 122–129. 10.1038/s41893-019-0220-7. PMC6376198 30778399

[gcb70125-bib-0013] Chuine, I. 2010. “Why Does Phenology Drive Species Distribution?” Philosophical Transactions of the Royal Society, B: Biological Sciences 365, no. 1555: 3149–3160. 10.1098/rstb.2010.0142.2025. 20819809 PMC2981946

[gcb70125-bib-0014] Chuine, I. 2021. “A Unified Model for Budburst of Trees.” Journal of Theoretical Biology 207: 337–347. 10.1006/jtbi.2000.2178. 11082304

[gcb70125-bib-0015] Chuine, I. , G. Cambon , and P. Comtois . 2000. “Scaling Phenology From the Local to the Regional Level: Advances From Species‐Specific Phenological Models.” Global Change Biology 6: 943–952. 10.1046/j.1365-2486.2000.00368.x.

[gcb70125-bib-0016] Chuine, I. , and J. Régnière . 2017. “Process‐Based Models of Phenology for Plants and Animals.” Annual Reviews of Ecology, Evolution, and Systematics 48: 159–182. 10.1146/annurev-ecolsys-110316-022706.

[gcb70125-bib-0017] Cleland, E. E. , I. Chuine , A. Menzel , H. A. Mooney , and M. D. Schwartz . 2007. “Shifting Plant Phenology in Response to Global Change.” Trends in Ecology & Evolution 22: 357–365. 10.1016/j.tree.2007.04.003. 17478009

[gcb70125-bib-0018] Croft, H. , J. M. Chen , N. J. Froelich , B. Chen , and R. M. Staebler . 2015. “Seasonal Controls of Canopy Chlorophyll Content on Forest Carbon Uptake: Implications for GPP Modeling.” Journal of Geophysical Research: Biogeosciences 120: 1576–1586. 10.1002/2015JG002980.

[gcb70125-bib-0019] Currier, C. M. , and O. E. Sala . 2022. “Precipitation Versus Temperature as Phenology Controls in Drylands.” Ecology 103: e3793. 10.1002/ecy.3793. 35724971

[gcb70125-bib-0021] Davis, T. W. , I. C. Prentice , B. D. Stocker , et al. 2017. “Simple Process‐Led Algorithms for Simulating Habitats (SPLASH v.1.0): Robust Indices of Radiation, Evapotranspiration and Plant‐Available Moisture.” Geoscientific Model Development 10: 689–708. Zenodo. 10.5281/zenodo.376293.

[gcb70125-bib-0099] Delire, C. , R. Séférian , B. Decharme , et al. 2020. “The Global Land Carbon Cycle Simulated With ISBA‐CTRIP: Improvements Over the Last Decade.” Journal of Advances in Modeling Earth Systems 12, no. 9: e2019MS001886. 10.1029/2019ms001886.

[gcb70125-bib-0023] DiMiceli, C. M. , M. L. Carroll , R. A. Sohlberg , D. H. Kim , M. Kelly , and J. R. G. Townshend . 2015. “MOD44B MODIS/Terra Vegetation Continuous Fields Yearly L3 Global 250m SIN Grid V006.” In: NASA EOSDIS Land Processes DAAC. Accessed 2022‐08‐06. 10.5067/MODIS/MOD44B.006.

[gcb70125-bib-0024] Franklin, O. , J. Johansson , R. C. Dewar , et al. 2012. “Modeling Carbon Allocation in Trees: A Search for Principles.” Tree Physiology 32: 648–666. 10.1093/treephys/tpr138. 22278378

[gcb70125-bib-0101] Farquhar, G. D. , S. von Caemmerer , and J. A. Berry . 1980. “A Biochemical Model of Photosynthetic CO2 Assimilation in Leaves of C3 Species.” Planta 149, no. 1: 78–90. 10.1007/bf00386231. 24306196

[gcb70125-bib-0025] Friedl, M. , and D. Sulla‐Menashe . 2015. “MCD12C1 MODIS/Terra+Aqua Land Cover Type Yearly L3 Global 0.05Deg CMG V006.” In: NASA EOSDIS Land Processes DAAC. Accessed 2022‐08‐06. 10.5067/MODIS/MCD12C1.006.

[gcb70125-bib-0026] Fu, Y. , X. Li , X. Zhou , X. Geng , Y. Guo , and Y. Zhang . 2020. “Progress in Plant Phenology Modeling Under Global Climate Change.” Science in China: Earth Sciences 63: 1237–1247. 10.1007/s11430-019-9622-2.

[gcb70125-bib-0027] Fu, Y. H. , S. Piao , X. Zhou , X. Geng , F. Hao , and Y. Vitasse . 2019. “Short Photoperiod Reduces the Temperature Sensitivity of Leaf‐Out in Saplings of *Fagus sylvatica* but Not in Horse Chestnut.” Global Change Biology 25: 1696–1703. 10.1111/gcb.14599. 30779408

[gcb70125-bib-0028] Gibelin, A.‐L. , J.‐C. Calvet , J.‐L. Roujean , L. Jarlan , and S. O. Los . 2006. “Ability of the Land Surface Model ISBA‐A‐Gs to Simulate Leaf Area Index at the Global Scale: Comparison With Satellites Products.” Journal of Geophysical Research: Atmospheres 111, no. D18. 10.1029/2005JD006691.

[gcb70125-bib-0029] Good, S. P. , G. W. Moore , and D. G. Miralles . 2017. “A Mesic Maximum in Biological Water Use Demarcates Biome Sensitivity to Aridity Shifts.” Nature Ecology & Evolution 1: 1883–1888. 10.1038/s41559-017-0371-8. 29133901

[gcb70125-bib-0031] Harris, I. C. 2024. “CRU JRA v2.5: A Forcings Dataset of Gridded Land Surface Blend of Climatic Research Unit (CRU) and Japanese Reanalysis (JRA) Data; Jan.1901—Dec.2023.” NERC EDS Centre for Environmental Data Analysis. https://catalogue.ceda.ac.uk/uuid/43ce517d74624a5ebf6eec5330cd18d5.

[gcb70125-bib-0032] Harrison, S. P. , W. Cramer , O. Franklin , et al. 2021. “Eco‐Evolutionary Optimality as a Means to Improve Vegetation and Land‐Surface Models.” New Phytologist 231: 2125–2141. 10.1111/nph.17558. 34131932

[gcb70125-bib-0033] Hartmann, H. , M. Bahn , M. Carbone , and A. D. Richardson . 2020. “Plant Carbon Allocation in a Changing World–Challenges and Progress: Introduction to a Virtual Issue on Carbon Allocation.” New Phytologist 227: 981–988. 10.1111/nph.16757. 32662104

[gcb70125-bib-0104] Haverd, V. , B. Smith , L. Nieradzik , et al. 2018. “A New Version of the CABLE Land Surface Model (Subversion revision r4601) Incorporating Land Use and Land Cover Change, Woody Vegetation Demography, and A Novel Optimisation‐based Approach to Plant Coordination of Photosynthesis.” Geoscientific Model Development 11, no. 7: 2995–3026. 10.5194/gmd-11-2995-2018.

[gcb70125-bib-0037] Jolly, W. M. , R. Nemani , and S. W. Running . 2005. “A Generalized, Bioclimatic Index to Predict Foliar Phenology in Response to Climate.” Global Change Biology 11: 619–632. 10.1111/j.1365-2486.2005.00930.x.

[gcb70125-bib-0093] Kato, E. , T. Kinoshita , A. Ito , M. Kawamiya , and Y. Yamagata . 2013. “Evaluation of Spatially Explicit Emission Scenario of Land‐Use Change and Biomass Burning Using A Process‐based Biogeochemical Model.” Journal of Land Use Science 8, no. 1: 104–122. 10.1080/1747423x.2011.628705.

[gcb70125-bib-0106] Lawrence, D. M. , R. A. Fisher , C. D. Koven , et al. 2019. “The Community Land Model Version 5: Description of New Features, Benchmarking, and Impact of Forcing Uncertainty.” Journal of Advances in Modeling Earth Systems 11, no. 12: 4245–4287. 10.1029/2018ms001583.

[gcb70125-bib-0039] Lawrence, D. M. , K. W. Oleson , M. G. Flanner , et al. 2011. “Parameterization Improvements and Functional and Structural Advances in Version 4 of the Community Land Model.” Journal of Advances in Modeling Earth Systems 3: M03001. 10.1029/2011MS00045.

[gcb70125-bib-0040] Li, B. , Y. Ryu , C. Jiang , et al. 2023. “BESSv2.0: A Satellite‐Based and Coupled‐Process Model for Quantifying Long‐Term Global Land–Atmosphere Fluxes.” Remote Sensing of Environment 295: 113696. 10.1016/j.rse.2023.113696.

[gcb70125-bib-0097] Lienert, S. , and F. Joos . 2018. “A Bayesian Ensemble Data Assimilation to Constrain Model Parameters and Land‐use Carbon Emissions.” Biogeosciences 15, no. 9: 2909–2930. 10.5194/bg-15-2909-2018.

[gcb70125-bib-0045] Luo, Y. , A. Gessler , P. D'Odorico , K. Hufkens , and B. D. Stocker . 2023. “Quantifying Effects of Cold Acclimation and Delayed Springtime Photosynthesis Resumption in Northern Ecosystems.” New Phytologist 240: 984–1002. 10.1111/nph.19208. 37583086

[gcb70125-bib-0047] McMaster, G. S. , and W. W. Wilhelm . 1997. “Growing Degree‐Days: One Equation, Two Interpretations.” Agricultural and Forest Meteorology 87: 291–300. 10.1016/S0168-1923(97)00027-0.

[gcb70125-bib-0094] Meiyappan, P. , A. K. Jain , and J. I. House . 2015. “Increased Influence of Nitrogen Limitation on CO_2_ Emissions from Future Land Use and Land Use Change.” Global Biogeochemical Cycles 29, no. 9: 1524–1548. 10.1002/2015gb005086.

[gcb70125-bib-0105] Melton, J. R. , V. K. Arora , E. Wisernig‐Cojoc , et al. 2020. “CLASSIC v1.0: The Open‐source Community Successor to The Canadian Land Surface Scheme (CLASS) and the Canadian Terrestrial Ecosystem Model (CTEM) – Part 1: Model Framework and Site‐level Performance.” Geoscientific Model Development 13, no. 6: 2825–2850. 10.5194/gmd-13-2825-2020.

[gcb70125-bib-0048] Meng, F. , S. Hong , J. Wang , et al. 2023. “Climate Change Increases Carbon Allocation to Leaves in Early Leaf Green‐Up.” Ecology Letters 26: 816–826. 10.1111/ele.14205. 36958943

[gcb70125-bib-0049] Meng, L. , Y. Zhou , L. Gu , A. D. Richardson , J. Peñuelas , and Y. Fu . 2021. “Photoperiod Decelerates the Advance of Spring Phenology of Six Deciduous Tree Species Under Climate Warming.” Global Change Biology 27: 2914–2927. 10.1111/gcb.15575. 33651464

[gcb70125-bib-0050] Mengoli, G. , A. Agustí‐Panareda , S. Boussetta , S. P. Harrison , C. Trotta , and I. C. Prentice . 2022. “Ecosystem Photosynthesis in Land‐Surface Models. A First‐Principles Approach Incorporating Acclimation.” Journal of Advances in Modeling Earth Systems 14: e2021MS002767. 10.1029/2021MS002767.

[gcb70125-bib-0051] Mengoli, G. , S. P. Harrison , and I. C. Prentice . 2023. “A Global Function of Climatic Aridity Accounts for Soil Moisture Stress on Carbon Assimilation.” EGUsphere 1–19. 10.5194/egusphere-2023-1261.

[gcb70125-bib-0054] Ninomiya, H. , T. Kato , L. Végh , and L. Wu . 2023. “Modeling of Non‐Structural Carbohydrate Dynamics by the Spatially Explicit Individual‐Based Dynamic Global Vegetation Model SEIB‐DGVM (SEIB‐DGVM‐NSC Version 1.0).” Geoscientific Model Development 16: 4155–4170. 10.5194/gmd-16-4155-2023.

[gcb70125-bib-0055] Pastorello, G. , C. Trotta , E. Canfora , et al. 2020. “The FLUXNET2015 Dataset and the ONEFlux Processing Pipeline for Eddy Covariance Data.” Scientific Data 7: 225. 10.1038/s41597-020-0534-3. 32647314 PMC7347557

[gcb70125-bib-0057] Piao, S. , Q. Liu , A. Chen , et al. 2019. “Plant Phenology and Global Climate Change: Current Progresses and Challenges.” Global Change Biology 25: 1922–1940. 10.1111/gcb.14619. 30884039

[gcb70125-bib-0058] Prentice, I. C. , N. Dong , S. M. Gleason , V. Maire , and I. J. Wright . 2014. “Balancing the Costs of Carbon Gain and Water Transport: Testing a New Theoretical Framework for Plant Functional Ecology.” Ecology Letters 17: 82–91. 10.1111/ele.12211. 24215231

[gcb70125-bib-0059] Rahmati, M. , W. Amelung , C. Brogi , et al. 2024. “Soil Moisture Memory: State‐of‐the‐Art and the Way Forward.” Reviews of Geophysics 62: e2023RG000828. 10.1029/2023RG000828.

[gcb70125-bib-0060] Reaumur, R. D. 1735. “Observations du thermomètre faites à Paris pendant l'année 1735, comparées avec celles qui ont été faites sous la ligne, à l'Isle de France, à Alger et quelques unes de nos iles de l'Amérique.” Mémoires de Academie Royale Sciences: 545–576.

[gcb70125-bib-0107] Reick, C. H. , V. Gayler , D. Goll , et al. 2021. “JSBACH 3 – The Land Component of the MPI Earth System Model: Documentation of Version 3.2.” MPI Für Meteorologie. 10.17617/2.3279802.

[gcb70125-bib-0064] Schaphoff, S. , W. von Bloh , A. Rammig , et al. 2018. “LPJmL4—A Dynamic Global Vegetation Model With Managed Land—Part 1: Model Description.” Geoscientific Model Development 11: 1343–1375. 10.5194/gmd-11-1343-2018.

[gcb70125-bib-0065] Sierra, C. A. , V. Ceballos‐Núñez , H. Hartmann , D. Herrera‐Ramírez , and H. Metzler . 2022. “Ideas and Perspectives: Allocation of Carbon From Net Primary Production in Models Is Inconsistent With Observations of the Age of Respired Carbon.” Biogeosciences 19: 3727–3738. 10.5194/bg-19-3727-2022.

[gcb70125-bib-0066] Sitch, S. , P. Friedlingstein , N. Gruber , et al. 2015. “Recent Trends and Drivers of Regional Sources and Sinks of Carbon Dioxide.” Biogeosciences 12, no. 3: 653–679. 10.5194/bg-12-653-2015.

[gcb70125-bib-0067] Sitch, S. , M. O'Sullivan , E. Robertson , et al. 2024. “Trends and Drivers of Terrestrial Sources and Sinks of Carbon Dioxide: An Overview of the TRENDY Project.” Global Biogeochemical Cycles 38, no. 7: e2024GB008102. 10.1029/2024gb008102.

[gcb70125-bib-0109] Smith, B. , D. Wårlind , A. Arneth , et al. 2014. “Implications of Incorporating N Cycling and N Limitations on Primary Production in An Individual‐Based Dynamic Vegetation Model.” Biogeosciences 11, no. 7: 2027–2054. 10.5194/bg-11-2027-2014.

[gcb70125-bib-0071] Stocker, B. D. , H. Wang , N. G. Smith , et al. 2020. “P‐Model v1.0: An Optimality‐Based Light Use Efficiency Model for Simulating Ecosystem Gross Primary Production.” Geoscientific Model Development 13: 1545–1581. 10.5194/gmd-13-1545-2020.

[gcb70125-bib-0072] Swinehart, D. F. 1962. “The Beer‐Lambert Law.” Journal of Chemical Education 39: 333. 10.1021/ed039p333.

[gcb70125-bib-0073] Tang, J. , C. Körner , H. Muraoka , et al. 2016. “Emerging Opportunities and Challenges in Phenology: A Review.” Ecosphere 7: e01436. 10.1002/ecs2.1436.

[gcb70125-bib-0095] Vuichard, N. , P. Messina , S. Luyssaert , et al. 2019. “Accounting for Carbon and Nitrogen Interactions in the Global Terrestrial Ecosystem Model ORCHIDEE (trunk version, rev 4999): Multi‐scale Evaluation of Gross Primary Production.” Geoscientific Model Development 12, no. 11: 4751–4779. 10.5194/gmd-12-4751-2019.

[gcb70125-bib-0096] Walker, A. P. , T. Quaife , P. M. van Bodegom , et al. 2017. “The Impact of Alternative Trait‐Scaling Hypotheses for the Maximum Photosynthetic Carboxylation Rate (V_cmax_) on Global Gross Primary Production.” New Phytologist 215, no. 4: 1370–1386. 10.1111/nph.14623. 28643848

[gcb70125-bib-0074] Wang, H. , I. C. Prentice , T. F. Keenan , et al. 2017. “Towards a Universal Model for Carbon Dioxide Uptake by Plants.” Nature Plants 3: 734–741. 29150690 10.1038/s41477-017-0006-8

[gcb70125-bib-0075] Wang, S. , X. Lu , X. Cheng , X. Li , M. Peichl , and I. Mammarella . 2017. “Limitations and Challenges of MODIS‐Derived Phenological Metrics Across Different Landscapes in Pan‐Arctic Regions.” Remote Sensing 10, no. 11: 1784. 10.3390/rs10111784.

[gcb70125-bib-0108] Wiltshire, A. J. , E. J. Burke , S. E. Chadburn , et al. 2021. “JULES‐CN: A Coupled Terrestrial Carbon–Nitrogen Scheme (JULES vn5.1).” Geoscientific Model Development 14, no. 4: 2161–2186. 10.5194/gmd-14-2161-2021.

[gcb70125-bib-0077] Wu, J. , L. P. Albert , A. P. Lopes , et al. 2016. “Leaf Development and Demography Explain Photosynthetic Seasonality in Amazon Evergreen Forests.” Science 351: 972–976. 10.1126/science.aad5068. 26917771

[gcb70125-bib-0078] Xia, J. , S. Niu , P. Ciais , et al. 2015. “Joint Control of Terrestrial Gross Primary Productivity by Plant Phenology and Physiology.” Proceedings of the National Academy of Sciences of the United States of America 112: 2788–2793. 10.1073/pnas.1413090112. 25730847 PMC4352779

[gcb70125-bib-0079] Xia, J. , W. Yuan , Y.‐P. Wang , and Q. Zhang . 2017. “Adaptive Carbon Allocation by Plants Enhances the Terrestrial Carbon Sink.” Scientific Reports 7: 3341. 10.1038/s41598-017-03574-3. 28611453 PMC5469799

[gcb70125-bib-0092] Xie, Y. , D. L. Civco , and J. A. Silander . 2018. “Species‐Specific Spring and Autumn Leaf Phenology Captured by Time‐lapse Digital Cameras.” Ecosphere 9, no. 1: e02089. 10.1002/ecs2.2089.

[gcb70125-bib-0080] Xin, Q. 2016. “A Risk‐benefit Model to Simulate Vegetation Spring Onset in Response to Multi‐decadal Climate Variability: Theoretical Basis and Applications From the Field to the Northern Hemisphere.” Agricultural and Forest Meteorology 228–229: 139–163. 10.1016/j.agrformet.2016.06.017.

[gcb70125-bib-0081] Xin, Q. , Y. Dai , X. Li , X. Liu , P. Gong , and A. D. Richardson . 2018. “A Steady‐State Approximation Approach to Simulate Seasonal Leaf Dynamics of Deciduous Broadleaf Forests via Climate Variables.” Agricultural and Forest Meteorology 249: 44–56. 10.1016/j.agrformet.2017.11.025.

[gcb70125-bib-0082] Xin, Q. , Y. Dai , and X. Liu . 2019. “A Simple Time‐Stepping Scheme to Simulate Leaf Area Index, Phenology, and Gross Primary Production Across Deciduous Broadleaf Forests in the Eastern United States.” Biogeosciences 16: 467–484. 10.5194/bg-16-467-2019.

[gcb70125-bib-0083] Xin, Q. , X. Zhou , N. Wei , H. Yuan , Z. Ao , and Y. Dai . 2020. “A Semiprognostic Phenology Model for Simulating Multidecadal Dynamics of Global Vegetation Leaf Area Index.” Journal of Advances in Modeling Earth Systems 12: e2019MS001935. 10.1029/2019MS001935.

[gcb70125-bib-0084] Yu, J. , S. B. Kim , J. Bai , and S. W. Han . 2020. “Comparative Study on Exponentially Weighted Moving Average Approaches for the Self‐Starting Forecasting.” Applied Sciences 10: 7351. 10.3390/app10207351.

[gcb70125-bib-0098] Yuan, W. , D. Liu , W. Dong , et al. 2014. “Multiyear Precipitation Reduction Strongly Decreases Carbon Uptake Over Northern China.” Journal of Geophysical Research: Biogeosciences 119, no. 5: 881–896. 10.1002/2014jg002608.

[gcb70125-bib-0110] Zaehle, S. , and A. D. Friend . 2010. “Carbon and Nitrogen Cycle Dynamics in the O‐CN Land Surface Model: 1. Model Description, Site‐Scale Evaluation, and Sensitivity to Parameter Estimates.” Global Biogeochemical Cycles 24, no. 1. 10.1029/2009gb003521.

[gcb70125-bib-0087] Zhao, M. , C. Peng , W. Xiang , et al. 2013. “Plant Phenological Modeling and Its Application in Global Climate Change Research: Overview and Future Challenges.” Environmental Reviews 21, no. 1: 1–14. 10.1139/er-2012-0036.

[gcb70125-bib-0088] Zhou, G. , and Q. Wang . 2018. “A New Nonlinear Method for Calculating Growing Degree Days.” Scientific Reports 8: 10149. 10.1038/s41598-018-28392-z. 29977001 PMC6033920

[gcb70125-bib-0089] Zhou, X. , Q. Xin , S. Zhang , S. Delzon , and Y. Dai . 2023. “A Prognostic Vegetation Phenology Model to Predict Seasonal Maximum and Time Series of Global Leaf Area Index Using Climate Variables.” Agricultural and Forest Meteorology 342: 109739. 10.1016/j.agrformet.2023.109739.

[gcb70125-bib-0090] Zhu, Z. , H. Wang , S. P. Harrison , I. C. Prentice , S. Qiao , and S. Tan . 2023. “Optimality Principles Explaining Divergent Responses of Alpine Vegetation to Environmental Change.” Global Change Biology 29: 126–142. 10.1111/gcb.16459. 36176241 PMC10092415

