# Supplementary material for: A General Model for the Seasonal to Decadal Dynamics of Leaf Area
Source: Glob Chang Biol. 2025 Mar 21;31(3):e70125. doi: 10.1111/gcb.70125 (PMC11926779; doi:10.1111/gcb.70125)
Supplement: Supplementary file 1 — Data S1. [file GCB-31-e70125-s001.docx]

**A general model for the seasonal to decadal dynamics of leaf area: Supplementary Information**

Boya Zhou^1^, Wenjia Cai^1^, Ziqi Zhu^2^, Han Wang^2^, Sandy P. Harrison^3,2^, I. Colin Prentice^1,2^

^1^Georgina Mace Centre for the Living Planet, Department of Life Sciences, Imperial College London, Silwood Park Campus, Buckhurst Road, Ascot SL5 7PY, UK; ^2^Department of Earth System Science, Ministry of Education Key Laboratory for Earth System Modelling, Institute for Global Change Studies, Tsinghua University, Beijing, China; ^3^ School of Archaeology, Geography and Environmental Science (SAGES), University of Reading, Reading RG6 6AH, UK

This Supplementary Information contains the following items:

**Note 1:** Simulating the dynamics of steady-state leaf area index (Xin et al., 2020)

**Note 2:** Description of the exponentially weighted moving average method (Mengoli et al., 2022)

**Table S1.** Characteristics of the flux tower sites used in the model evaluation

**Table S2**. Summary statistics for the observed and modelled Leaf Area Index distributions

**Table S3**. Statistics for the MODIS-derived and modelled Leaf Area Index (LAI) across biomes on different timescales

**Table S4**. Statistics for the Copernicus-derived and modelled Leaf Area Index (LAI) across biomes on different timescales

**Table S5.** Summary coefficients for Fig. 5

**Table S6**. Fitted *σ* values using different sources

**Table S7**. Fitted *σ* values for different vegetation types

**Table S8**. Variation of σ values with environmental conditions

**Table S9.** Summary coefficients for Figure S12-S17, S21-S26.

**Figure S1**. RRMSE, R^2^ and bias error distributions of different vegetation types according to different lengths of acclimation time using the exponential moving average approach

**Figure S2** Mean seasonal cycle of LAI.

**Figure S3** Regression analysis is shown for satellite-derived (Copernicus) and simulated yearly average LAI for all vegetation types

**Figure S4** The spatial distribution of σ, inferred using different products.

**Figure S5** Correlation of observed and modelled annual mean LAI values for different model combinations

**Figure S6** The spatial distributions of multiyear average LAI (2000 to 2019) using different *σ* values.

**Figure S7** Mean seasonal cycle of potential GPP.

**Figure S8** Comparison of predicted and observed LAI_max._

**Figure S9** Differences between simulated and MODIS-derived multi-year LAI mean values (2001-2019)

**Figure S10** The seasonal differences between modelled LAI and MODIS-derived LAI values (2001-2019) on a global scale

**Figure S11** Regression analysis of multi-year average of simulated LAI and observed LAI from MODIS (2001 to 2019).

**Figure** **S12** The spatial distribution of relative root mean squared error (RRMSE) between modelled and MODIS-derived annual average LAI time series (from 2001 to 2019).

**Figure** **S13** The spatial distribution of bias between modelled and MODIS-derived annual average LAI time series (from 2001 to 2019).

**Figure** **S14** The spatial distribution of Pearson correlation coefficient (r) between modelled and MODIS-derived annual average LAI time series (from 2001 to 2019).

**Figure S15** The spatial distribution of relative root mean squared error (RRMSE) between modelled and MODIS-derived monthly LAI time series in 2010.

**Figure S16** The spatial distribution of bias between modelled and MODIS-derived monthly LAI time series in 2010.

**Figure S17** The spatial distribution of bias between modelled and MODIS-derived monthly LAI time series in 2010.

**Figure S18** Differences between simulated and Copernicus-derived multi-year LAI mean values (2001-2019)

**Figure S19** The seasonal differences between modelled LAI and Copernicus-derived LAI values (2001-2019) on a global scale

**Figure S20** Regression analysis of multi-year average of simulated LAI and observed LAI from Copernicus (2001 to 2019).

**Figure** **S21** The spatial distribution of relative root mean squared error (RRMSE) between modelled and Copernicus-derived annual average LAI time series (from 2001 to 2019).

**Figure** **S22** The spatial distribution of bias between modelled and Copernicus-derived annual average LAI time series (from 2001 to 2019).

**Figure** **S23** The spatial distribution of Pearson correlation coefficient (r) between modelled and Copernicus-derived annual average LAI time series (from 2001 to 2019).

**Figure S24** The spatial distribution of relative root mean squared error (RRMSE) between modelled and Copernicus-derived monthly LAI time series in 2010.

**Figure S25** The spatial distribution of bias between modelled and Copernicus-derived monthly LAI time series in 2010.

**Figure S26** The spatial distribution of bias between modelled and Copernicus-derived monthly LAI time series in 2010.

**Note 1: Simulating the dynamics of steady-state leaf area index (Xin et al., 2020)**

Recent theoretical developments indicate that there is a general, global relationship between leaf phenology and the seasonal time course of ‘steady-state LAI’: the LAI would be in equilibrium with GPP if weather conditions were held constant. This can be predicted from the time course of gross primary production (GPP) because LAI and GPP are mutually related, via the Beer’s law dependence of GPP on LAI, and the requirement for GPP to support LAI development. Given daily meteorological conditions, the steady-state LAI is modelled on a daily basis by solving the closed system of equations as follows (Xin et al., 2020):

$$L_{s} = m A_{s} (Eqn S1)$$

$A_{s} = A_{0} [1 - exp (-k L_{s})] (Eqn S2)$

where *L*_s_ denotes steady-state LAI; *m* is the ratio of steady-state LAI and GPP; *A*_s_ is the steady-state GPP; *A*_0_ is the potential GPP, which would be obtained if all incident light were absorbed by green tissues; and *k* = 0.5 denotes the canopy light extinction coefficient.

Equations S1 and S2 yield an analytical solution as follows. Let *μ* be an intermediate variable:

$$\mu= mA_{0} (Eqn S3)$$

Substituting *μ* into Equations S1 and S2:

$$L_{s} = \mu[1 - exp \left( -k L_{s} \right)] (Eqn S4)$$

Let *y* be another intermediate variable:

$$y = k \mu- k L_{s} (Eqn S5)$$

Substituting *y* into Equation S4:

$$y exp(-y) = k \mu exp (-k \mu) (Eqn S6)$$

Equation S6 can be solved:

$$y = W_{0}\{-k \mu exp (-k \mu)\} (Eqn S7)$$

where *W*_0_ denotes the principal branch of the Lambert W function. This function is defined as the inverse relation of the function *f(z)* = *ze^z^*. Note that *−k μ exp (− k μ)* has the range (−e^−1^,0] because *kμ* ≥ 0.

Substituting Equation S7 into Equation S5 yields the solution:

$$L_{s} = \mu+\frac{1}{k}W_{0}\left\{ -k \mu exp \left( -k \mu\right) \right\} (Eqn S8)$$

subject to the condition LAI_s_  ≤ LAI_max_, where LAI_max_ is the maximum LAI during the growing season.

**Note 2: Description of the exponentially weighted moving average method (Mengoli et al., 2022)**

The smoothing-in-time coefficient (α) defines the length of the recent period to be considered for acclimation. This parameter, ranging from 0 to 1, reflects the influence that past data (with an N-day memory) have on the current values of leaf traits, as shown below:

$$\mathrm{LAI}_{\mathrm{sim}}\left[ t \right]= \sum_{n=0}^{t} w\left[ t-n \right]\times L_{s}[t-n]; w\left[ t-n \right]= {\alpha(1 - \alpha)}^{n} (Eqn S9)$$

where *n* is the index of the time steps (days) back in time. The weight of *L*_s_ decreases exponentially as time progresses (so for example, the weight of 20 days ago is *w* (*t* – 20)/*w* (*t*) = (1 ­– α) ^20^ smaller than that of today). We can consider the *e*-folding time of the weights as the memory of the acclimation response. An *e*-folding decrease of the weights within a window of N days would imply a change in the weights of (1 – α) ^N^ = 1/*e*, hence N = – 1/ln (1 – α). For small α, ln (1 – α) ≈ – α (from the Taylor expansion) hence N ≈ 1/α.

**Table S1.** Characteristics of the flux tower sites used in the model evaluation, giving the unique code for each site (Site ID), latitude, longitude, elevation, climate classification (Climate), vegetation classification (using the IGBP classification scheme), sampling years (recording period) and reference. **Abbreviations**: **BSk**, Cold semi-arid (steppe) climate; **BSh**, Hot semi-arid (steppe) climate; **Cfa**, Humid subtropical climate; **Cfb**, Temperate oceanic climate; **Csb**, Warm-summer Mediterranean climate; **Dfa**, Hot-summer humid continental climate; **Dfb**, Warm-summer humid continental climate; **Dfc**, Subarctic climate; **CSH**, Closed shrubland; **DBF**, Deciduous broadleaf; **EBF**, Evergreen broadleaf; **ENF**, Evergreen needleaf; **GRA**, Grassland; **MF,** Mixed Forest; **OSH**, Open shrubland; **SAV**, Savanna; **WSA**, Woody savanna.

| **Site ID** | **Latitude**  **(°)** | **Longitude**  **(°)** | **Elevation (m)** | **Climate type** | **Vegetation**  **type** | **Recording period** | **Reference** |
| --- | --- | --- | --- | --- | --- | --- | --- |
| **Fluxnet2015** | | | | | | | |
| AR-SLu | -33.46 | -66.46 | 507 | BSk | MF | 2009-2011 | Ulke et al. (2015) |
| AR-Vir | -28.24 | -56.19 | 104 | Cfa | ENF | 2010-2012 | Posse et al. (2016) |
| AT-Neu | 47.12 | 11.32 | 970 | Dfb | GRA | 2002-2012 | Wohlfahrt et al.(2008) |
| AU-Ade | -13.08 | 131.12 | 79 | Aw | WSA | 2007-2009 | Beringer et al. (2011b) |
| AU-ASM | -22.28 | 133.25 | 605 | BSh | SAV | 2010-2014 | Cleverly et al. (2013) |
| AU-Cpr | -34.00 | 140.59 | 60 | BSk | SAV | 2010-2014 | Meyer et al. (2015) |
| AU-Cum | -33.61 | 150.72 | 351 | NA | EBF | 2012-2014 | Beringer et al.(2016) |
| AU-DaP | -14.06 | 131.32 | 69 | Aw | GRA | 2007-2013 | Beringer et al. (2011a) |
| AU-DaS | -14.16 | 131.39 | 79 | Aw | SAV | 2008-2014 | Hutley et al. (2011) |
| AU-Dry | -15.26 | 132.37 | 175 | Aw | SAV | 2008-2014 | Cernusak et al. (2011) |
| AU-Emr | -23.86 | 148.47 | 175 | BSh | GRA | 2011-2013 | Schroder et al. (2014) |
| AU-Gin | -31.38 | 115.71 | 50 | Csa | WSA | 2011-2014 | Beringer et al. (2016) |
| AU-How | -12.49 | 131.15 | 35 | Aw | WSA | 2001-2014 | Andrykanus et al. (2012) |
| AU-RDF | -14.56 | 132.48 | 181 | Aw | WSA | 2011-2013 | Bristow et al. (2016) |
| AU-Rig | -36.65 | 145.58 | 151 | Cfb | GRA | 2011-2014 | Beringer et al. (2016) |
| AU-Stp | -17.15 | 133.35 | 229 | BSh | GRA | 2009-2014 | Beringer et al. (2011a) |
| AU-TTE | -22.29 | 133.64 | 551 | BWh | GRA | 2013-2014 | Cleverly et al. (2016) |
| AU-Tum | -35.66 | 148.15 | 1238 | Cfb | EBF | 2001-2014 | Leuning et al. (2005) |
| AU-Wac | -37.43 | 145.19 | 732 | Cfb | EBF | 2005-2008 | Kilinc et al. (2013) |
| AU-Whr | -36.67 | 145.03 | 144 | Cfb | EBF | 2011-2014 | McHugh et al. (2017) |
| AU-Wom | -37.42 | 144.09 | 700 | Cfb | EBF | 2010-2014 | Hinko-Najera et al. (2017) |
| AU-Ync | -34.99 | 146.29 | 127 | BSk | GRA | 2012-2014 | Yee et al. (2015) |
| BE-Bra | 51.31 | 4.52 | 16 | Cfb | MF | 2001-2013 | Stoy et al. (2013) |
| BE-Vie | 50.30 | 6.00 | 486 | Cfb | MF | 2001-2014 | Aubinet et al. (2001) |
| BR-Sa1 | -2.85 | -54.95 | 88 | Am | EBF | 2002-2011 | Saleska et al.(2013) |
| BR-Sa3 | -3.02 | -54.97 | 172 | Am | EBF | 2001-2004 | Wick et al. (2005) |
| CA-Gro | 48.21 | -82.15 | 340 | Dfb | MF | 2003-2014 | NA |
| CA-Man | 55.88 | -98.48 | 261 | Dfc | ENF | 2001-2008 | Dunn et al. (2007) |
| CA-NS1 | 55.87 | -98.48 | 260 | Dfc | ENF | 2002-2005 | NA |
| CA-NS2 | 55.90 | -98.52 | 260 | Dfc | ENF | 2001-2005 | NA |
| CA-NS3 | 55.91 | -98.38 | 260 | Dfc | ENF | 2001-2005 | NA |
| CA-NS4 | 55.91 | -98.38 | 252 | Dfc | ENF | 2002-2005 | NA |
| CA-NS5 | 55.86 | -98.48 | 260 | Dfc | ENF | 2001-2005 | NA |
| CA-NS6 | 55.91 | -98.96 | 244 | Dfc | OSH | 2001-2005 | NA |
| CA-NS7 | 56.63 | -99.94 | 297 | Dfc | OSH | 2002-2005 | NA |
| CA-Oas | 53.62 | -106.19 | 530 | Dfc | DBF | 2001-2010 | NA |
| CA-Obs | 53.98 | -105.11 | 629 | Dfc | ENF | 2001-2010 | NA |
| CA-Qfo | 49.69 | -74.34 | 382 | Dfc | ENF | 2003-2010 | Bergeron et al.(2007) |
| CA-SF1 | 54.48 | -105.81 | 536 | Dfc | ENF | 2003-2006 | NA |
| CA-SF2 | 54.25 | -105.87 | 520 | Dfc | ENF | 2001-2005 | NA |
| CA-SF3 | 54.09 | -106.01 | 544 | Dfc | OSH | 2002-2006 | NA |
| CA-TP1 | 42.66 | -80.55 | 265 | Dfb | ENF | 2003-2014 | Arain et al.(2005) |
| CA-TP2 | 42.77 | -80.45 | 212 | Dfb | ENF | 2003-2007 | Arain et al.(2005) |
| CA-TP3 | 42.70 | -80.34 | 184 | Dfb | ENF | 2003-2014 | Arain et al.(2005) |
| CA-TP4 | 42.71 | -80.35 | 184 | Dfb | ENF | 2002-2014 | Arain et al.(2005) |
| CA-TPD | 42.63 | -80.55 | 260 | Dfb | DBF | 2012-2014 | Arain et al.(2005) |
| CG-Tch | -4.28 | 11.65 | 82 | NA | SAV | 2006-2009 | NA |
| CH-Cha | 47.21 | 8.41 | 393 | Cfb | GRA | 2005-2014 | Merbold et al.(2014) |
| CH-Dav | 46.81 | 9.85 | 1639 | ET | ENF | 2001-2014 | Zielis et al.(2014) |
| CH-Fru | 47.12 | 8.54 | 972 | Cfb | GRA | 2005-2014 | Imer et al. (2013) |
| CH-Lae | 47.47 | 8.36 | 689 | NA | MF | 2004-2014 | Etzold et al.(2011) |
| CH-Oe1 | 47.29 | 7.73 | 454 | Cfb | GRA | 2002-2008 | Ammann et al. (2009) |
| CN-Cha | 42.40 | 128.09 | NA | Dwb | MF | 2003-2005 | Guan et al.(2006) |
| CN-Cng | 44.59 | 123.50 | NA | NA | GRA | 2007-2010 | NA |
| CN-Din | 23.17 | 112.53 | NA | Cfa | EBF | 2003-2005 | Yan et al. (2013) |
| CN-Du2 | 42.05 | 116.28 | 1321 | Dwb | GRA | 2007-2008 | Chen et al. (2009) |
| CN-HaM | 37.37 | 101.18 | 4032 | ET | GRA | 2002-2004 | Kato et al. (2006) |
| CN-Qia | 26.74 | 115.05 | NA | Cfa | ENF | 2003-2005 | Wen et al. (2010) |
| CN-Sw2 | 41.79 | 111.89 | NA | NA | GRA | 2010-2012 | Shao et al.(2017) |
| CZ-BK1 | 49.50 | 18.53 | 875 | NA | ENF | 2004-2014 | Acosta et al.(2013) |
| CZ-BK2 | 49.49 | 18.54 | 855 | Dfb | GRA | 2006-2012 | NA |
| DE-Gri | 50.95 | 13.51 | 380 | Cfb | GRA | 2004-2014 | Prescher et al. (2010) |
| DE-Hai | 51.07 | 10.45 | 430 | NA | DBF | 2001-2012 | Knohl et al.(2003) |
| DE-Lkb | 49.09 | 13.30 | 1308 | NA | ENF | 2009-2013 | Lindauer et al.(2014) |
| DE-Lnf | 51.32 | 10.36 | 451 | Cfb | DBF | 2002-2012 | NA |
| DE-Obe | 50.78 | 13.72 | 734 | Cfb | ENF | 2008-2014 | NA |
| DE-RuR | 50.62 | 6.30 | 514 | Cfb | GRA | 2011-2014 | Post et al. (2015) |
| DE-Tha | 50.96 | 13.56 | 385 | Cfb | ENF | 2001-2014 | Grünwald and Bernhofer (2007) |
| DK-Eng | 55.69 | 12.19 | 10 | NA | GRA | 2005-2008 | NA |
| DK-Sor | 55.48 | 11.64 | 40 | NA | DBF | 2001-2014 | Pilegaard et al.(2011) |
| ES-Amo | 36.83 | -2.25 | 58 | NA | OSH | 2007-2012 | NA |
| ES-LgS | 37.10 | -2.97 | 2271 | Csa | OSH | 2007-2009 | Reverter et al. (2010) |
| ES-LJu | 36.92 | -2.75 | 1600 | NA | OSH | 2004-2013 | NA |
| FI-Let | 60.64 | 23.95 | 111 | NA | ENF | 2009-2012 | NA |
| FR-Fon | 48.48 | 2.78 | 103 | Cfb | DBF | 2005-2014 | Delpierre et al. (2015) |
| FR-LBr | 44.72 | -0.77 | 63 | Cfb | ENF | 2001-2008 | Berbigier et al. (2001) |
| FR-Pue | 43.74 | 3.60 | 269 | Csa | EBF | 2001-2014 | Rambal et al. (2004) |
| GF-Guy | 5.27 | -52.92 | 48 | NA | EBF | 2004-2014 | Bonal et al.(2008) |
| GH-Ank | 5.26 | -2.69 | 124 | NA | EBF | 2011-2014 | NA |
| IT-CA1 | 42.38 | 12.02 | 200 | NA | DBF | 2011-2014 | Sabbatini et al.(2016) |
| IT-CA3 | 42.38 | 12.02 | 197 | NA | DBF | 2011-2014 | Sabbatini et al.(2016) |
| IT-Col | 41.85 | 13.59 | 1549 | Cfa | DBF | 2001-2014 | Valentini et al. (1996) |
| IT-Cp2 | 41.70 | 12.36 | 3 | Csa | EBF | 2012-2014 | Fares et al. (2014) |
| IT-Cpz | 41.70 | 12.37 | 68 | NA | EBF | 2001-2008 | Wei et al.(2014) |
| IT-La2 | 45.95 | 11.28 | 1350 | NA | ENF | 2001-2002 | Marcolla et al.(2003) |
| IT-Lav | 45.95 | 11.28 | 1353 | NA | ENF | 2003-2014 | Marcolla et al.(2003) |
| IT-MBo | 46.01 | 11.05 | 1549 | Dfb | GRA | 2003-2013 | Marcolla et al. (2011) |
| IT-PT1 | 45.20 | 9.06 | 60 | NA | DBF | 2002-2004 | Migliavacca et al.(2009) |
| IT-Ren | 46.58 | 11.43 | 1730 | NA | ENF | 2002-2013 | Montagnani et al.(2009) |
| IT-Ro1 | 42.40 | 11.93 | 235 | NA | DBF | 2001-2008 | NA |
| IT-Ro2 | 42.39 | 11.92 | 160 | NA | DBF | 2002-2012 | Tedeschi et al.(2006) |
| IT-SRo | 43.73 | 10.28 | 6 | Csa | ENF | 2001-2012 | Chiesi et al. (2005) |
| IT-Tor | 45.84 | 7.58 | 2164 | Dfc | GRA | 2008-2014 | Galvagno et al. (2013) |
| JP-MBF | 44.38 | 142.31 | NA | NA | DBF | 2004-2005 | Matsumoto et al.(2008) |
| JP-SMF | 35.26 | 137.07 | NA | NA | MF | 2002-2006 | Matsumoto et al.(2008) |
| MY-PSO | 2.97 | 102.30 | NA | NA | EBF | 2003-2009 | NA |
| NL-Hor | 52.24 | 5.07 | 2.2 | Cfb | GRA | 2004-2011 | Jacobs et al. (2007) |
| NL-Loo | 52.16 | 5.74 | 25 | NA | ENF | 2001-2014 | Moors et al.(2012) |
| PA-SPn | 9.31 | -79.63 | 78 | NA | DBF | 2007-2009 | NA |
| PA-SPs | 9.31 | -79.63 | 68 | NA | GRA | 2007-2009 | NA |
| RU-Fyo | 56.46 | 32.92 | 265 | Dfb | ENF | 2001-2014 | Kurbatova et al. (2008) |
| RU-Ha1 | 54.73 | 90.00 | 446 | Dfc | GRA | 2002-2004 | Belelli Marchesini et al. (2007) |
| SD-Dem | 13.28 | 30.47 | 500 | NA | SAV | 2005-2009 | Ardö et al.(2008) |
| SN-Dhr | 15.40 | -15.43 | 40 | NA | SAV | 2010-2013 | Tagesson et al.(2014) |
| US-AR1 | 36.43 | -99.42 | 612 | Cfa | GRA | 2009-2012 | NA |
| US-AR2 | 36.64 | -99.60 | 645 | BSk | GRA | 2009-2012 | NA |
| US-Blo | 38.89 | -120.63 | 1315 | Csa | ENF | 2001-2007 | Reichstein et al.(2003) |
| US-Cop | 38.09 | -109.39 | 1520 | BSk | GRA | 2001-2007 | Bowling et al. (2010) |
| US-GBT | 41.36 | -106.23 | 3191 | Dfc | ENF | 2001-2003 | Zeller and Nikolov (2000) |
| US-GLE | 41.36 | -106.23 | 3197 | Dfc | ENF | 2005-2014 | Frank et al. (2014) |
| US-Goo | 34.25 | -89.87 | 87 | Cfa | GRA | 2002-2006 | NA |
| US-Ha1 | 42.53 | -72.17 | 340 | Dfb | DBF | 2001-2012 | Urbanski et al. (2007a) |
| US-IB2 | 41.84 | -88.24 | 226.5 | Dfa | GRA | 2004-2011 | Allison et al.(2005) |
| US-KS2 | 28.61 | -80.67 | 2 | Cfa | CSH | 2003-2006 | Powell et al. (2006) |
| US-Me2 | 44.45 | -121.55 | 1253 | Csb | ENF | 2002-2014 | Irvine et al. (2008) |
| US-Me3 | 44.31 | -121.60 | 1005 | Csb | ENF | 2004-2009 | Irvine et al. (2008) |
| US-Me5 | 44.43 | -121.56 | 1188 | Csb | ENF | 2001-2002 | Irvine et al. (2008) |
| US-Me6 | 44.32 | -121.60 | 998 | Csb | ENF | 2010-2014 | Ruehr et al. (2012) |
| US-MMS | 39.32 | -86.41 | 275 | Cfa | DBF | 2001-2014 | Dragoni et al. (2011) |
| US-NR1 | 40.03 | -105.54 | 3050 | Dfc | ENF | 2001-2014 | Monson et al. (2002) |
| US-Oho | 41.55 | -83.84 | 230 | Dfa | DBF | 2004-2013 | NA |
| US-PFa | 45.95 | -90.27 | 471 | Dfb | MF | 2001-2014 | Desai et al. (2015) |
| US-SRC | 31.90 | -110.83 | 950 | Bwh | OSH | 2008-2013 | NA |
| US-SRG | 31.79 | -110.83 | 1293 | BSk | GRA | 2008-2014 | Scott et al. (2015a) |
| US-SRM | 31.82 | -110.87 | 1113 | BSk | WSA | 2004-2014 | Scott et al. (2009) |
| US-Sta | 41.39 | -106.80 | 2069 | Bsh | OSH | 2005-2009 | NA |
| US-Syv | 46.24 | -89.35 | 544 | Dfb | MF | 2001-2014 | Desai et al. (2005) |
| US-Ton | 38.43 | -120.97 | 174 | Csa | WSA | 2001-2014 | Baldocchi et al. (2010) |
| US-UMB | 45.55 | -84.71 | 234 | Dfb | DBF | 2001-2014 | Gough et al. (2013) |
| US-UMd | 45.56 | -84.69 | 239 | Dfb | DBF | 2008-2014 | Gough et al. (2013) |
| US-Var | 38.41 | -120.95 | 166 | Csa | GRA | 2001-2014 | Ma et al. (2007) |
| US-WCr | 45.80 | -90.07 | 520 | Dfb | DBF | 2001-2014 | Cook et al. (2004) |
| US-Whs | 31.74 | -110.05 | 1372 | BSk | OSH | 2007-2014 | Scott et al. (2015a) |
| US-Wi3 | 46.63 | -91.09 | 411 | Dfb | DBF | 2002-2004 | Noormets et al. (2007) |
| US-Wi4 | 46.73 | -91.16 | 352 | Dfb | ENF | 2002-2005 | Noormets et al. (2007) |
| US-Wkg | 31.74 | -109.94 | 1515 | BSk | GRA | 2004-2014 | Scott et al. (2010) |
| ZA-Kru | -25.02 | 31.50 | 357 | Cwa | SAV | 2001-2013 | Archibald et al. (2009) |
| ZM-Mon | -15.44 | 23.25 | 1087 | Aw | DBF | 2007-2009 | Merbold et al. (2009) |
| **ONEFLUX** | | | | | | | |
| CA-Ca1 | 49.86 | -125.33 | 300 | Cfb | ENF | 2001-2010 | Black et al.(2018) |
| CA-Ca2 | 49.87 | -125.29 | 300 | Cfb | ENF | 2001-2010 | Black et al.(2018) |
| CA-Ca3 | 49.53 | -124.90 | NA | Cfb | ENF | 2001-2010 | Black et al.(2023) |
| CA-Cbo | 44.31 | -79.93 | 120 | Dfb | DBF | 2001-2014 | Ralf et al.(2022) |
| CA-Qc2 | 49.75 | -74.57 | NA | NA | MF | 2008-2010 | Hank et al.(2018) |
| CA-SF2 | 54.25 | -105.87 | 520 | Dfc | ENF | 2003-2005 | NA |
| CA-SJ2 | 53.94 | -104.64 | 580 | Dfc | ENF | 2003-2007 | Alan et al.(2018) |
| CA-TP4 | 42.71 | -80.35 | 184 | Dfb | ENF | 2003-2017 | NA |
| US-Bar | 44.06 | -71.28 | 272 | Dfb | DBF | 2005-2017 | Richardson et al.(2023) |
| US-Dk1 | 35.97 | -79.09 | 168 | Cfa | GRA | 2001-2008 | Chris et al.(2018) |
| US-Dk2 | 35.97 | -79.10 | 168 | Cfa | DBF | 2001-2008 | Chris et al.(2018) |
| US-Dk3 | 35.97 | -79.09 | 163 | Cfa | ENF | 2001-2008 | Chris et al.(2018) |
| US-Fmf | 35.14 | -111.72 | 2160 | Csb | ENF | 2005-2010 | Sabina et al.(2019) |
| US-FR2 | 29.94 | -97.99 | 271.9 | Cfa | WSA | 2005-2008 | Marcy (2016) |
| US-Fuf | 35.08 | -111.76 | 2180 | Csb | ENF | 2005-2010 | Sabina et al.(2019) |
| US-Ho1 | 45.20 | -68.74 | 60 | Dfb | ENF | 2012-2018 | David (2021) |
| US-KFS | 39.05 | -95.19 | 310 | Cfa | GRA | 2007-2017 | Nathaniel (2020) |
| US-Mpj | 34.43 | -106.23 | 2196 | Bsk | WSA | 2008-2017 | Marcy (2023) |
| US-Rls | 43.14 | -116.73 | 1608 | Bsh | CSH | 2014-2017 | Gerald (2023) |
| US-Rms | 43.06 | -116.74 | 2111 | Bsh | CSH | 2014-2017 | Gerald (2022) |
| US-Ro4 | 44.67 | -93.07 | 274 | Dfa | GRA | 2014-2017 | John (2022) |
| US-Rws | 43.16 | -116.71 | 1425 | Bsk | OSH | 2014-2017 | Gerald (2023) |
| US-Seg | 34.36 | -106.70 | 1596 | Bsk | GRA | 2007-2017 | Marcy (2023) |
| US-Ses | 34.33 | -106.74 | 1604 | Bsk | OSH | 2007-2017 | Marcy (2023) |
| US-SRG | 31.78 | -110.82 | 1291 | Bsk | GRA | 2008-2017 | Russell (2023) |
| US-Vcm | 35.88 | -106.53 | 3030 | Dfb | ENF | 2007-2017 | Marcy (2023) |
| US-Vcp | 35.86 | -106.59 | 2500 | Dfb | ENF | 2007-2017 | Marcy (2023) |
| US-Wjs | 34.42 | -105.86 | 1931 | Bsk | SAV | 2008-2017 | Marcy (2023) |

**Table S2**. Summary statistics for the MODIS-derived and modelled Leaf Area Index (LAI) distributions according to different lengths of acclimation time (n. of days) using the exponential moving average approach. The statistics (RRMSE, R^2^, and bias) of different vegetation types were calculated in three steps: (1) Calculate RRMSE, R^2^, and bias for daily LAI of every week during growing season at each site. (2) Calculate the median values of RRMSE, R^2^, and bias among all weeks for each site to remove extreme values. (3) Calculate the mean values of RRMSE, R^2^, and bias among sites for each vegetation type. **Abbreviations**: **CSH**, Closed shrubland; **DBF**, Deciduous broadleaf; **EBF**, Evergreen broadleaf; **ENF**, Evergreen needleaf; **GRA**, Grassland; **MF,** Mixed Forest; **OSH**, Open shrubland; **SAV**, Savanna; **WSA**, Woody savanna.

| Vegetation type | α = 0.33 (3 days) | | | α = 0.143 (7 days) | | | α = 0.1 (10 days) | | | α = 0.067 (15 days) | | | α = 0.05 (20 days) | | |
| --- | --- | --- | --- | --- | --- | --- | --- | --- | --- | --- | --- | --- | --- | --- | --- |
|  | RRMSE | R^2^ | bias | RRMSE | R^2^ | bias | RRMSE | R^2^ | bias | RRMSE | R^2^ | bias | RRMSE | R^2^ | bias |
| ENF | 27.82 | 0.36 | 0.27 | 26.78 | 0.40 | 0.20 | 25.72 | 0.43 | 0.23 | 24.76 | 0.55 | 0.17 | 24.83 | 0.55 | 0.13 |
| EBF | 25.63 | 0.25 | 0.56 | 23.93 | 0.28 | 0.65 | 23.76 | 0.26 | 0.57 | 22.10 | 0.27 | 0.48 | 22.17 | 0.28 | 0.47 |
| DBF | 31.12 | 0.41 | -0.20 | 30.12 | 0.44 | -0.20 | 29.88 | 0.46 | -0.19 | 28.13 | 0.48 | -0.13 | 28.10 | 0.47 | -0.09 |
| MF | 30.12 | 0.48 | 0.50 | 29.45 | 0.51 | 0.49 | 28.85 | 0.53 | 0.48 | 27.92 | 0.54 | 0.41 | 27.12 | 0.54 | 0.38 |
| OSH | 56.78 | 0.20 | -0.09 | 54.32 | 0.21 | -0.08 | 51.46 | 0.21 | -0.09 | 50.10 | 0.20 | -0.05 | 50.00 | 0.21 | -0.04 |
| CSH | 32.67 | 0.31 | 0.09 | 30.45 | 0.34 | 0.08 | 29.87 | 0.33 | 0.08 | 27.29 | 0.36 | -0.02 | 28.12 | 0.36 | 0.04 |
| WSA | 40.32 | 0.43 | 0.54 | 37.68 | 0.46 | 0.44 | 35.46 | 0.45 | 0.42 | 33.13 | 0.46 | 0.32 | 31.23 | 0.50 | 0.29 |
| SAV | 43.54 | 0.07 | 0.04 | 36.50 | 0.04 | 0.03 | 38.22 | 0.03 | 0.03 | 36.21 | 0.04 | 0.08 | 33.63 | 0.04 | 0.01 |
| GRA | 34.27 | 0.51 | 0.14 | 29.74 | 0.56 | 0.20 | 30.99 | 0.51 | 0.16 | 26.33 | 0.63 | 0.15 | 27.06 | 0.62 | 0.12 |
|  | α = 0.04 (25 days) | | | α = 0.033 (30 days) | | | α = 0.022 (45 days) | | | α = 0.0167 (60 days) | | |  | | |
| ENF | 24.65 | 0.56 | 0.14 | 24.21 | 0.57 | 0.13 | 23.59 | 0.58 | 0.12 | 22.99 | 0.59 | 0.14 |  |  |  |
| EBF | 21.97 | 0.30 | 0.46 | 21.88 | 0.31 | 0.44 | 20.95 | 0.31 | 0.46 | 20.12 | 0.33 | 0.43 |  |  |  |
| DBF | 27.67 | 0.48 | -0.08 | 26.54 | 0.50 | -0.08 | 26.34 | 0.49 | -0.07 | 26.12 | 0.50 | 0.03 |  |  |  |
| MF | 26.19 | 0.56 | 0.38 | 27.12 | 0.56 | 0.38 | 26.38 | 0.57 | 0.36 | 26.32 | 0.58 | 0.36 |  |  |  |
| OSH | 49.86 | 0.21 | -0.04 | 49.67 | 0.22 | -0.04 | 48.76 | 0.28 | -0.04 | 46.56 | 0.29 | -0.05 |  |  |  |
| CSH | 27.47 | 0.38 | 0.06 | 27.12 | 0.36 | 0.12 | 26.79 | 0.38 | 0.13 | 26.12 | 0.39 | 0.10 |  |  |  |
| WSA | 32.12 | 0.51 | 0.29 | 32.16 | 0.55 | 0.28 | 31.10 | 0.55 | 0.28 | 30.98 | 0.56 | 0.30 |  |  |  |
| SAV | 31.61 | 0.07 | 0.02 | 30.88 | 0.05 | 0.02 | 30.25 | 0.05 | 0.01 | 28.15 | 0.06 | 0.01 |  |  |  |
| GRA | 26.15 | 0.63 | 0.11 | 25.57 | 0.65 | 0.12 | 24.11 | 0.64 | 0.11 | 25.62 | 0.63 | 0.09 |  |  |  |

**Table S3**. Statistics for the MODIS-derived and modelled Leaf Area Index (LAI) across biomes on different timescales **(a)** Model_Flux_: Simulate LAI dynamics using Flux tower GPP and MODIS derived seasonal maximum LAI (LAI_max_) as inputs; (**b)** Model_Prognostic_: Simulate LAI dynamics using P model-derived GPP and simulated LAI from fAPAR_max_ model as inputs. Reported metrics are the Pearson correlation coefficient (r), relative root means square error (RRMSE), R^2^ and bias.

|  | 1. **Model_Flux_** | | | | 1. **Model_Prognostic_** | | | |  |
| --- | --- | --- | --- | --- | --- | --- | --- | --- | --- |
| Timesteps | r | RRMSE | R^2^ | bias | r | RRMSE | R^2^ | bias | N |
| **Evergreen Needleaf** | | | | | | | | | |
| 7 days | 0.568 | 30.05 | 0.389 | 0.207 | 0.724 | 24.76 | 0.548 | 0.17 | 18489 |
| Seasonal | 0.649 | 27.85 | 0.483 | 0.190 | 0.738 | 23.18 | 0.575 | 0.158 | 4510 |
| Annual | 0.853 | 12.71 | 0.715 | 0.217 | 0.615 | 15.40 | 0.379 | 0.182 | 398 |
| Spatial | 0.859 | 12.08 | 0.775 | 0.293 | 0.628 | 15.83 | 0.481 | 0.288 | 53 |
| **Evergreen Broadleaf** | | | | | | | | | |
| 7 days | 0.750 | 16.37 | 0.562 | 0.238 | 0.621 | 22.10 | 0.270 | 0.483 | 3584 |
| Seasonal | 0.788 | 16.08 | 0.633 | 0.227 | 0.555 | 21.31 | 0.290 | 0.370 | 862 |
| Annual | 0.939 | 8.83 | 0.914 | 0.242 | 0.825 | 31.44 | 0.439 | 0.368 | 72 |
| Spatial | 0.945 | 5.16 | 0.950 | 0.191 | 0.734 | 16.53 | 0.463 | 0.166 | 12 |
| **Deciduous Broadleaf** | | | | | | | | | |
| 7 days | 0.775 | 22.87 | 0.793 | -0.130 | 0.778 | 28.13 | 0.482 | -0.127 | 8434 |
| Seasonal | 0.788 | 16.06 | 0.633 | 0.227 | 0.805 | 27.45 | 0.590 | 0.224 | 2562 |
| Annual | 0.817 | 8.01 | 0.628 | -0.130 | 0.598 | 13.14 | 0.235 | 0.262 | 219 |
| Spatial | 0.926 | 6.91 | 0.812 | -0.114 | 0.518 | 13.18 | 0.290 | 0.135 | 26 |
| **Mixed Forest** | | | | | | | | | |
| 7 days | 0.695 | 18.84 | 0.579 | 0.125 | 0.786 | 27.92 | 0.537 | 0.405 | 3855 |
| Seasonal | 0.759 | 21.35 | 0.681 | 0.100 | 0.805 | 27.51 | 0.568 | 0.038 | 932 |
| Annual | 0.695 | 6.50 | 0.718 | 0.134 | 0.231 | 15.58 | 0.129 | -0.001 | 79 |
| Spatial | 0.916 | 5.94 | 0.937 | 0.140 | 0.133 | 16.83 | 0.226 | 0.251 | 9 |
| **Open Shrubland** | | | | | | | | | |
| 7 days | 0.586 | 30.87 | 0.512 | 0.077 | 0.582 | 50.1 | 0.205 | -0.047 | 2448 |
| Seasonal | 0.664 | 30.40 | 0.620 | 0.078 | 0.578 | 45.1 | 0.197 | -0.006 | 618 |
| Annual | 0.938 | 17.3 | 0.831 | 0.102 | 0.594 | 41.45 | 0.240 | -0.006 | 55 |
| Spatial | 0.918 | 16.32 | 0.829 | 0.094 | 0.417 | 32.50 | 0.370 | -0.008 | 11 |
| **Closed Shrubland** | | | | | | | | | |
| 7 days | 0.830 | 15.24 | 0.741 | 0.148 | 0.661 | 27.29 | 0.359 | -0.024 | 950 |
| Seasonal | 0.664 | 30.89 | 0.620 | 0.09 | 0.684 | 27.66 | 0.375 | -0.033 | 618 |
| Annual | 0.954 | 7.39 | 0.984 | 0.144 | 0.878 | 18.07 | 0.702 | -0.017 | 19 |
| Spatial | 1 | 7.13 | 0.996 | 0.11 | 1 | 21.0 | 0.618 | -0.001 | 4 |
| **Woody Savannas** | | | | | | | | | |
| 7 days | 0.689 | 26.01 | 0.443 | 0.062 | 0.710 | 33.13 | 0.457 | 0.319 | 2771 |
| Seasonal | 0.812 | 25.88 | 0.527 | 0.142 | 0.723 | 31.78 | 0.358 | 0.275 | 672 |
| Annual | 0.817 | 13.44 | 0.807 | 0.156 | 0.713 | 26.59 | 0.564 | 0.320 | 56 |
| Spatial | 0.833 | 14.77 | 0.795 | 0.139 | 0.714 | 27.52 | 0.540 | 0.311 | 8 |
| **Savanna** | | | | | | | | | |
| 7 days | 0.522 | 27.36 | 0.375 | 0.152 | 0.300 | 36.21 | 0.038 | 0.080 | 1934 |
| Seasonal | 0.732 | 24.87 | 0.482 | 0.165 | 0.309 | 31.54 | 0.231 | 0.275 | 472 |
| Annual | 0.697 | 17.59 | 0.610 | 0.183 | 0.104 | 32.14 | 0.026 | 0.269 | 37 |
| Spatial | 0.785 | 14.51 | 0.838 | 0.179 | 0.05 | 36.31 | 0.003 | 0.09 | 7 |
| **Grassland** | | | | | | | | | |
| 7 days | 0.722 | 27.3 | 0.688 | 0.041 | 0.770 | 26.33 | 0.634 | 0.147 | 9300 |
| Seasonal | 0.799 | 21.51 | 0.679 | 0.030 | 0.794 | 26.24 | 0.656 | 0.129 | 2328 |
| Annual | 0.911 | 10.16 | 0.848 | 0.041 | 0.807 | 16.83 | 0.692 | 0.147 | 200 |
| Spatial | 0.934 | 8.29 | 0.878 | 0.018 | 0.846 | 17.51 | 0.691 | 0.110 | 33 |
| **Overall** | | | | | | | | | |
| 7 days | 0.782 | 23.49 | 0.67 | 0.100 | 0.746 | 27.41 | 0.60 | 0.155 | 53863 |
| Seasonal | 0.758 | 25.00 | 0.70 | 0.087 | 0.757 | 26.04 | 0.67 | 0.014 | 13184 |
| Annual | 0.738 | 17.24 | 0.89 | 0.221 | 0.783 | 17.67 | 0.70 | -0.092 | 1138 |
| Spatial | 0.931 | 10.73 | 0.90 | 0.131 | 0.769 | 16.02 | 0.72 | 0.197 | 163 |

**Table S4**. Statistics for the Copernicus-derived and modelled Leaf Area Index (LAI) across biomes on different timescales **(a)** Model_Flux_: Simulate LAI dynamics using Flux tower GPP and Copernicus derived seasonal maximum LAI (LAI_max_) as inputs; (**b)** Model_Prognostic_: Simulate LAI dynamics using P model-derived GPP and simulated LAI from fAPAR_max_ model as inputs. Reported metrics are the Pearson correlation coefficient (r), relative root means square error (RRMSE), R^2^ and bias.

|  | 1. **Model_Flux_** | | | | 1. **Model_Prognostic_** | | | |  |
| --- | --- | --- | --- | --- | --- | --- | --- | --- | --- |
| Timesteps | r | RRMSE | R^2^ | bias | r | RRMSE | R^2^ | bias | N |
| **Evergreen Needleaf** | | | | | | | | | |
| 7 days | 0.670 | 28.12 | 0.469 | -0.059 | 0.634 | 25.42 | 0.386 | 0.109 | 12668 |
| Seasonal | 0.721 | 25.23 | 0.558 | -0.036 | 0.659 | 25.22 | 0.414 | 0.155 | 2543 |
| Annual | 0.820 | 13.68 | 0.534 | -0.042 | 0.195 | 17.19 | 0.045 | 0.166 | 209 |
| Spatial | 0.868 | 12.45 | 0.635 | 0.049 | 0.417 | 18.87 | 0.134 | 0.167 | 29 |
| **Evergreen Broadleaf** | | | | | | | | | |
| 7 days | 0.844 | 13.02 | 0.712 | 0.094 | 0.608 | 20.10 | 0.464 | 0.422 | 5182 |
| Seasonal | 0.869 | 11.82 | 0.765 | 0.074 | 0.489 | 19.71 | 0.483 | 0.425 | 862 |
| Annual | 0.914 | 8.60 | 0.877 | 0.095 | 0.717 | 17.43 | 0.629 | 0.407 | 90 |
| Spatial | 0.898 | 6.81 | 0.930 | 0.076 | 0.608 | 19.26 | 0.444 | 0.351 | 14 |
| **Deciduous Broadleaf** | | | | | | | | | |
| 7 days | 0.815 | 24.44 | 0.764 | -0.260 | 0.716 | 32.71 | 0.534 | 0.273 | 10534 |
| Seasonal | 0.850 | 21.49 | 0.823 | -0.250 | 0.740 | 31.86 | 0.568 | 0.264 | 2112 |
| Annual | 0.794 | 9.29 | 0.692 | -0.246 | 0.580 | 15.09 | 0.487 | 0.281 | 175 |
| Spatial | 0.892 | 9.30 | 0.740 | -0.206 | 0.500 | 13.99 | 0.545 | 0.235 | 22 |
| **Mixed Forest** | | | | | | | | | |
| 7 days | 0.767 | 24.12 | 0.619 | -0.225 | 0.737 | 25.36 | 0.556 | 0.292 | 3167 |
| Seasonal | 0.835 | 18.49 | 0.752 | -0.217 | 0.749 | 25.72 | 0.609 | 0.266 | 636 |
| Annual | 0.853 | 7.41 | 0.833 | -0.218 | 0.329 | 16.27 | 0.130 | 0.289 | 53 |
| Spatial | 0.750 | 9.64 | 0.842 | -0.280 | 0.285 | 15.89 | 0.325 | 0.146 | 7 |
| **Open Shrubland** | | | | | | | | | |
| 7 days | 0.496 | 34.13 | 0.467 | 0.083 | 0.351 | 52.92 | 0.116 | -0.018 | 2270 |
| Seasonal | 0.593 | 30.29 | 0.540 | 0.083 | 0.375 | 57.06 | 0.125 | -0.006 | 456 |
| Annual | 0.791 | 18.21 | 0.852 | 0.084 | 0.449 | 48.65 | 0.182 | -0.023 | 38 |
| Spatial | 0.885 | 18.34 | 0.982 | 0.103 | 0.085 | 42.80 | 0.224 | -0.024 | 6 |
| **Closed Shrubland** | | | | | | | | | |
| 7 days | 0.671 | 20.71 | 0.441 | 0.203 | 0.432 | 21.79 | 0.152 | 0.318 | 239 |
| Seasonal | 0.762 | 18.11 | 0.604 | 0.201 | 0.449 | 21.58 | 0.168 | 0.317 | 48 |
| Annual | 0.800 | 15.48 | 0.689 | 0.298 | -0.8 | 15.67 | 0.297 | 0.311 | 4 |
| Spatial | NA | NA | NA | NA | NA | NA | NA | NA | 1 |
| **Woody Savannas** | | | | | | | | | |
| 7 days | 0.711 | 40.55 | 0.498 | -0.287 | 0.588 | 50.46 | 0.419 | -0.353 | 2826 |
| Seasonal | 0.765 | 37.59 | 0.557 | -0.285 | 0.612 | 55.87 | 0.448 | -0.350 | 564 |
| Annual | 0.907 | 25.88 | 0.871 | -0.283 | 0.822 | 47.77 | 0.486 | -0.346 | 49 |
| Spatial | 0.600 | 19.70 | 0.870 | -0.173 | 0.485 | 62.66 | 0.413 | -0.414 | 8 |
| **Savanna** | | | | | | | | | |
| 7 days | 0.539 | 38.09 | 0.277 | -0.202 | 0.322 | 46.11 | 0.155 | -0.208 | 2109 |
| Seasonal | 0.765 | 36.59 | 0.557 | -0.285 | 0.328 | 45.25 | 0.162 | -0.209 | 564 |
| Annual | 0.642 | 21.78 | 0.627 | -0.199 | 0.125 | 43.50 | 0.077 | -0.218 | 36 |
| Spatial | 0.857 | 14.95 | 0.967 | -0.167 | 0.071 | 46.65 | 0.054 | -0.387 | 7 |
| **Grassland** | | | | | | | | | |
| 7 days | 0.771 | 30.14 | 0.665 | -0.037 | 0.707 | 32.05 | 0.517 | 0.060 | 9716 |
| Seasonal | 0.850 | 24.62 | 0.756 | -0.038 | 0.714 | 31.06 | 0.533 | 0.059 | 1943 |
| Annual | 0.956 | 9.27 | 0.925 | -0.023 | 0.604 | 23.90 | 0.448 | 0.077 | 163 |
| Spatial | 0.967 | 10.59 | 0.921 | -0.048 | 0.561 | 32.37 | 0.247 | 0.262 | 27 |
| **Overall** | | | | | | | | | |
| 7 days | 0.796 | 25.69 | 0.710 | -0.102 | 0.679 | 30.32 | 0.486 | 0.091 | 48720 |
| Seasonal | 0.843 | 22.06 | 0.777 | -0.095 | 0.693 | 25.39 | 0.508 | 0.096 | 9754 |
| Annual | 0.907 | 11.04 | 0.871 | -0.090 | 0.507 | 21.26 | 0.635 | 0.091 | 1138 |
| Spatial | 0.939 | 10.46 | 0.904 | -0.052 | 0.523 | 24.44 | 0.449 | 0.047 | 119 |

**Table S5.** Summary coefficients for Fig. 5. **Annotations:** CSH, Closed shrubland; DBF, Deciduous broadleaf; EBF, Evergreen broadleaf; ENF, Evergreen needleaf; GRA, Grassland; MF, Mixed Forest; OSH, Open shrubland; SAV, Savanna; WSA, Woody savanna; PFT, Plant Functional Type. Each point represents one site-year of data.

| **PFT** | **LAI_sim_ (Model_Flux_)** | | | **LAI_sim_ (Model_Prognostic_)** | | |
| --- | --- | --- | --- | --- | --- | --- |
|  | Slope | R^2^ | RRMSE | Slope | R^2^ | RRMSE |
| CSH | 1.141 | 0.98 | 7.39 | 1.621 | 0.70 | 18.07 |
| DBF | 0.884 | 0.63 | 8.01 | 0.530 | 0.24 | 13.14 |
| EBF | 0.884 | 0.91 | 8.83 | 0.795 | 0.44 | 31.44 |
| ENF | 0.789 | 0.72 | 12.71 | 0.725 | 0.38 | 15.40 |
| GRA | 0.964 | 0.85 | 10.16 | 0.814 | 0.69 | 16.83 |
| MF | 0.705 | 0.72 | 6.50 | 0.031 | 0.13 | 15.58 |
| OSH | 1.134 | 0.83 | 17.30 | 0.265 | 0.24 | 41.45 |
| SAV | 0.909 | 0.61 | 17.59 | 0.157 | 0.03 | 32.14 |
| WSA | 1.036 | 0.88 | 13.44 | 0.562 | 0.56 | 26.59 |
| Overall | 0.904 | 0.89 | 17.24 | 0.934 | 0.72 | 16.02 |

**Table S6**. Fitted *σ* values using: (a) Flux tower-derived Gross Primary Production (GPP) data and MODIS derived Fraction of absorbed photosynthetically active radiation (fAPAR) and Leaf Area Index (LAI) data; (b) Flux-tower-derived GPP data and Copernicus derived fAPAR and LAI data; (c) P model-derived GPP data and MODIS fAPAR and LAI products, (d) P model-derived GPP data and AVHRR fAPAR and LAI products, and (e) P model-derived GPP data and GLOBMAP fAPAR and LAI products.

| **Input data combinations** | **Fitted *σ* values** |
| --- | --- |
| Site level | |
| 1. Flux-GPP + MODIS fAPAR/LAI | 0.771 |
| 1. Flux-GPP + Copernicus fAPAR/LAI | 0.711 |
| Global level | |
| 1. P model-GPP + MODIS fAPAR/LAI | 0.800 |
| 1. P model-GPP + AVHRR fAPAR/LAI | 0.792 |
| 1. P model-GPP + GLOBMAP fAPAR/LAI | 0.792 |

**Table S7**. Fitted *σ* values for different vegetation types based on MODIS satellite and Copernicus satellite data at Flux sites. RRMSE and R^2^ are the summary coefficients between observed and predicted m values for each corresponding σ value. **Abbreviations**: **CSH**, Closed shrubland; **DBF**, Deciduous broadleaf; **EBF**, Evergreen broadleaf; **ENF**, Evergreen needleaf; **GRA**, Grassland; **MF,** Mixed Forest; **OSH**, Open shrubland; **SAV**, Savanna; **WSA**, Woody savanna.

| **Vegetation Type** | **MODIS** | | | **Copernicus** | | |
| --- | --- | --- | --- | --- | --- | --- |
|  | *σ* | RRMSE | R^2^ | *σ* | RRMSE | R^2^ |
| MF | 0.633 | 50.55 | 0.92 | 0.670 | 48.49 | 0.89 |
| GRA | 0.816 | 50.48 | 0.84 | 0.736 | 55.39 | 0.96 |
| ENF | 0.824 | 37.44 | 0.88 | 0.815 | 40.17 | 0.73 |
| DBF | 0.667 | 59.94 | 0.89 | 0.632 | 62.64 | 0.75 |
| SAV | 0.737 | 48.86 | 0.87 | 0.723 | 46.84 | 0.85 |
| EBF | 0.958 | 15.21 | 0.96 | 0.907 | 17.52 | 0.89 |
| WSA | 0.691 | 45.45 | 0.92 | 0.602 | 43.16 | 0.86 |
| OSH | 0.717 | 43.30 | 0.77 | 0.746 | 47.78 | 0.75 |
| CSH | 0.787 | 17.25 | 0.94 | 0.799 | 19.29 | 0.78 |
| Overall | 0.771 | 46.18 | 0.88 | 0.711 | 47.99 | 0.79 |

**Table S8**. Variation of σ values (fitted based on MODIS satellite data at Flux sites) with environmental conditions. **Abbreviations:** **GST**, Growing season length (>0℃); **PPFD**, Photosynthetic photon flux density; **SW**, Soil water content (%); **CSH**, Closed shrubland; **DBF**, Deciduous broadleaf; **EBF**, Evergreen broadleaf; **ENF**, Evergreen needleaf; **GRA**, Grassland; **MF,** Mixed Forest; **OSH**, Open shrubland; **SAV**, Savanna; **WSA**, Woody savanna.

|  | **MAT** | **p** | **Ln (PPFD)** | **p** | **Ln (SW)** | **p** | **RMSE** | **R^2^** |
| --- | --- | --- | --- | --- | --- | --- | --- | --- |
| MF | -0.008±0.004 | 0.06 | 0.047±0.036 | 0.20 | -0.042±0.029 | 0.16 | 0.06 | 0.14 |
| GRA | -0.007±0.003 | <0.05 | -0.067±0.083 | 0.42 | 0.008±0.028 | 0.77 | 0.16 | 0.12 |
| ENF | -0.033±0.003 | <0.001 | 0.395±0.046 | <0.001 | 0.002±0.019 | 0.89 | 0.13 | 0.49 |
| DBF | 0.003±0.002 | 0.25 | 0.171±0.038 | <0.001 | 0.0008±0.020 | 0.97 | 0.10 | 0.19 |
| SAV | 0.145±0.119 | 0.44 | -3.54±6.277 | 0.67 | -0.021±2.156 | 0.99 | 0.13 | 0.60 |
| EBF | 0.007±0.001 | <0.001 | -0.141±0.052 | <0.001 | 0.063±0.020 | <0.001 | 0.74 | 0.03 |
| WSA | 0.003±0.031 | <0.001 | -0.328±0.442 | 0.47 | 0.026±0.060 | 0.67 | 0.06 | 0.11 |
| OSH | -0.023±0.006 | <0.001 | 0.118±0.378 | 0.76 | 0.050±0.038 | 0.20 | 0.12 | 0.54 |
| CSH | 0.0008±0.023 | 0.97 | -0.269±0.317 | 0.43 | -0.034±0.0961 | 0.74 | 0.04 | 0.14 |
| Overall | -0.011 ± 0.001 | <0.001 | 0.192±0.027 | <0.001 | 0.045±0.010 | <0.001 | 0.14 | 0.49 |

**Table S9. Summary coefficients for Figure S12-S17, S21-S26.**

| Models | Inter-annual LAI variability | | | Intra-annual LAI variability | | |
| --- | --- | --- | --- | --- | --- | --- |
|  | RRMSE | bias | Correlation | RRMSE | bias | Correlation |
| Compared with MODIS-derived LAI | | | | | | |
| Our Model | 46.14 | 0.008 | 0.35 | 56.14 | -0.16 | 0.67 |
| CABLE-POP | 87.27 | 0.416 | 0.06 | 100.18 | 0.41 | 0.47 |
| CLASSIC | 119.25 | 0.45 | 0.26 | 96.71 | 0.39 | 0.61 |
| CLM 5.0 | 110.97 | 0.83 | 0.19 | 135.36 | 0.77 | 0.22 |
| IBIS | 147.04 | 1.22 | 0.19 | 164.55 | 1.15 | 0.41 |
| ISAM | 52.46 | 0.23 | 0.19 | 71.42 | 0.16 | 0.62 |
| ISBA-CTRIP | 49.67 | 0.20 | 0.25 | 64.06 | 0.14 | 0.60 |
| JSBACH | 47.30 | -0.03 | 0.22 | 63.11 | -0.10 | 0.57 |
| JULES-ES | 113.86 | 0.89 | 0.22 | 123.62 | 0.83 | 0.43 |
| LPJ-GUESS | 81.46 | 0.65 | 0.14 | 92.92 | 0.58 | 0.66 |
| LPX-Bern | 158.27 | 0.91 | 0.15 | 163.53 | 0.85 | 0.66 |
| OCN | 117.90 | 1.01 | 0.27 | 126.97 | 0.94 | 0.52 |
| ORCHIDEEv3 | 54.81 | 0.15 | 0.23 | 73.36 | 0.09 | 0.54 |
| SDGVM | 181.96 | 1.46 | 0.17 | 190.92 | 1.40 | 0.52 |
| VISIT | 256.19 | 1.66 | 0.19 | 257.76 | 1.63 | 0.49 |
| YIBs | 56.77 | 0.11 | 0.15 | 72.85 | 0.04 | 0.62 |
| Compared with Copernicus-derived LAI | | | | | | |
| Our Model | 53.84 | -0.005 | 0.36 | 64.92 | -0.11 | 0.63 |
| CABLE-POP | 75.78 | 0.39 | 0.09 | 102.87 | 0.39 | 0.38 |
| CLASSIC | 80.71 | 0.35 | 0.28 | 99.77 | 0.42 | 0.55 |
| CLM 5.0 | 104.51 | 0.66 | 0.22 | 138.77 | 0.68 | 0.15 |
| IBIS | 147.01 | 0.91 | 0.26 | 169.82 | 0.93 | 0.35 |
| ISAM | 61.05 | 0.20 | 0.12 | 87.16 | 0.24 | 0.53 |
| ISBA-CTRIP | 46.29 | 0.14 | 0.27 | 70.67 | 0.16 | 0.56 |
| JSBACH | 52.14 | -0.002 | 0.16 | 73.75 | 0.04 | 0.53 |
| JULES-ES | 111.71 | 0.59 | 0.24 | 124.70 | 0.64 | 0.36 |
| LPJ-GUESS | 83.73 | 0.60 | 0.19 | 103.51 | 0.62 | 0.62 |
| LPX-Bern | 83.73 | 0.60 | 0.19 | 172.98 | 0.53 | 0.62 |
| OCN | 114.62 | 0.88 | 0.28 | 135.01 | 0.89 | 0.45 |
| ORCHIDEEv3 | 51.63 | 0.11 | 0.27 | 71.94 | 0.13 | 0.50 |
| SDGVM | 155.37 | 1.10 | 0.17 | 201.16 | 1.08 | 0.47 |
| VISIT | 169.81 | 1.52 | 0.23 | 257.84 | 0.94 | 0.43 |
| YIBs | 57.99 | 0.12 | 0.21 | 77.71 | 0.11 | 0.59 |

**Figure S1**. RRMSE, R^2^ and bias error distributions of different vegetation types according to different lengths of acclimation time (represented by parameter α) using the exponential moving average approach. The statistics (RMSE, R^2^, and bias) of different vegetation types were calculated mainly through three steps: (1) Calculate RRMSE, R^2^, and bias for daily LAI of every week during growing season at each site. (2) Calculate the median values of RRMSE, R^2^, and bias among all weeks for each site to remove extreme values. (3) Calculate the mean values of RRMSE, R^2^, and bias among sites for each vegetation type. **Abbreviations**: **CSH**, Closed shrubland; **DBF**, Deciduous broadleaf; **EBF**, Evergreen broadleaf; **ENF**, Evergreen needleaf; **GRA**, Grassland; **MF,** Mixed Forest; **OSH**, Open shrubland; **SAV**, Savanna; **WSA**, Woody savanna.


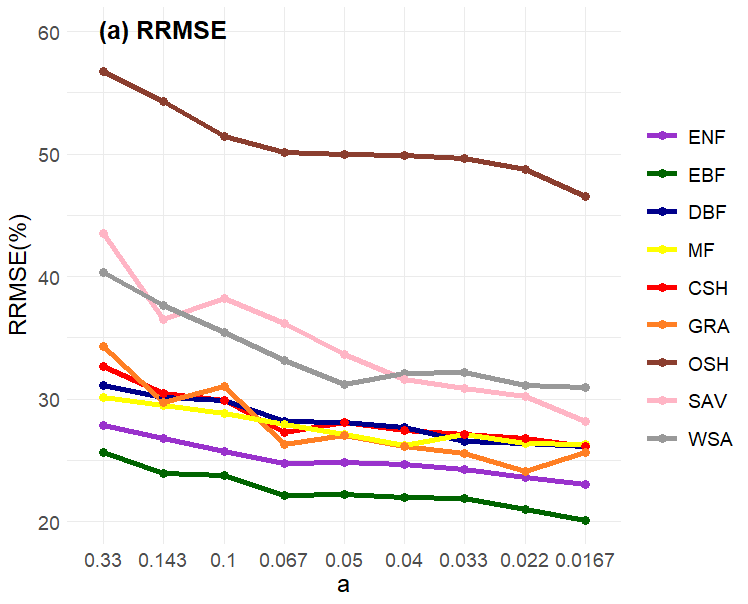

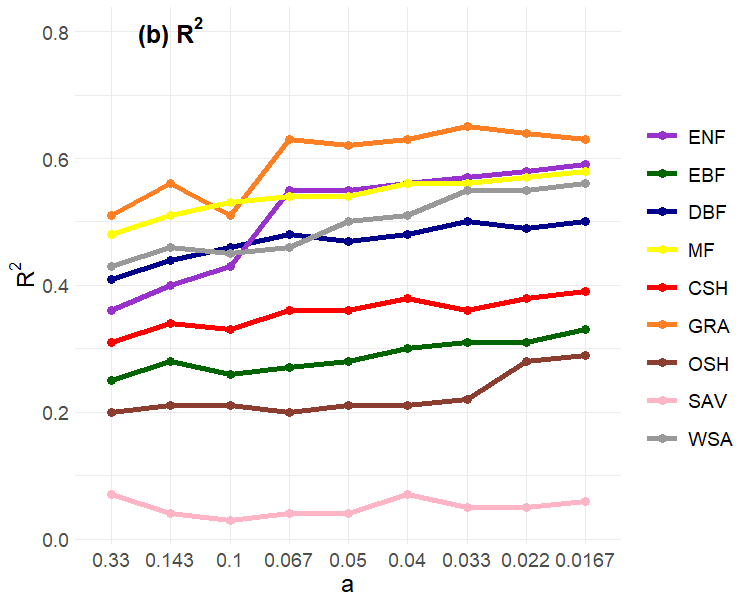

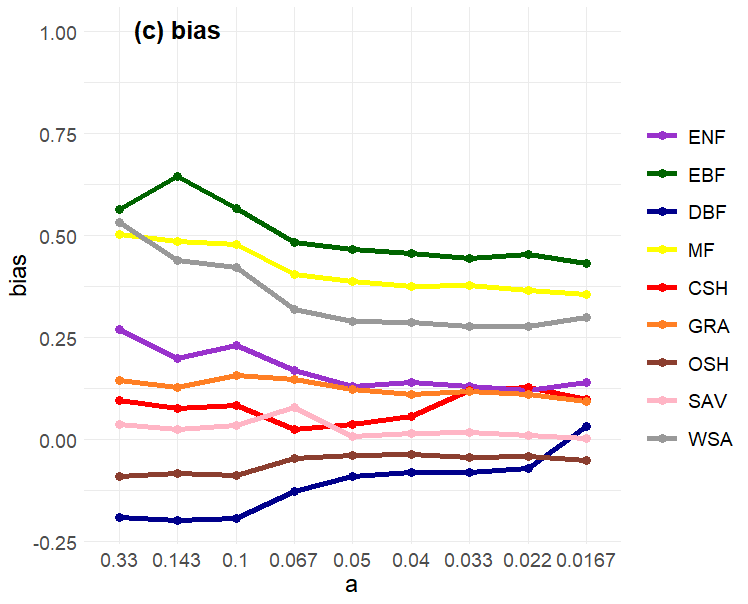


**Figure S2** Mean seasonal cycle of LAI. Observations are given by the black line (Copernicus LAI data) and grey band, representing the median and 33 %/66 % quantiles of all data (multiple sites and years) pooled by vegetation type. Red line (Model_Prognostic_) is the seasonal variation of simulated LAI across all sites and years for each vegetation type, where simulated LAI are calculated using P model derived-GPP and seasonal maximum LAI from fAPAR_max_ model as inputs. Orange line (Model_Flux_) is the seasonal variation of simulated LAI across all sites and years for each vegetation type, where simulated LAI are calculated using Flux tower GPP and annual peak Copernicus LAI as inputs. **Abbreviations**: **CSH**, Closed shrubland; **DBF**, Deciduous broadleaf; **EBF**, Evergreen broadleaf; **ENF**, Evergreen needleaf; **GRA**, Grassland; **MF,** Mixed Forest; **OSH**, Open shrubland; **SAV**, Savanna; **WSA**, Woody savanna.

**
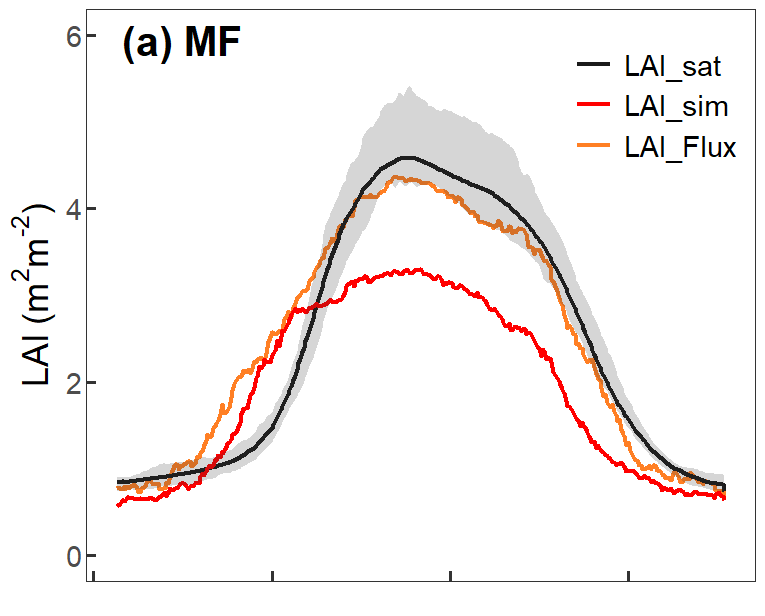

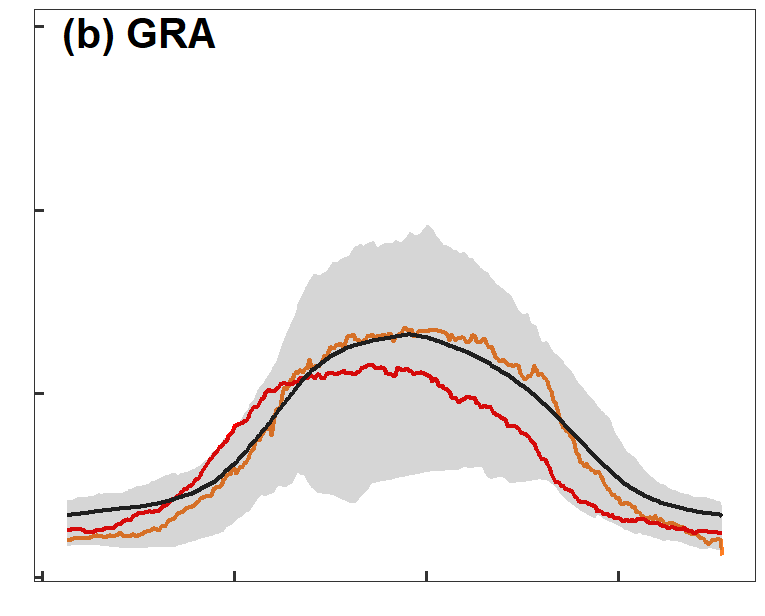

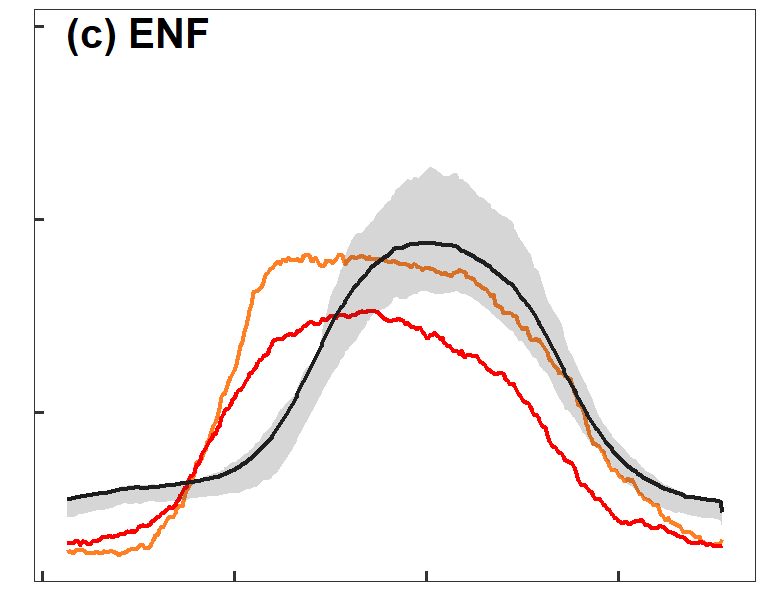
**

**
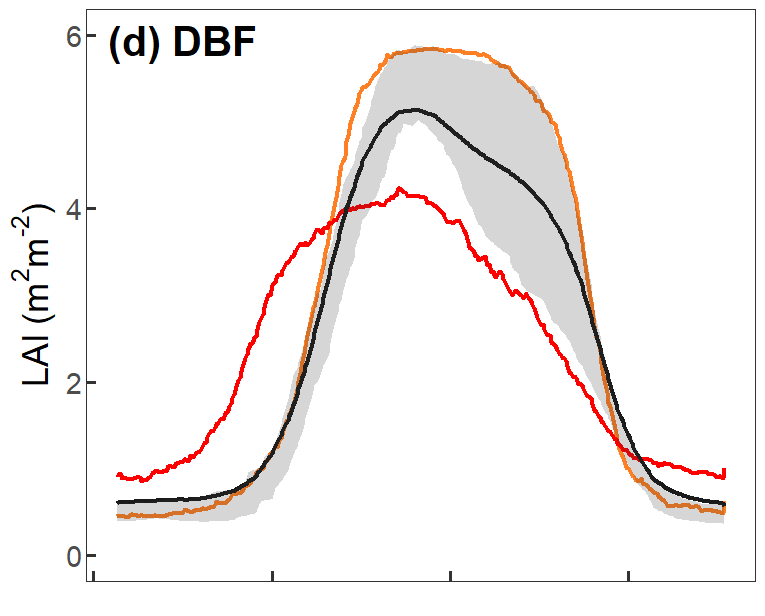

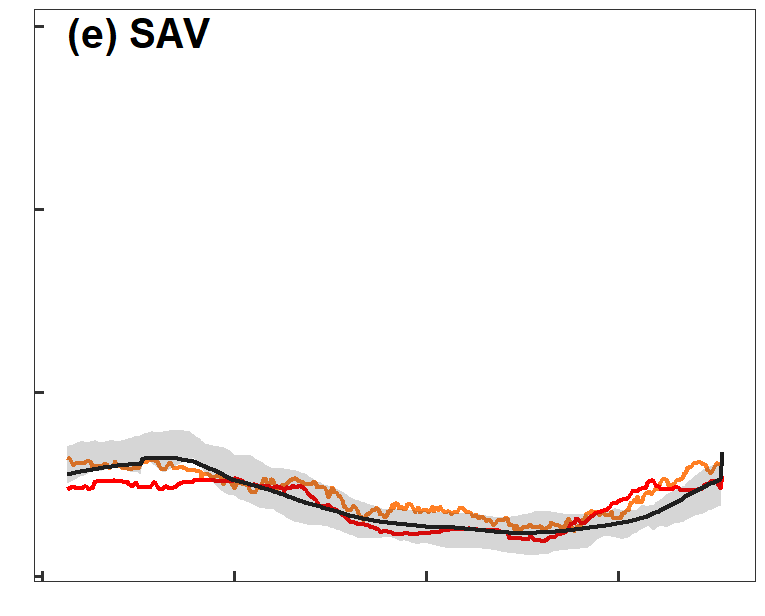

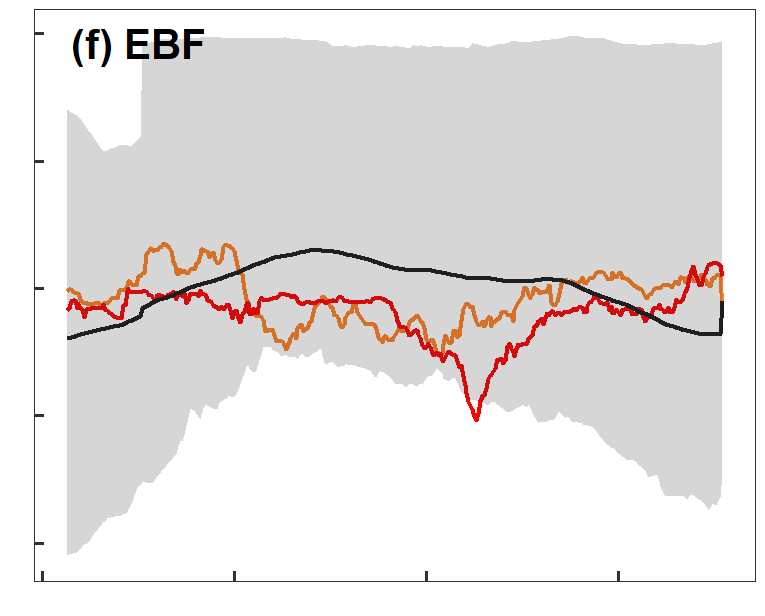
**

**
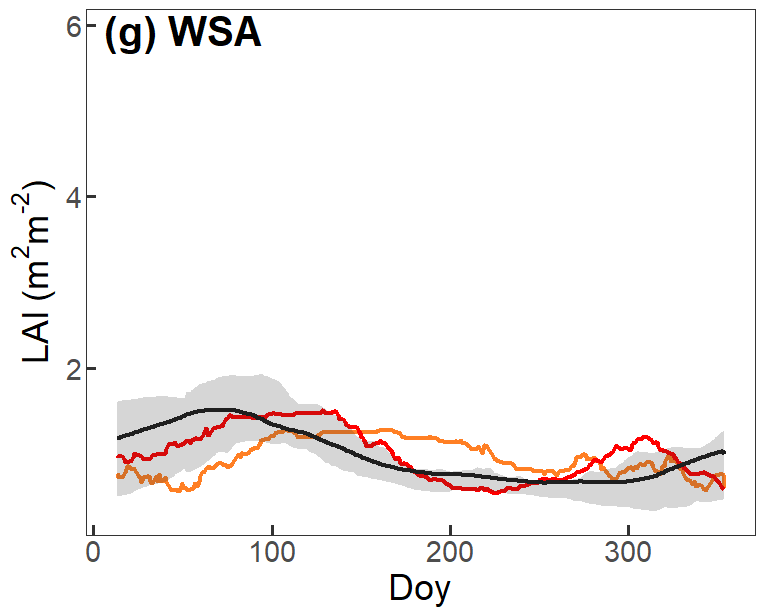

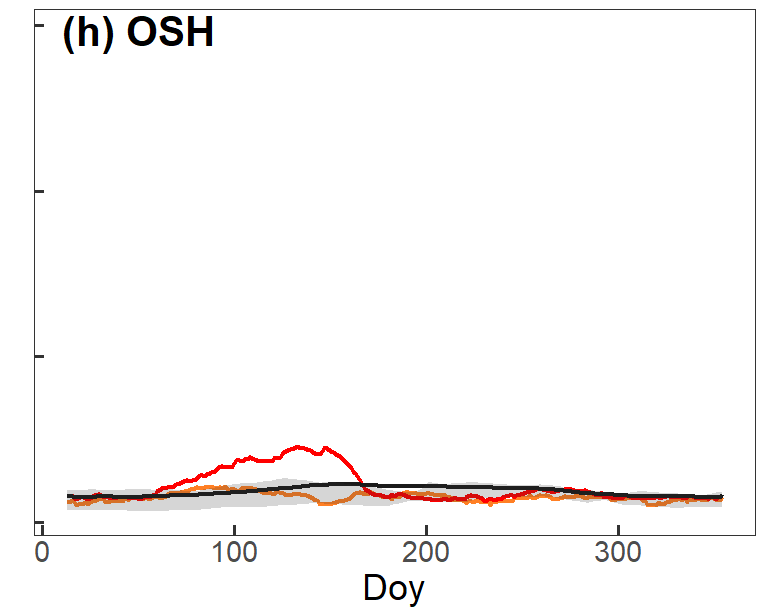

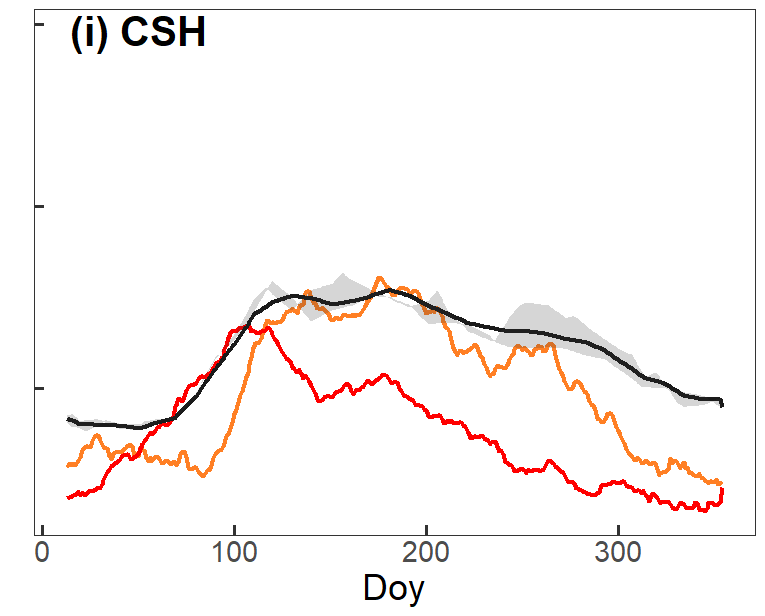
**

**Figure S3** Regression analysis is shown for satellite-derived (Copernicus) and simulated yearly average LAI for all vegetation types together, where simulated LAI are calculated using P model derived-GPP and seasonal maximum LAI from fAPAR_max_ model as inputs (Model_Prognostic_).


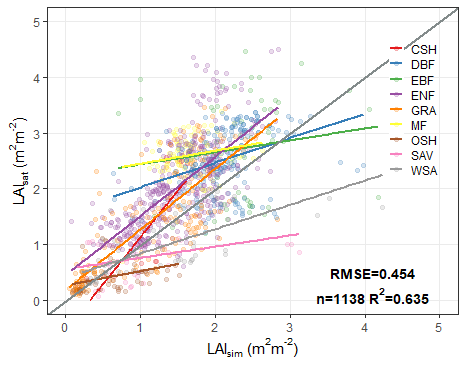


**Figure S4** The spatial distribution of σ, inferred from (a) the MODIS fAPAR and LAI products, (b) AVHRR fAPAR and LAI products, (c) GLOBMAP fAPAR and LAI products. The histograms show the distribution of *σ* values. Cropland, snow/ice, and non-vegetated areas are shown in white.


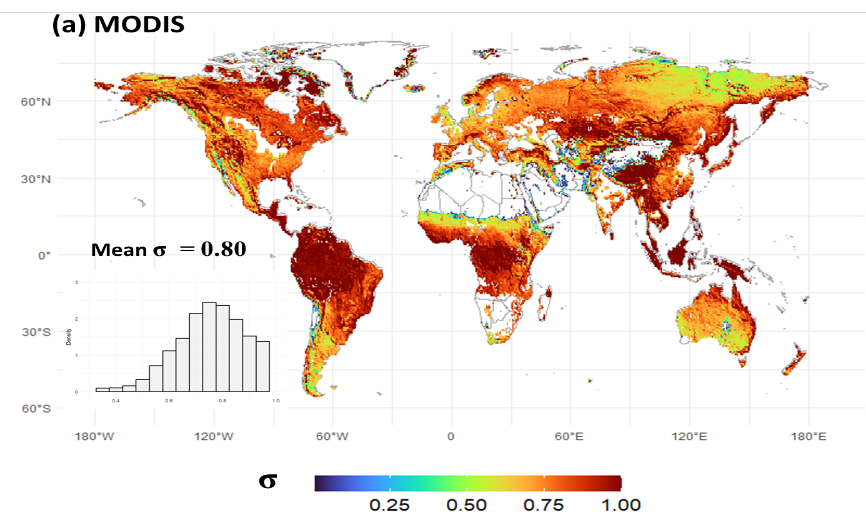

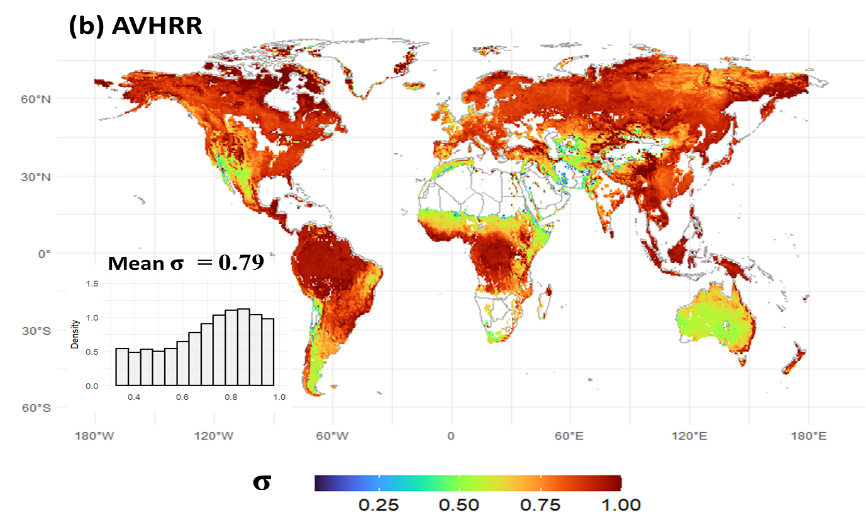


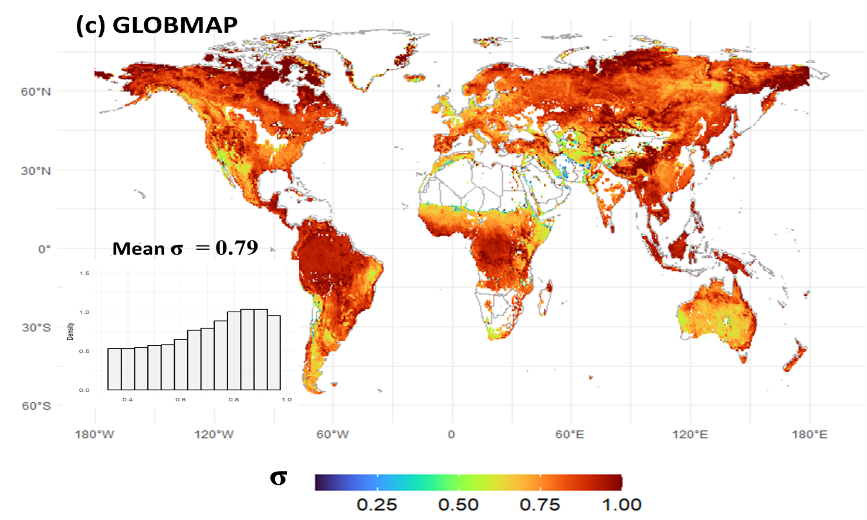


**Figure S5** Correlation of MODIS-derived and modelled annual mean LAI values for different model combinations: (a) Model_Prognostic_, Simulated LAI dynamics using P model derived GPP and simulated seasonal maximum LAI from fAPAR_max_ model as inputs; (b) Model_Flux_, Simulated LAI dynamics using Flux tower GPP and seasonal maximum satellite LAI as inputs. **Spatial:** The red lines are the regression between multi-year observed and multi-year modelled LAI means aggregated from all sites, representing spatial (across-site) variations. **Annual:** Black lines are based on annual LAI values, with one line for each site, representing the linear regression model of annual LAI values for a single site. For a perfect fit between modelled and observed values, all black lines would lie on the 1:1 line (grey dashed line) and have a slope of 1. For each kind of linear regression model (Spatial and Annual), R^2^ and RMSE statistics are calculated.


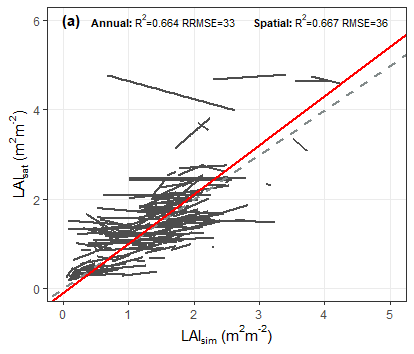

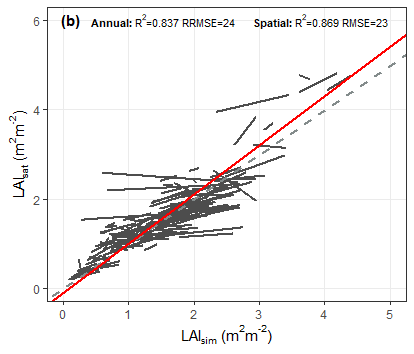


**Figure S6** The spatial distributions of multiyear average LAI (2000 to 2019) using σ, inferred from (a) Flux tower-derived GPP data and MODIS derived fAPAR and LAI data; (b) Flux-tower-derived GPP data and Copernicus derived fAPAR and LAI data; (c) P model-derived GPP data and MODIS fAPAR and LAI products, and (d) P model-derived GPP data and AVHRR/GLOBMAP fAPAR and LAI products.


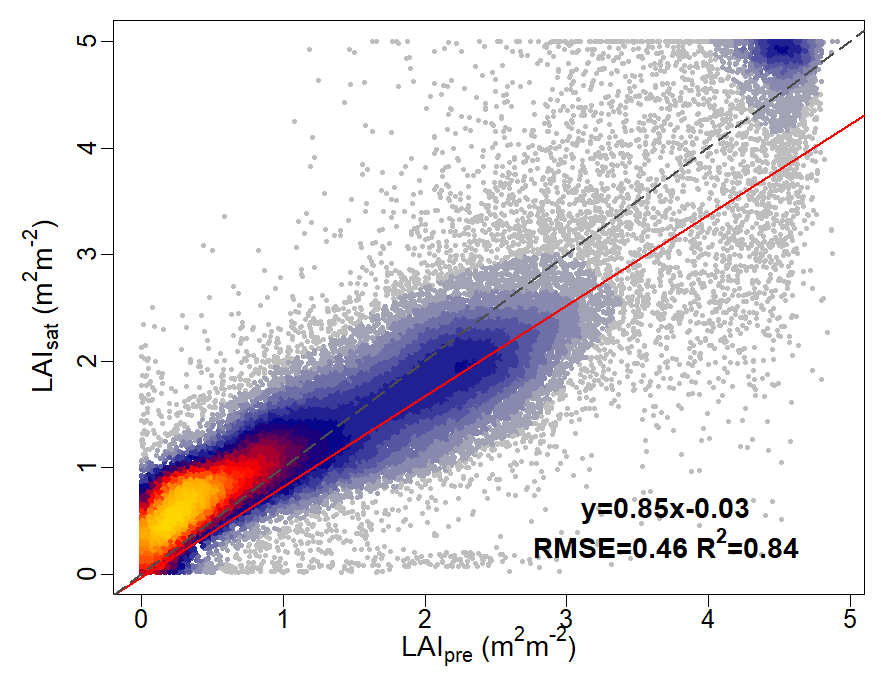

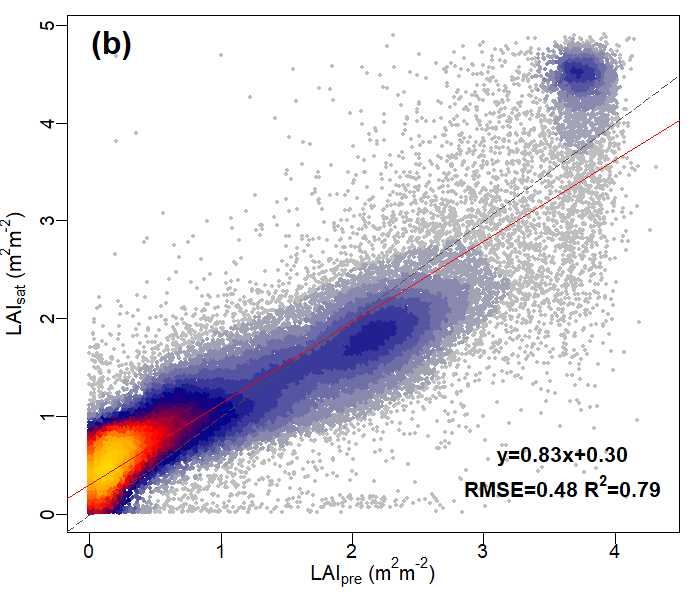


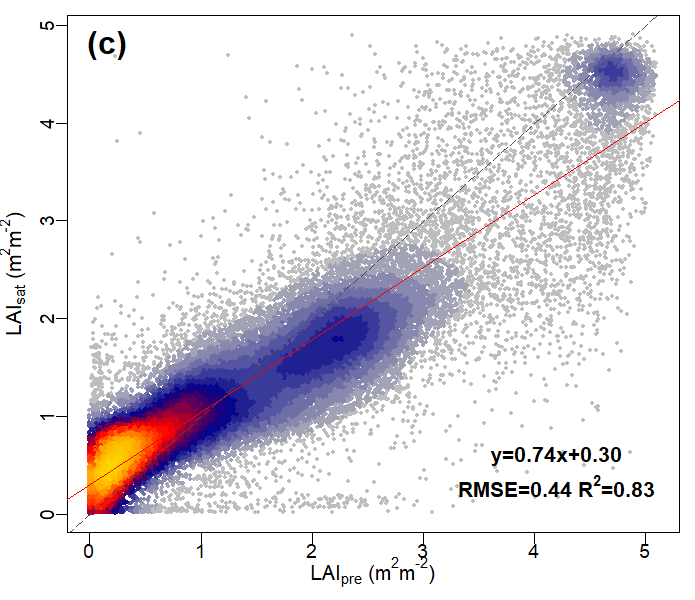

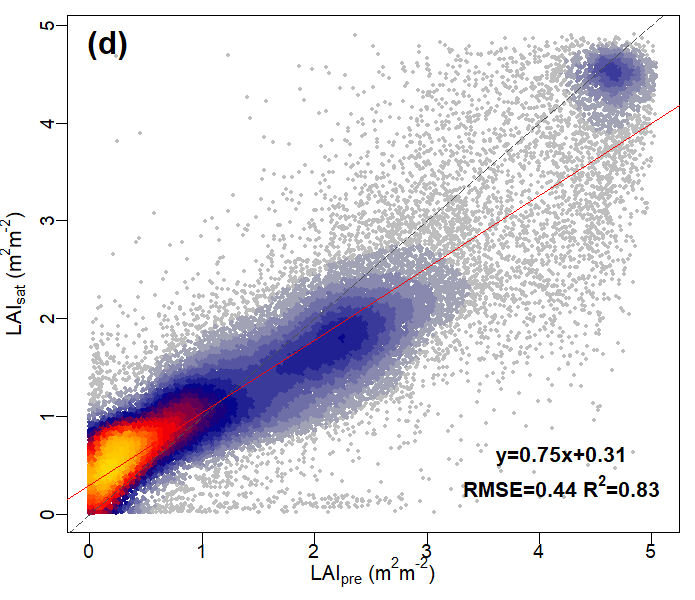


**Figure S7** Mean seasonal cycle of potential GPP. Observations are given by the dark blue line **(*A_0_obs_*)** and grey band, representing the median and 33 %/66 % quantiles of all data (multiple sites and years) pooled by vegetation type. Light blue line **(*A_0_sim_*)** is the seasonal variation of simulated potential GPP across all sites and years for each vegetation type. **Abbreviations**: **CSH**, Closed shrubland; **DBF**, Deciduous broadleaf; **EBF**, Evergreen broadleaf; **ENF**, Evergreen needleaf; **GRA**, Grassland; **MF,** Mixed Forest; **OSH**, Open shrubland; **SAV**, Savanna; **WSA**, Woody savanna. Each point represents the site-year data.


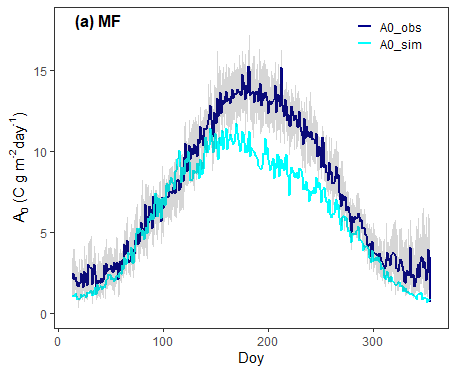

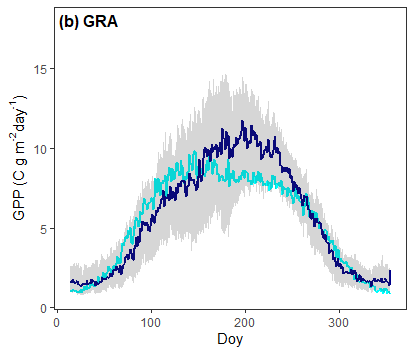

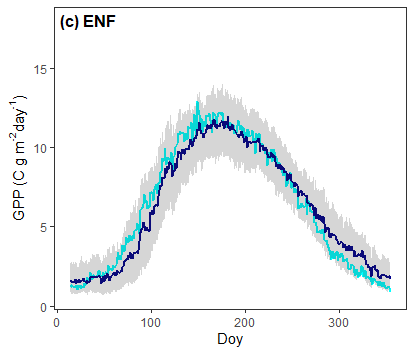


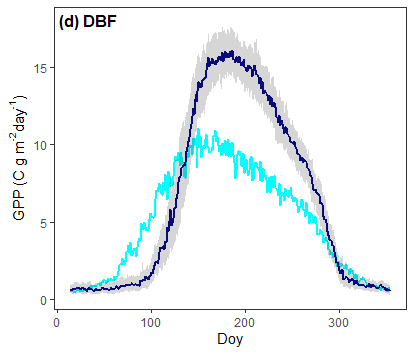

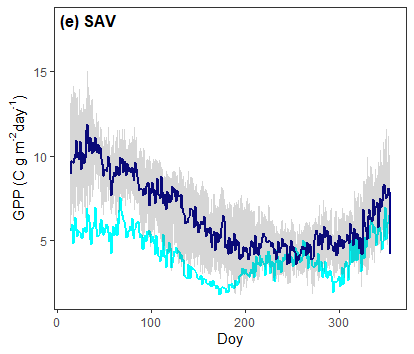

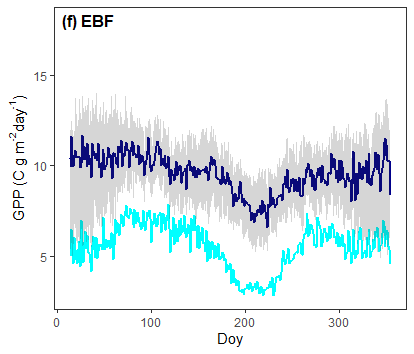


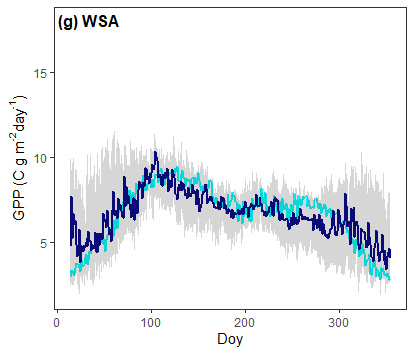

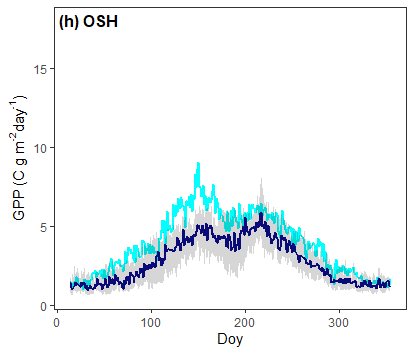

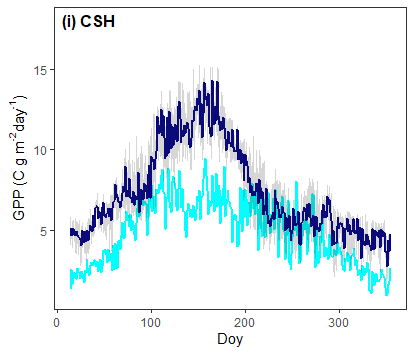


**Fig. S8.** Comparison of (a) predicted multiyear mean LAI_max_ between 2001-2019 based on an empirical equation of *f_0_* (Eqn. S3) of *f_0_* and (b) a single constant value with (c) LAI_max_ derived from MODIS LAI products and their latitudinal variation. (d) is the spatial distribution of simulated *f_0_* (Eqn.S3). The red and blue lines in insert figures of (c) represent the latitudinal variation of predicted LAI_max_ based on an empirical equation of *f_0_* (red), a single constant value (blue) and MODIS LAI products (black) respectively. Cropland, snow/ice, and non-vegetated areas are shown in white.

**
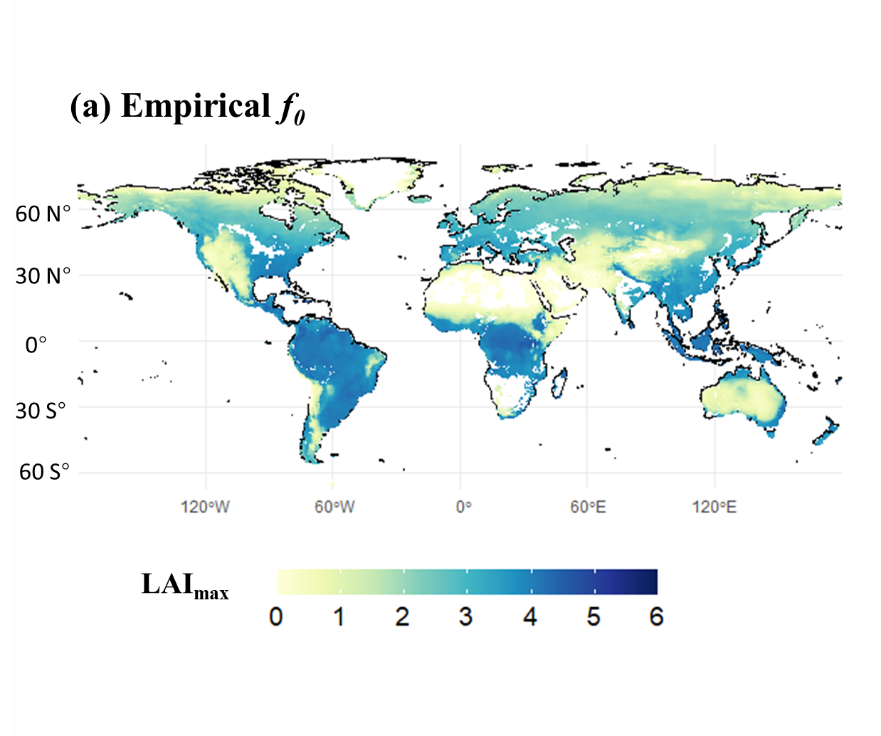

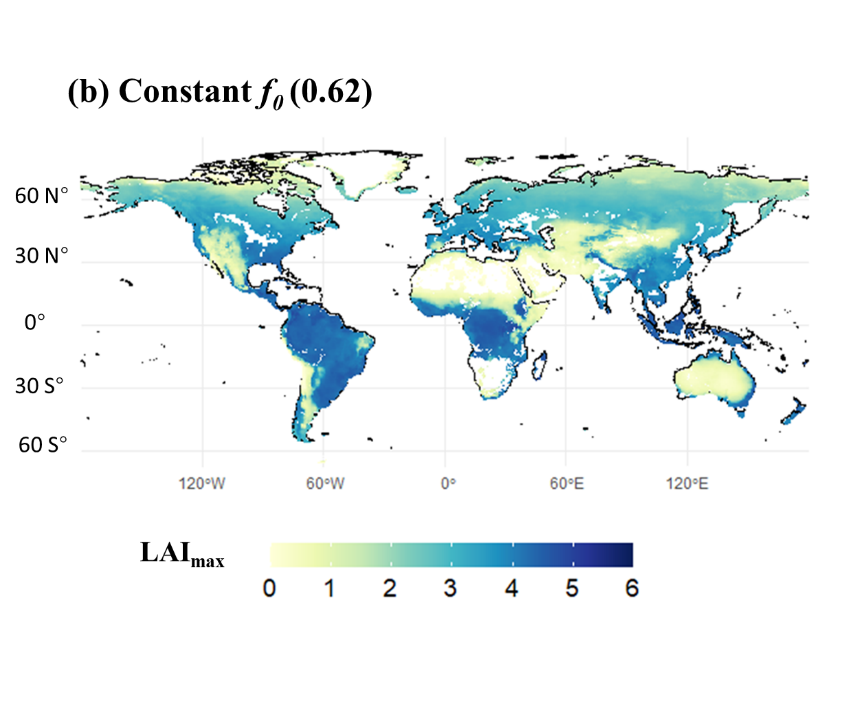
**

**
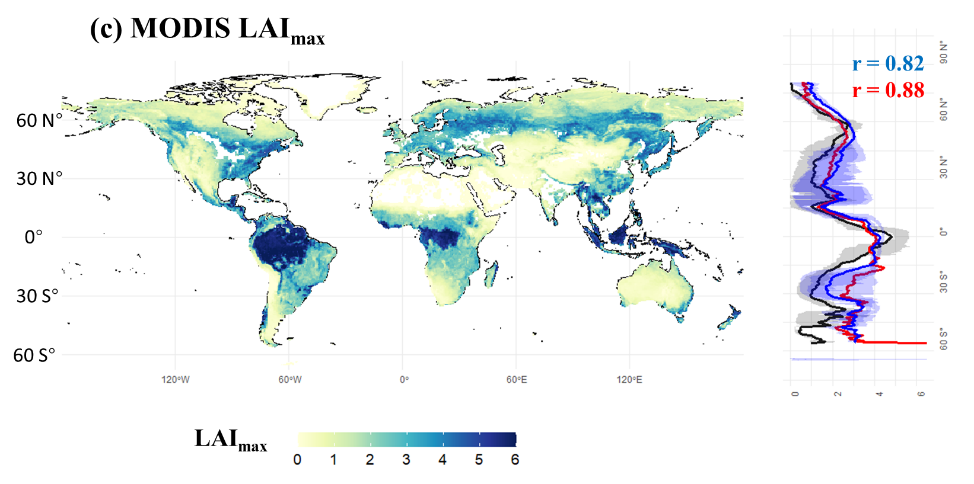

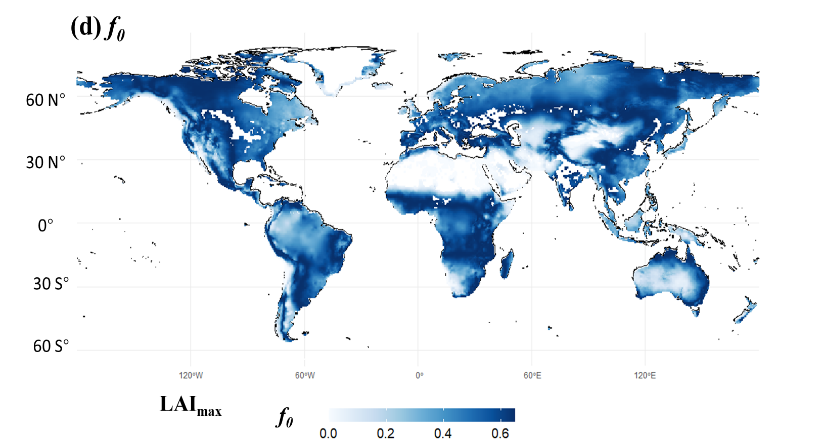
**

**Figure.S9** Differences between simulated and MODIS-derived multi-year Leaf Area Index (LAI) mean values (2001-2019) (Simulated LAI – MODIS-derived LAI). The insert histogram showed the distribution of differences between intervals. The blue dashed line indicates ± 0.56 intervals, while red dashed line indicates ± 1.12 intervals. Cropland, snow/ice, and non-vegetated areas are shown in white.


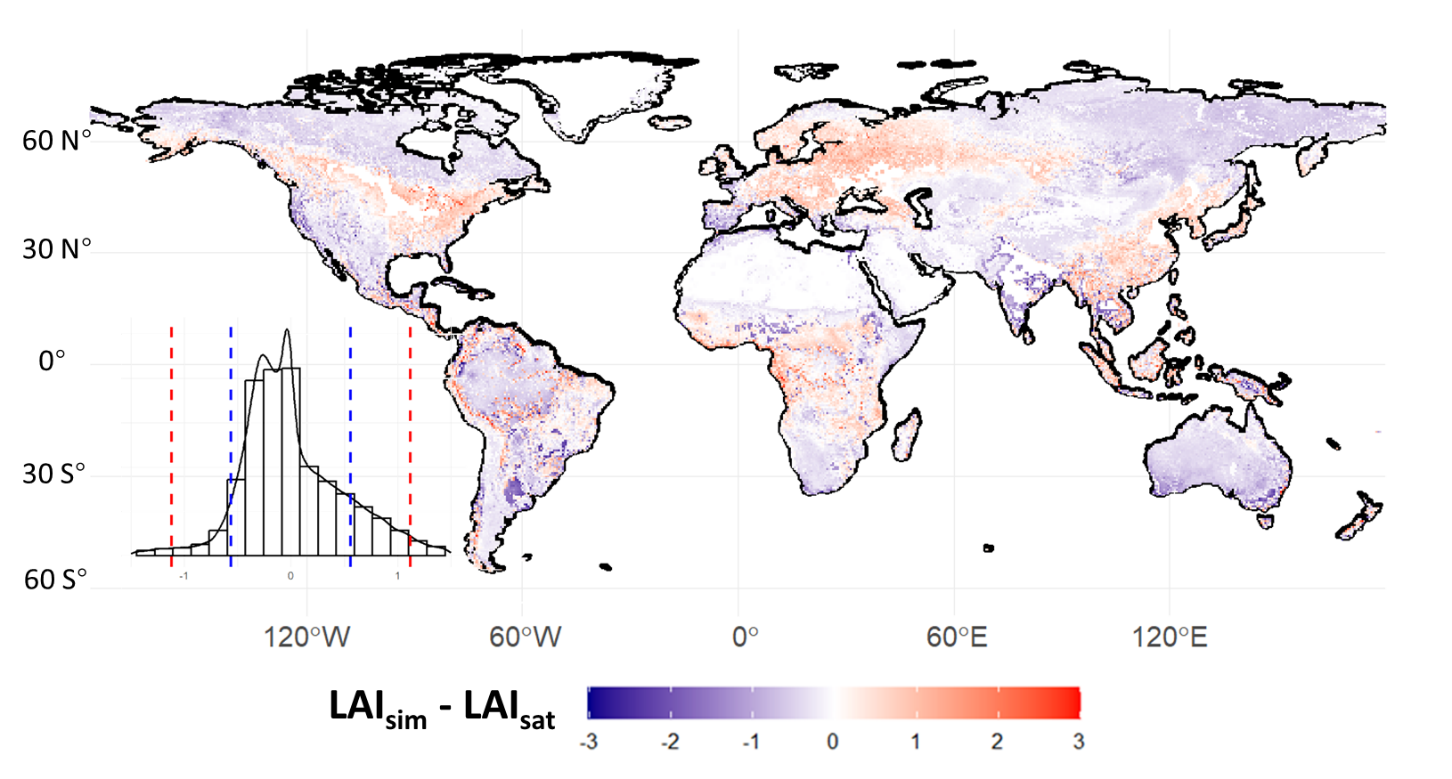


**Figure.S10** The seasonal differences between simulated and MODIS-derived Leaf Area Index (LAI) values (2001-2019) (Simulated LAI – MODIS-derived LAI) on a global scale. Blue means underestimation of monthly mean LAI, red colour means overestimation of monthly mean LAI. Cropland, snow/ice, and non-vegetated areas are shown in white.

**
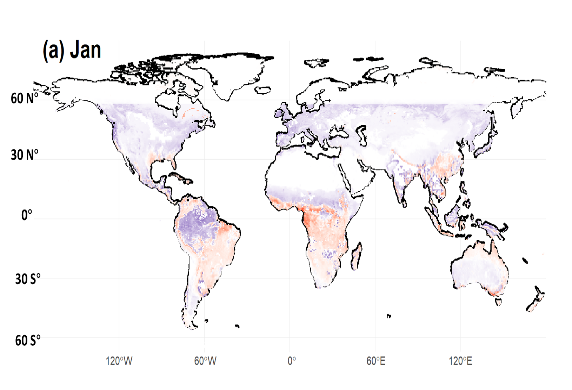

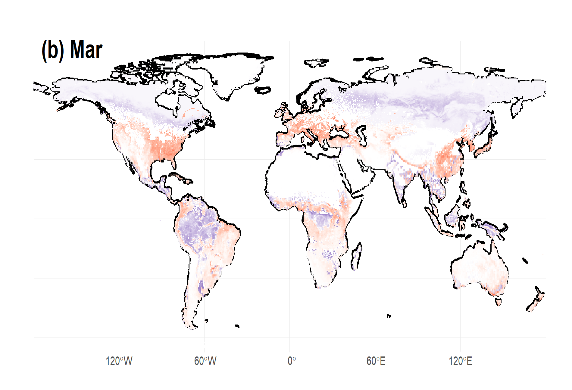

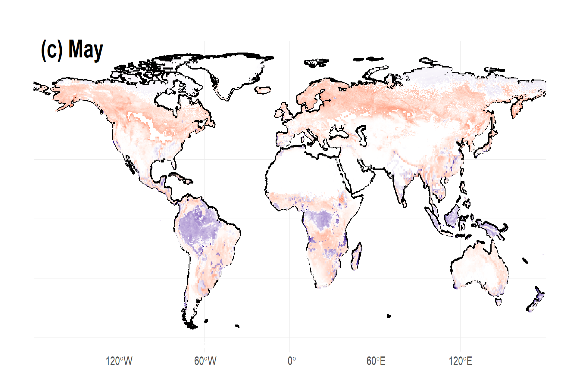
**

**
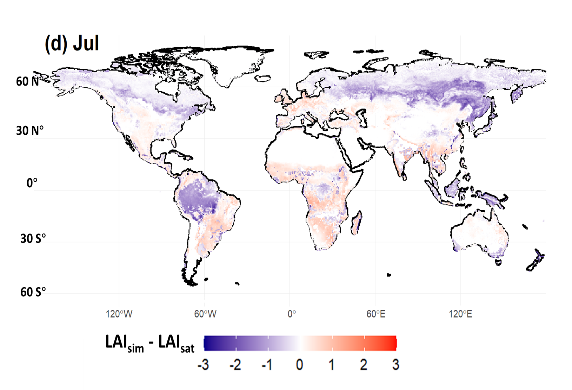

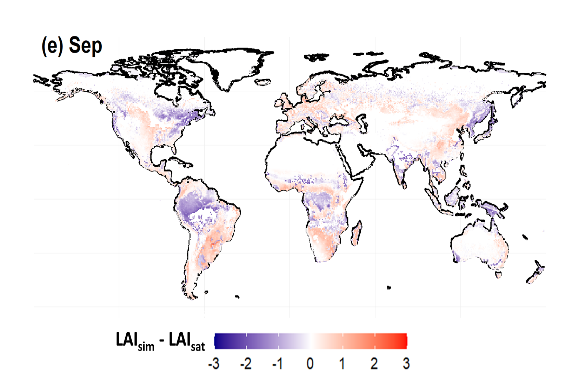

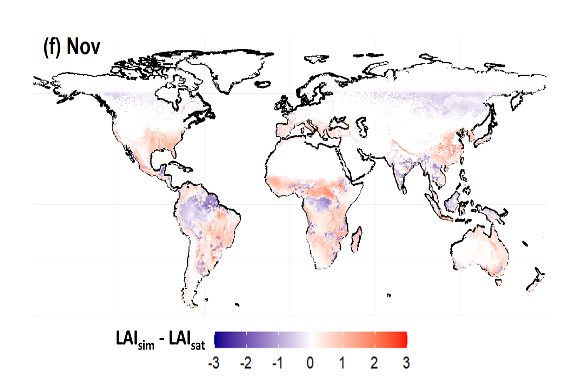
**

**Figure.S11** Regression analysis of multi-year average of simulated LAI and observed LAI from MODIS (2001 to 2019). (a) is the regression between our model and observed LAI from MODIS; (b)-(p) are the regression between other 15 models of Trendy project and observed LAI from MODIS. The red solid line is 1:1 line; the black dashed line is the regression line. RMSE: root-mean-squared error of prediction; R^2^: proportion of observed variance accounted for by the prediction.


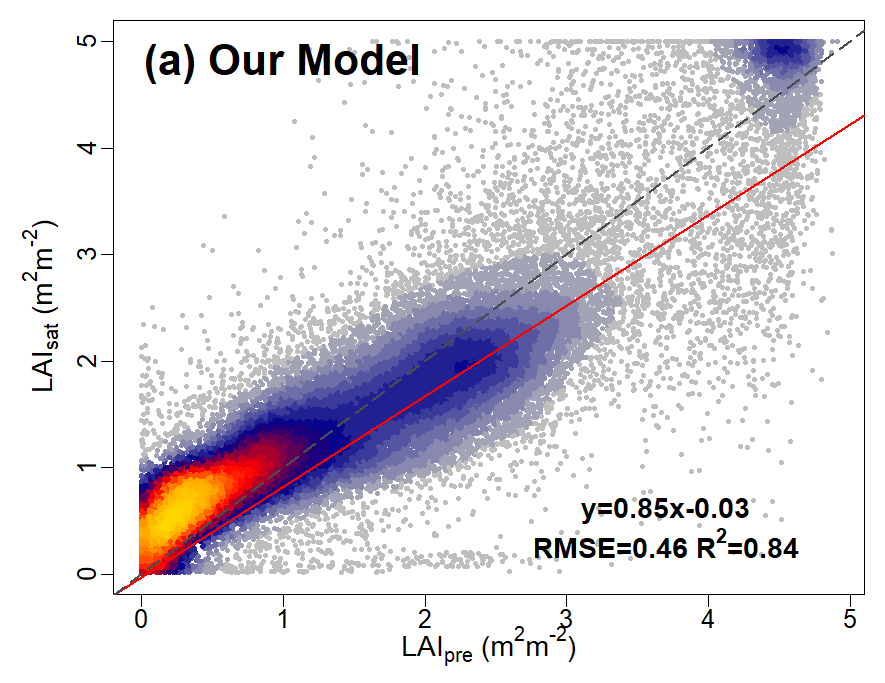

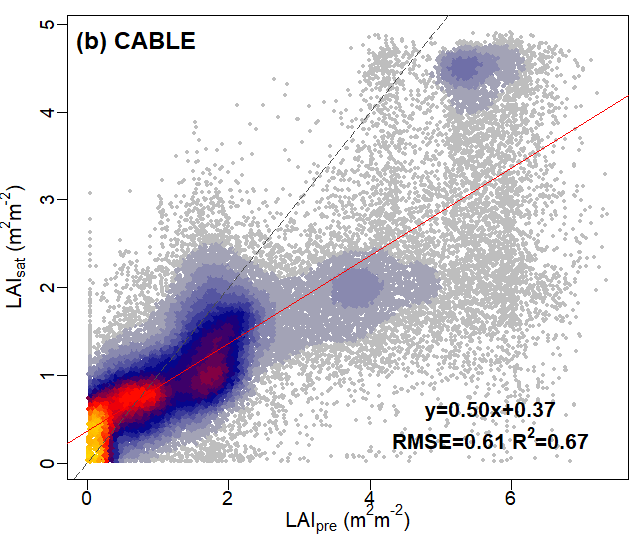

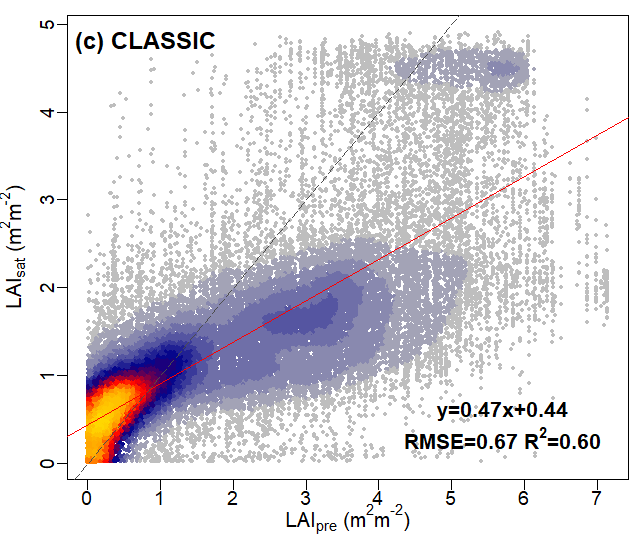

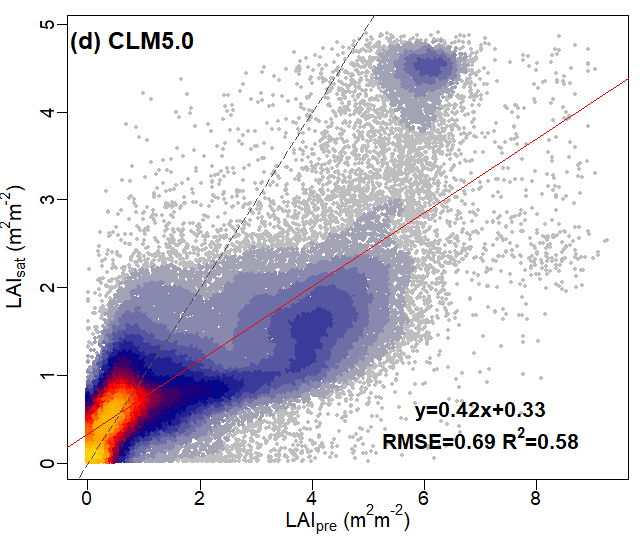


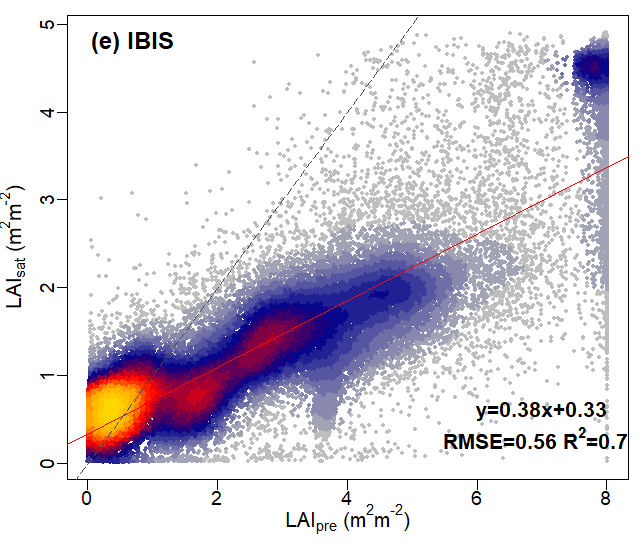

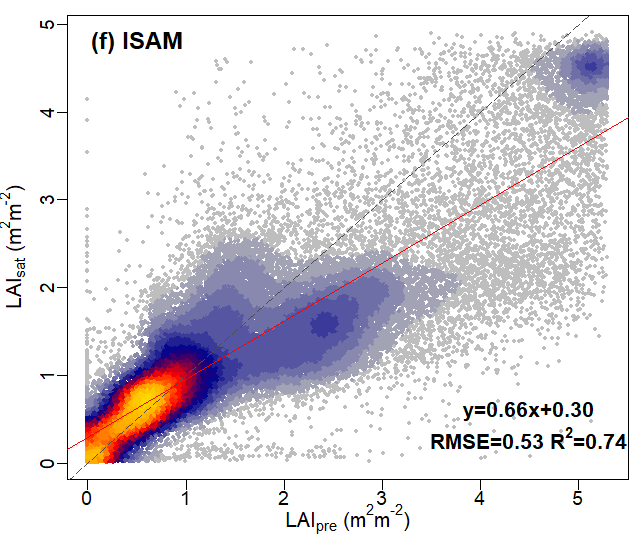

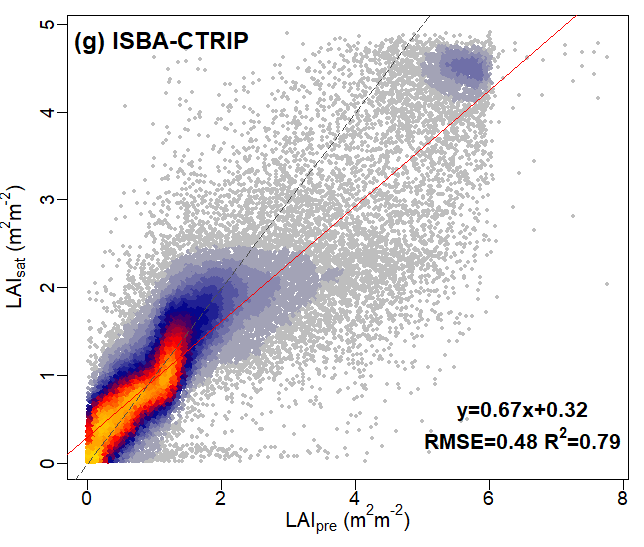

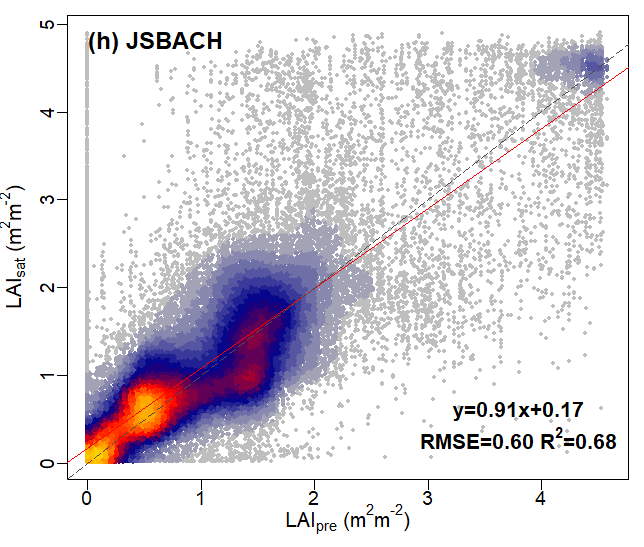


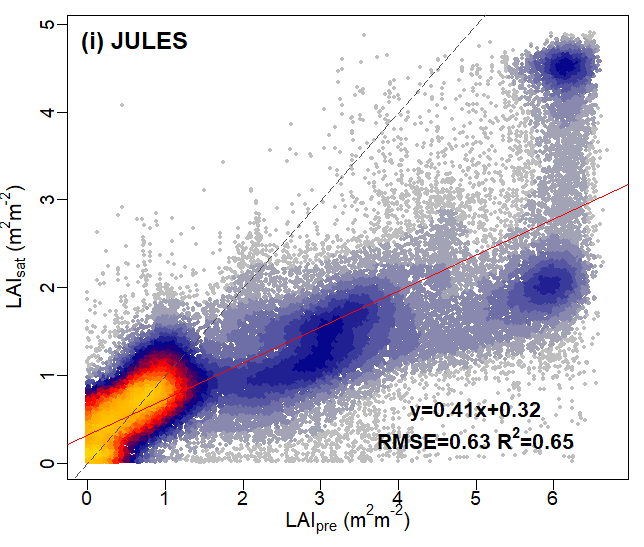

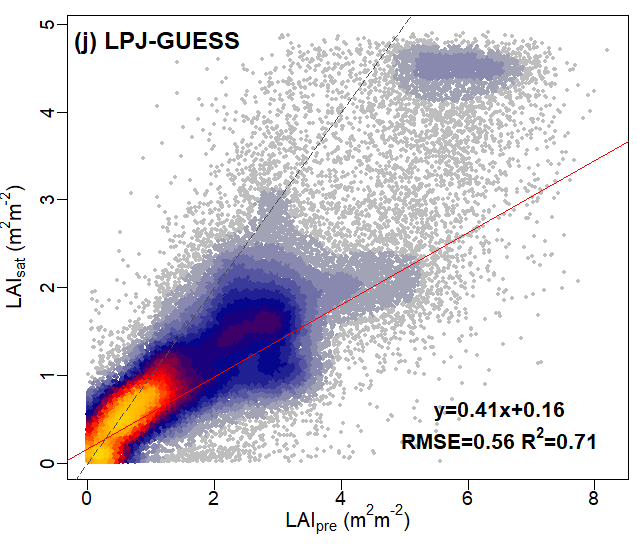

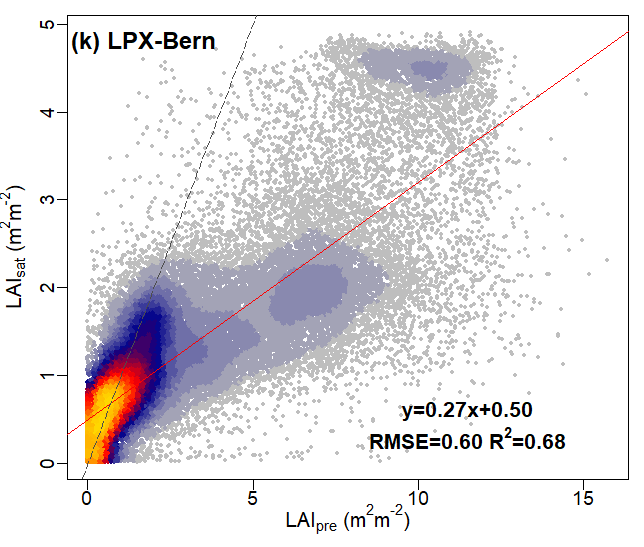

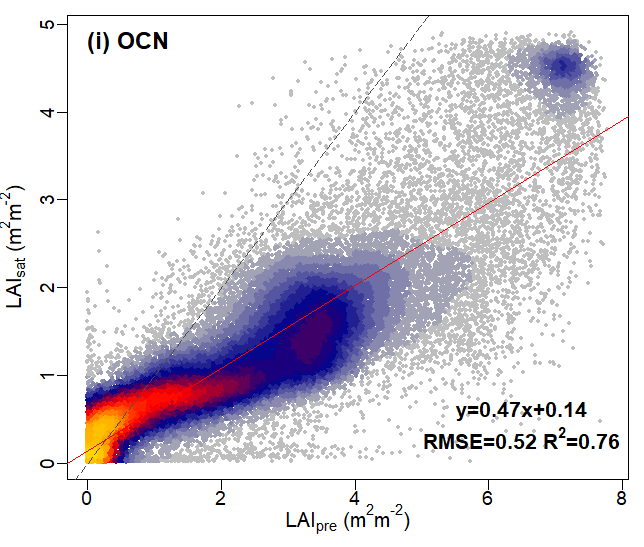


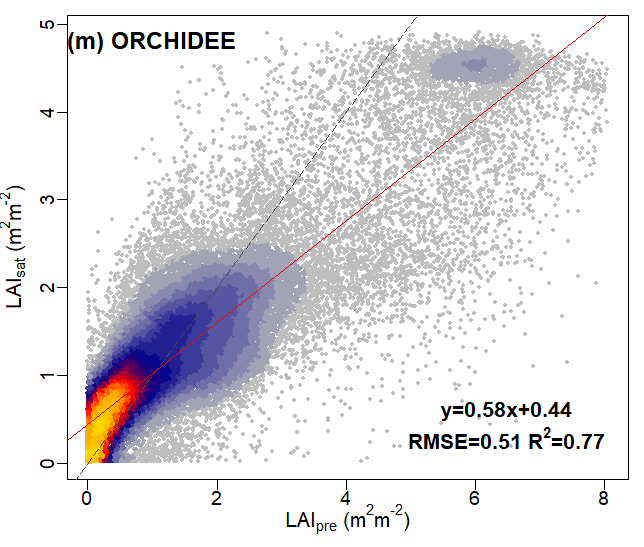

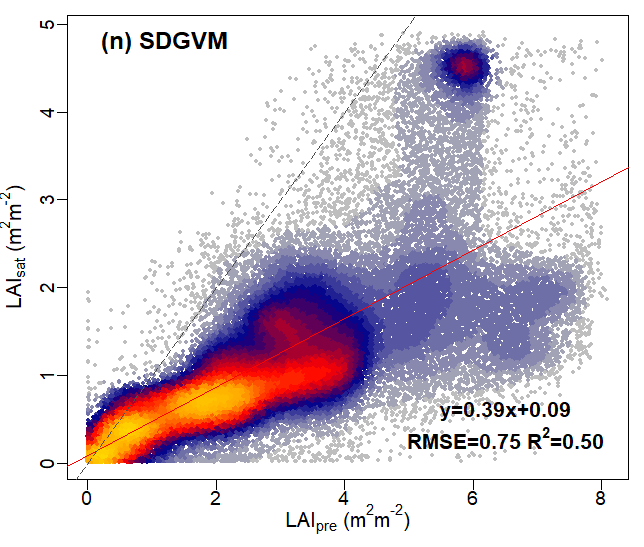

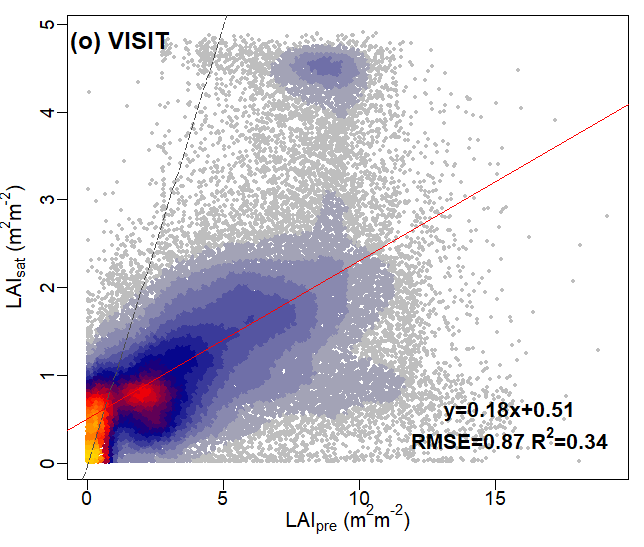

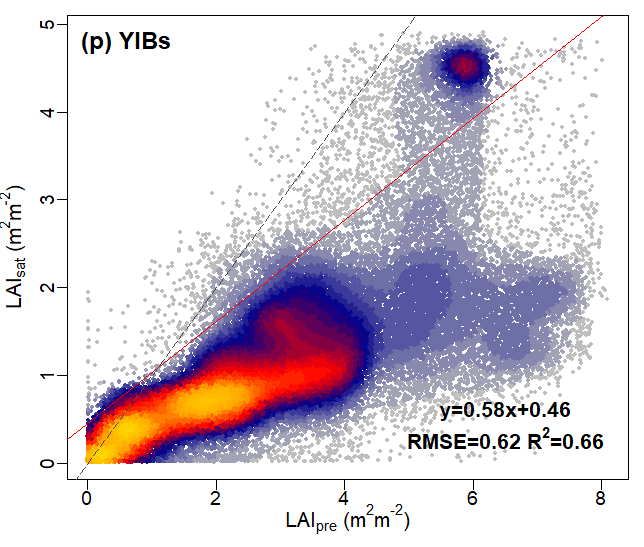


**Fig**.**S12** The spatial distribution of relative root mean squared error (RRMSE) between modelled and MODIS-derived annual average LAI time series (from 2001 to 2019). (a) is the RRMSE between our model and satellite products; (b)-(p) are the RRMSE between other 15 models of Trendy project and satellite products. Cropland, snow/ice, and non-vegetated areas are shown in white.


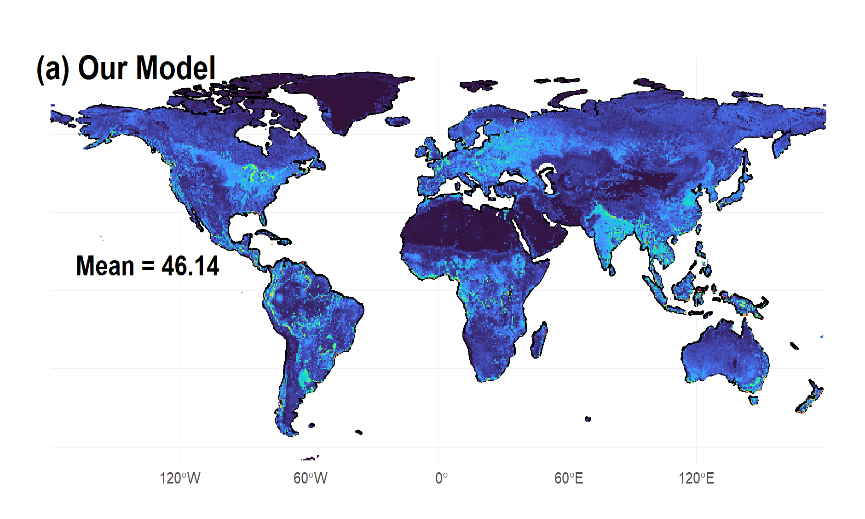

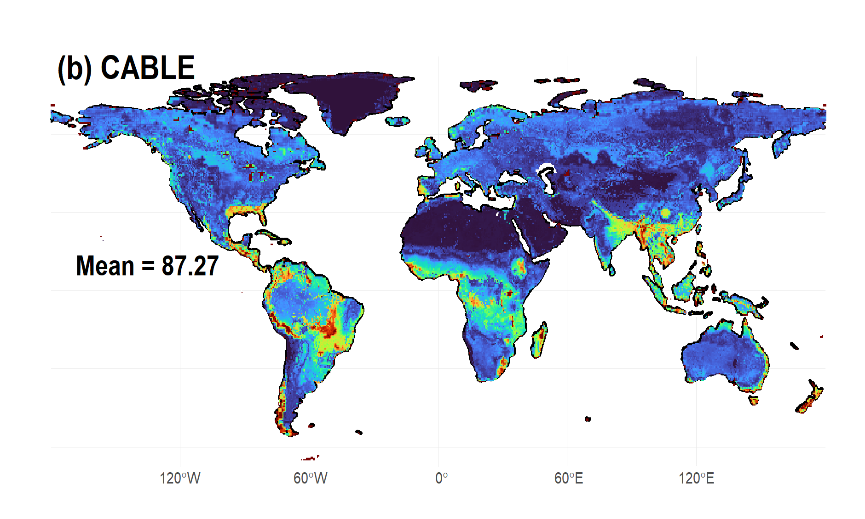


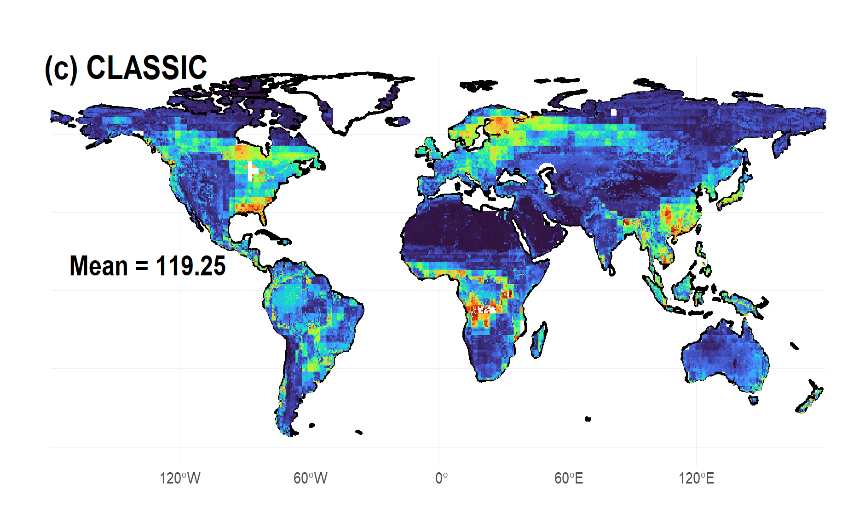

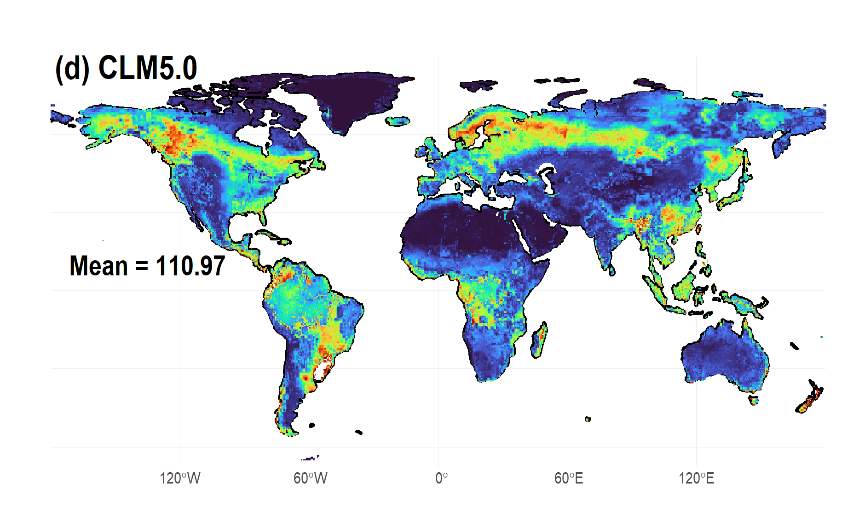


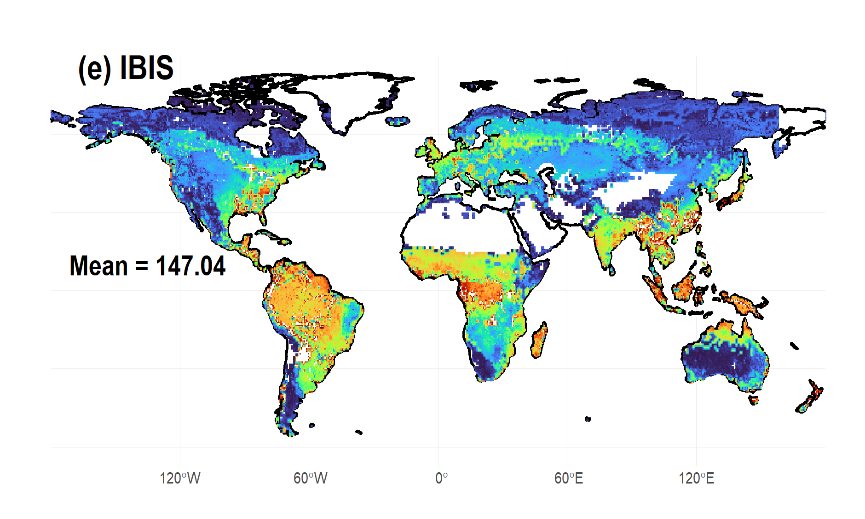

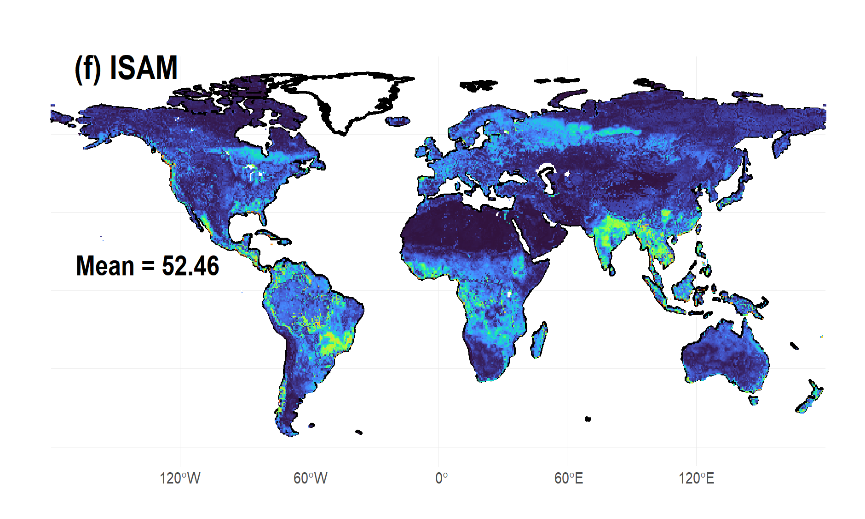


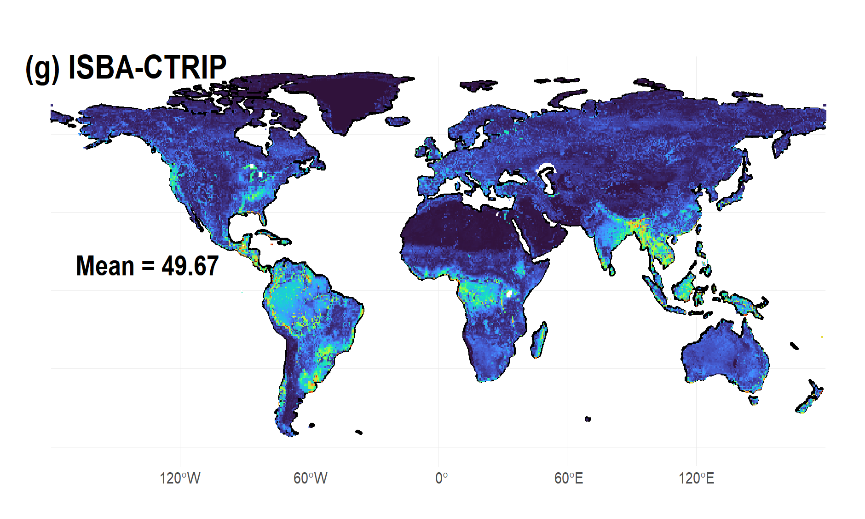

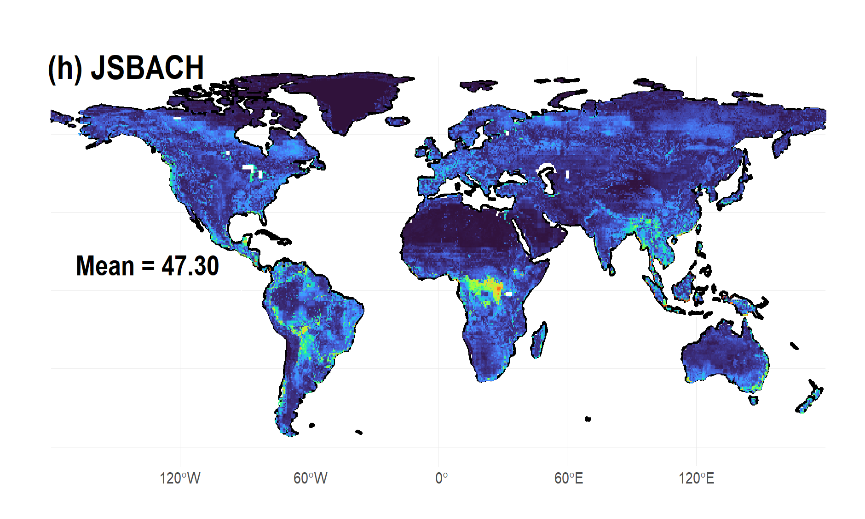


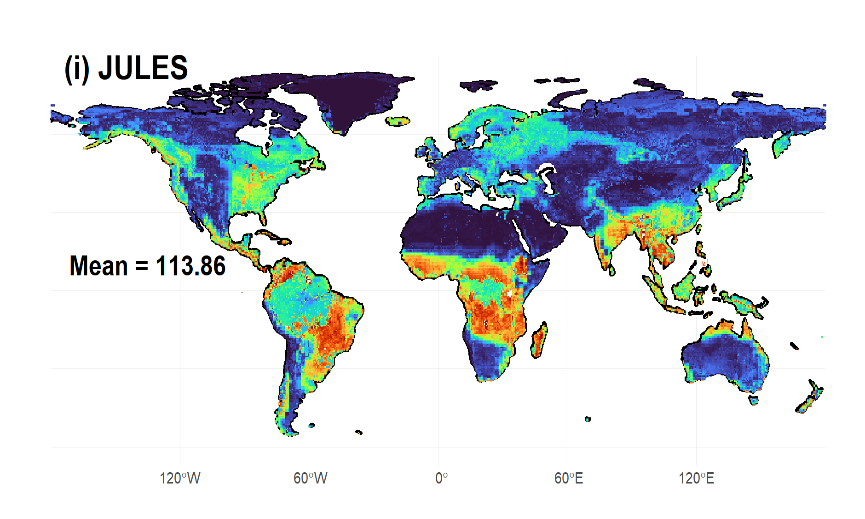

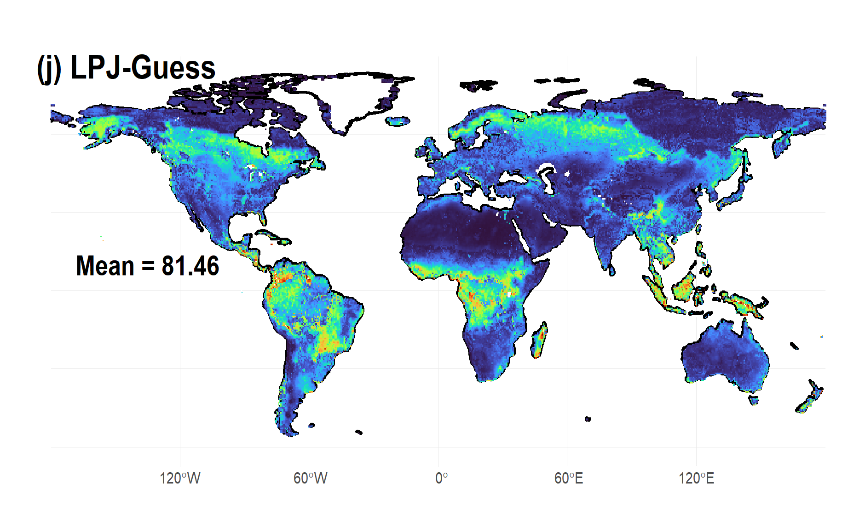


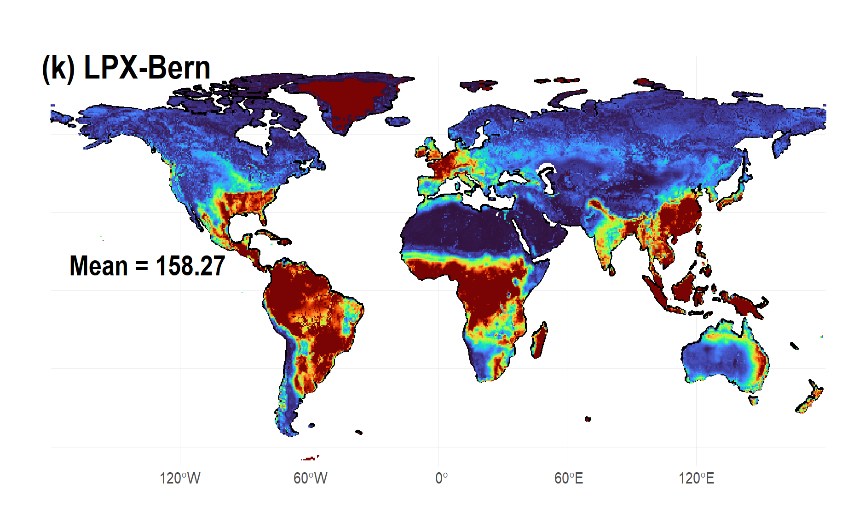

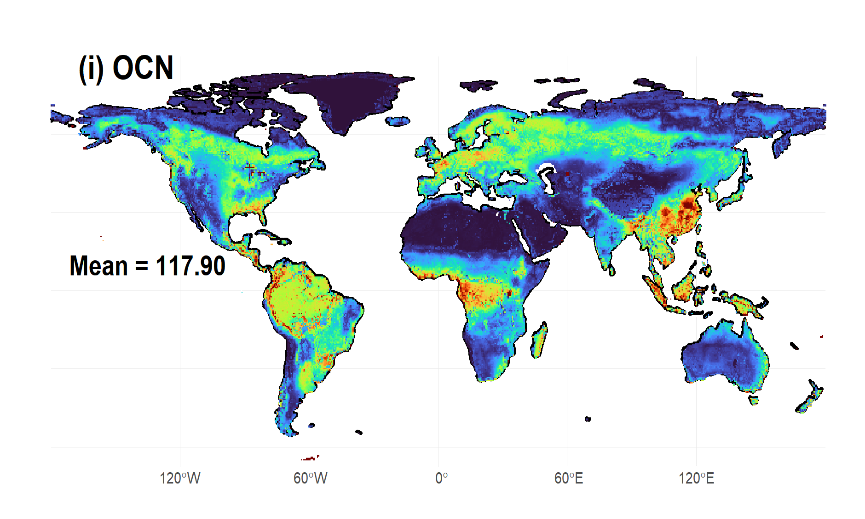


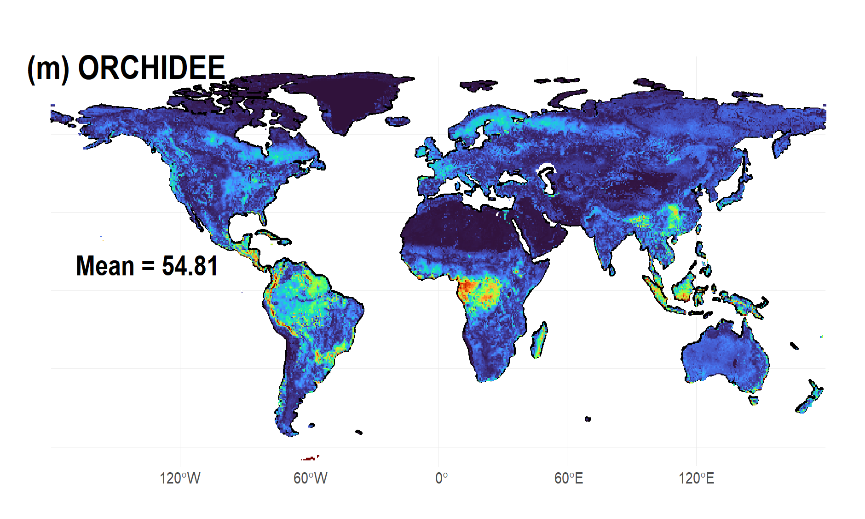

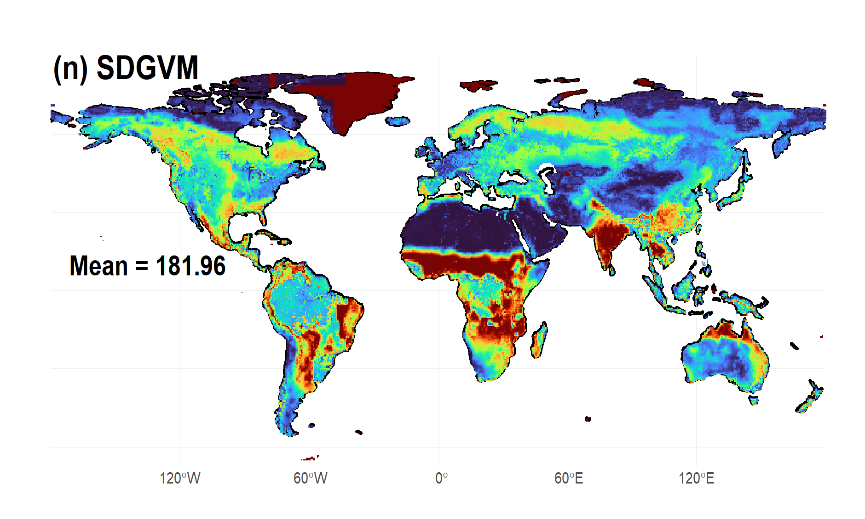


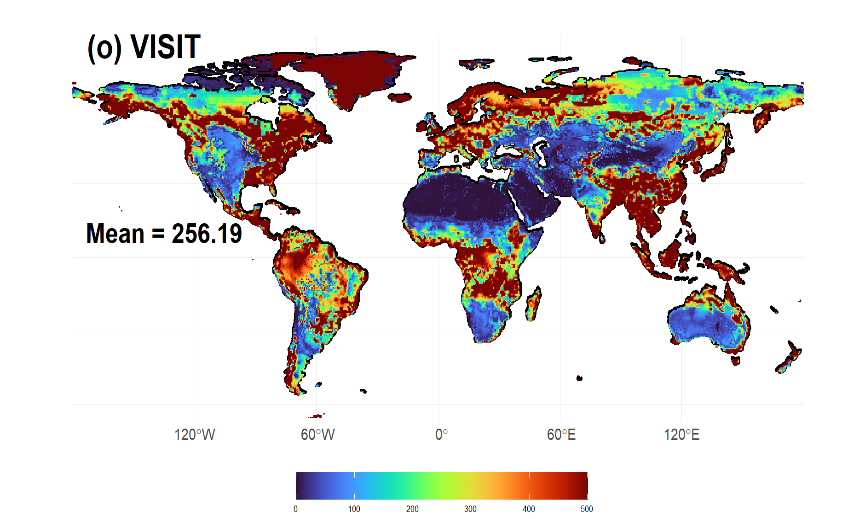

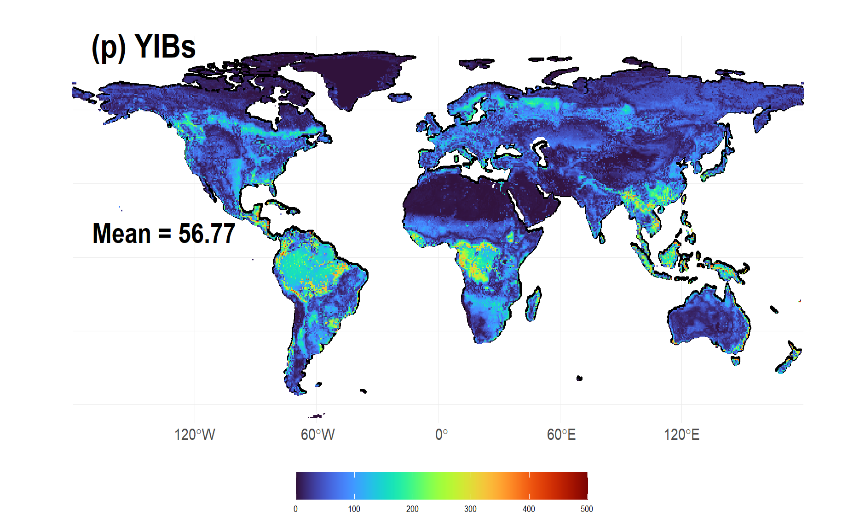


**Fig**.**S13** The spatial distribution of bias between modelled and MODIS-derived annual average LAI time series (from 2001 to 2019). (a) is the bias between our model and satellite products; (b)-(p) are the bias between other 15 models of Trendy project and satellite products. Cropland, snow/ice, and non-vegetated areas are shown in white.


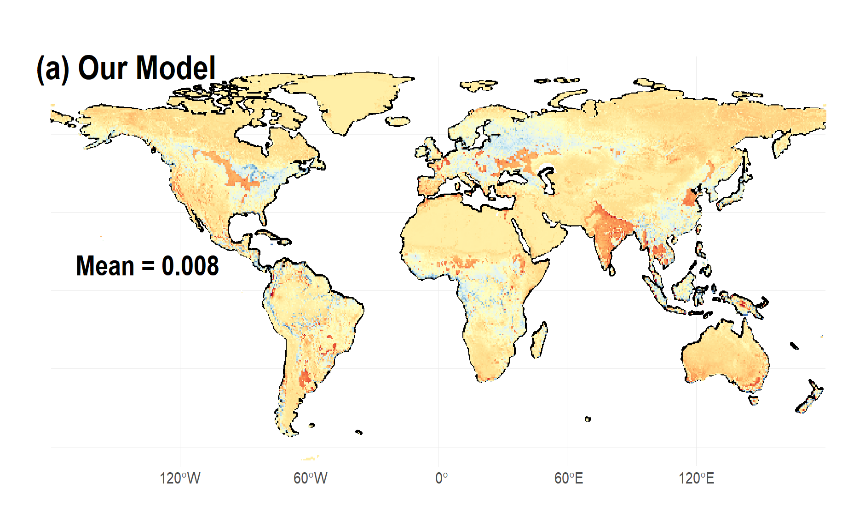

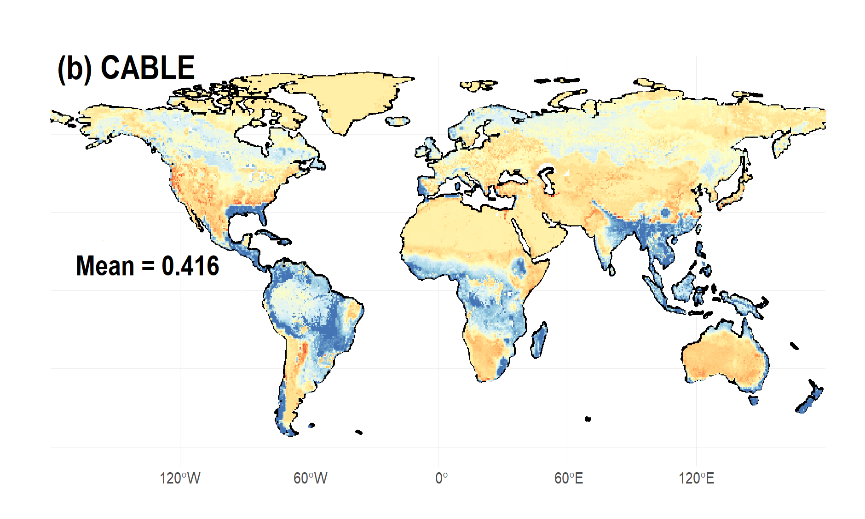


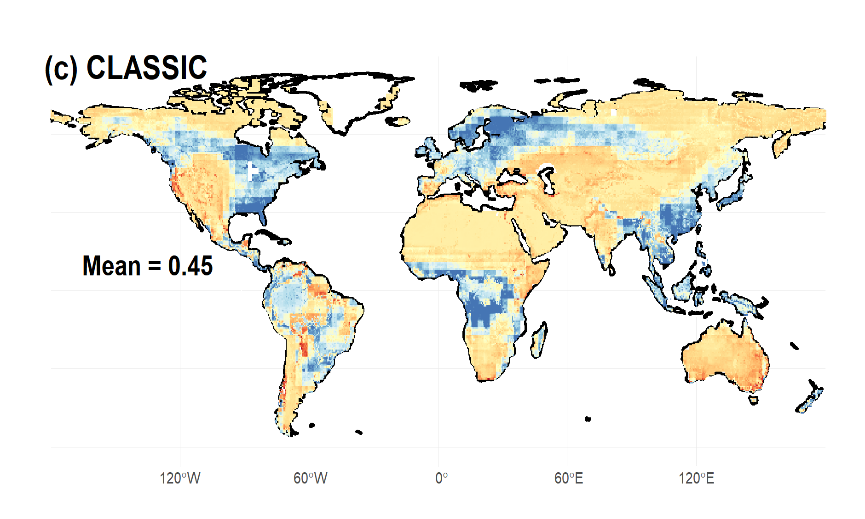

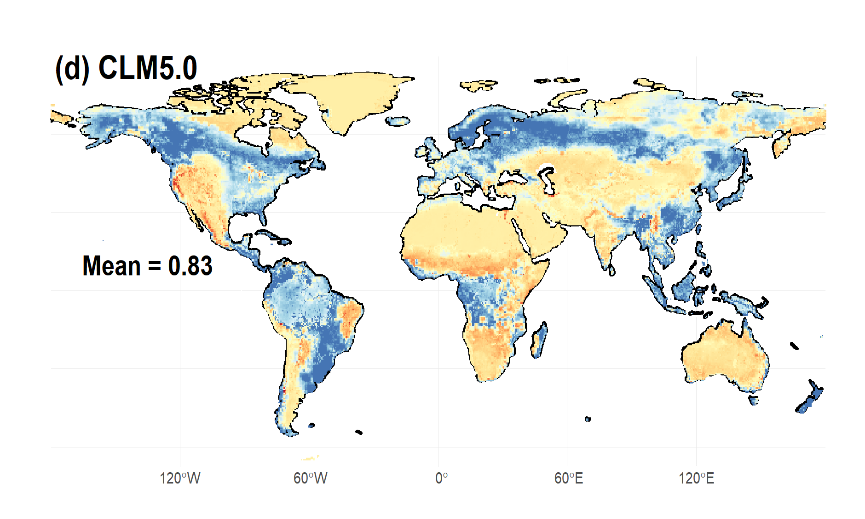


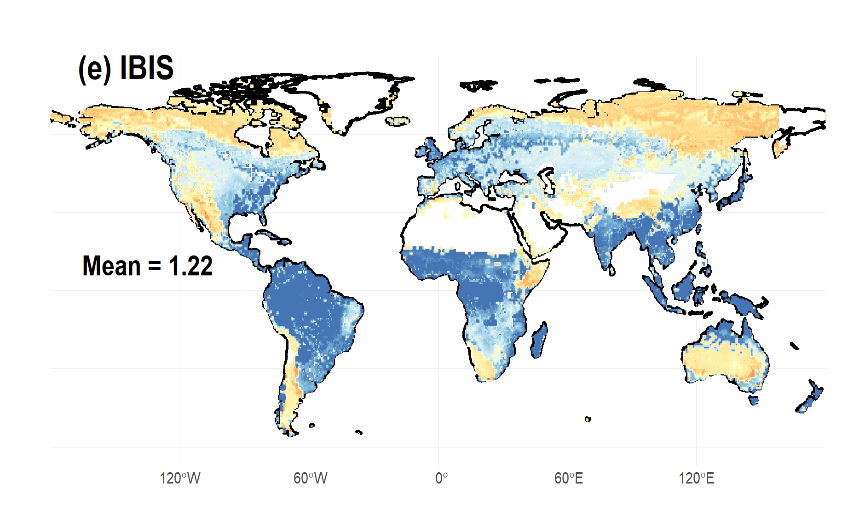

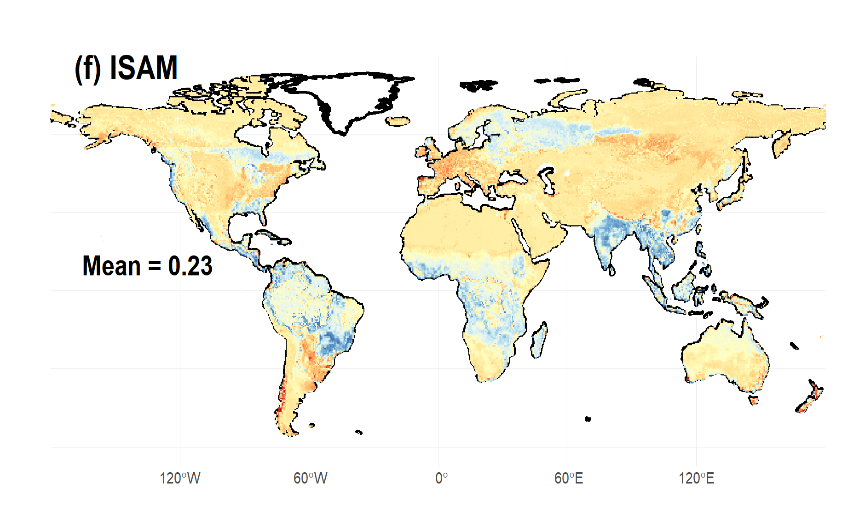


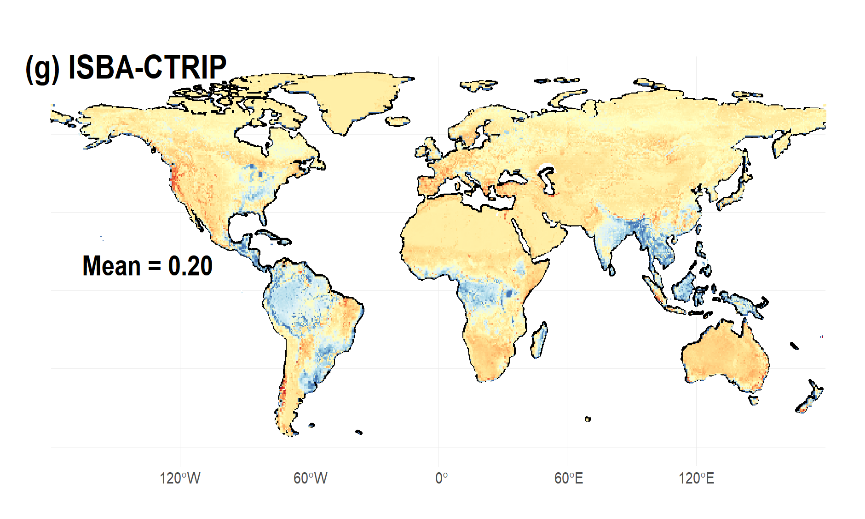

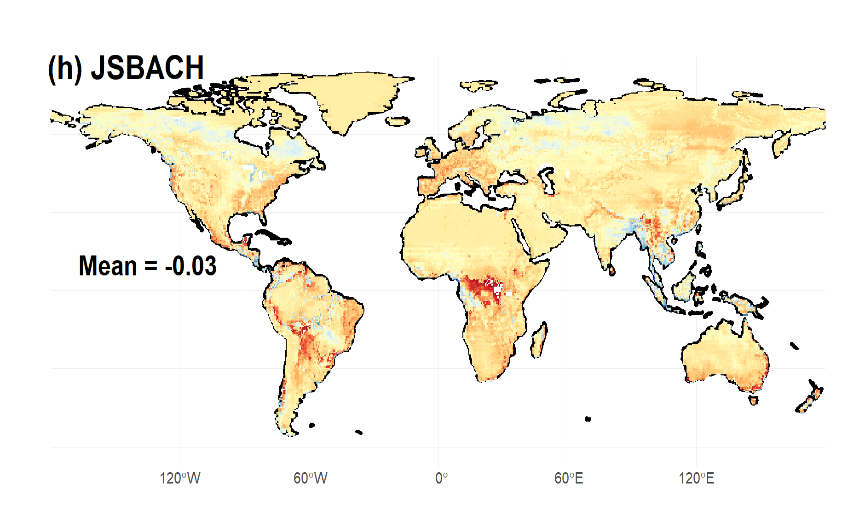


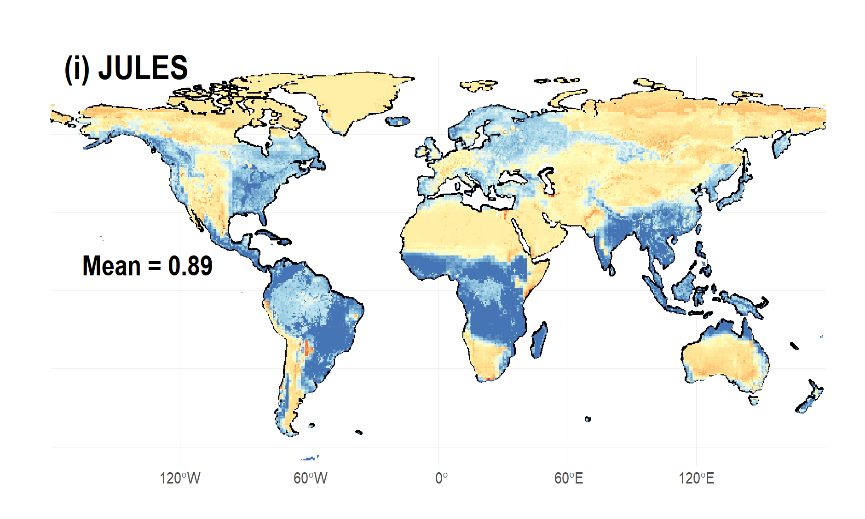

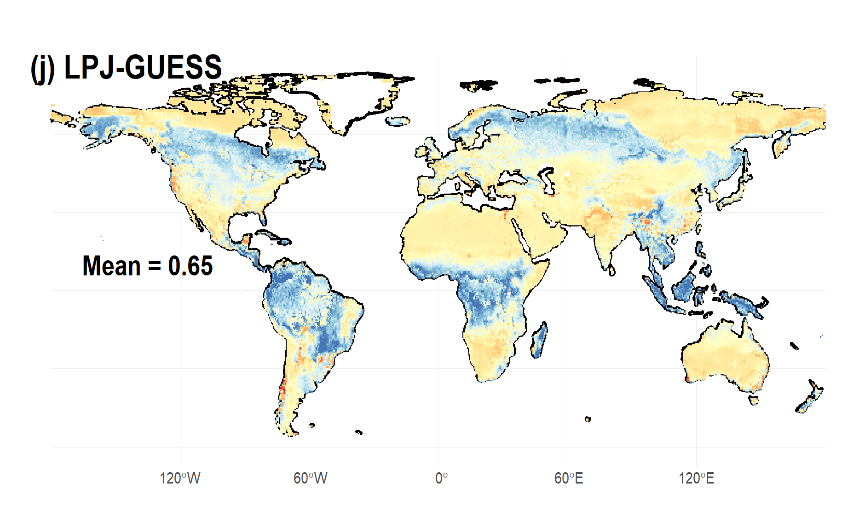


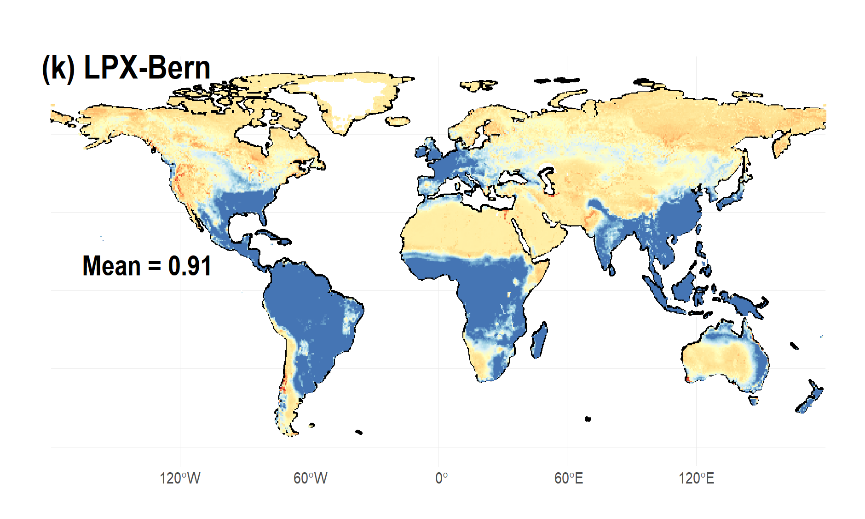

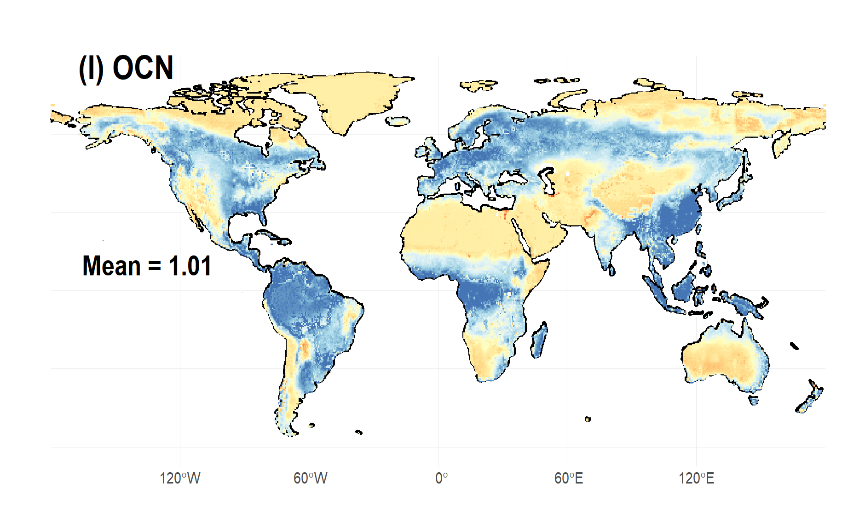


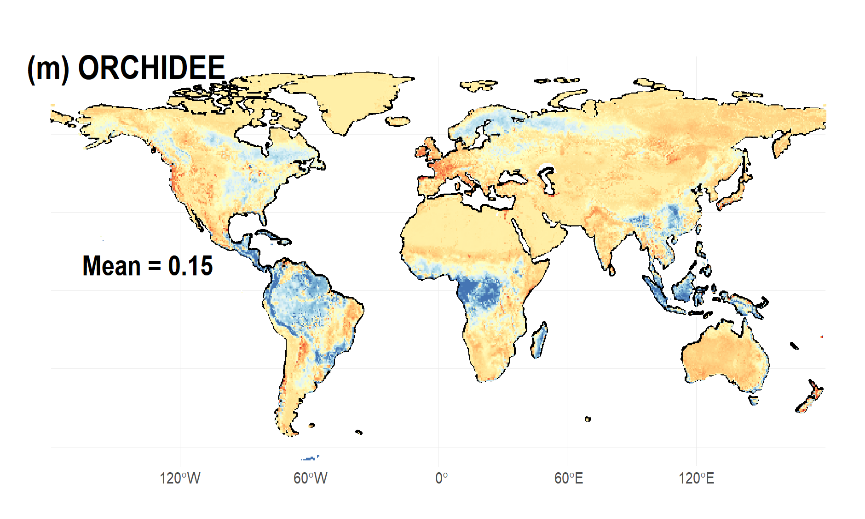

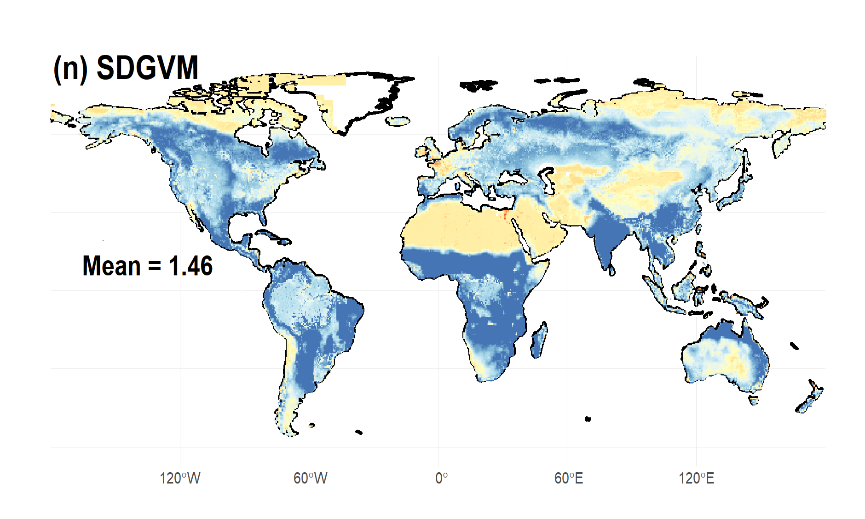


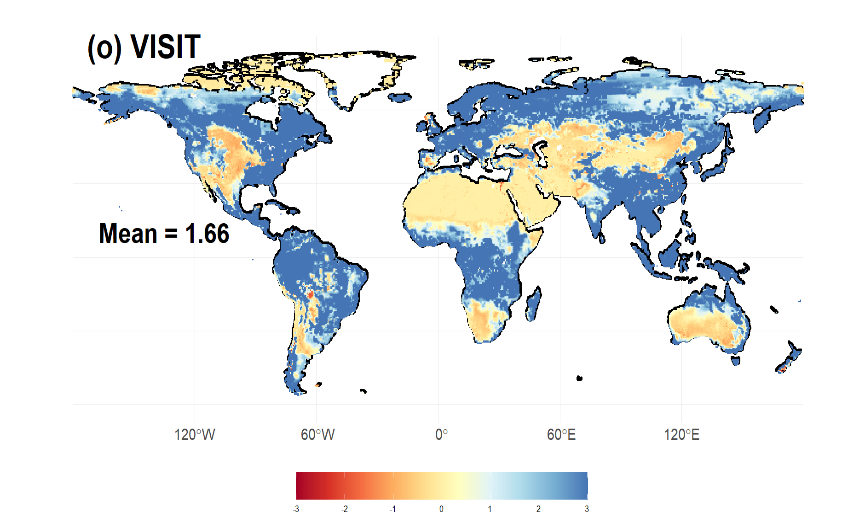

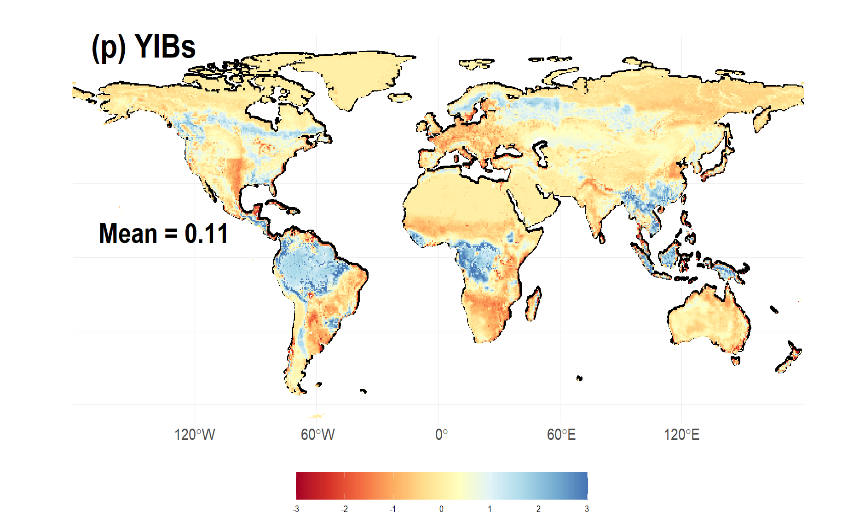


**Fig**.**S14** The spatial distribution of Pearson correlation coefficient (r) between modelled and MODIS-derived annual average LAI time series (from 2001 to 2019). (a) is the Pearson correlation coefficient between our model and satellite products; (b)-(p) are the Pearson correlation coefficient between other 15 models of Trendy project and satellite products. N = 12 and r = 0.37 correspond to the 0.100 significance level. Cropland, snow/ice, and non-vegetated areas are shown in white.


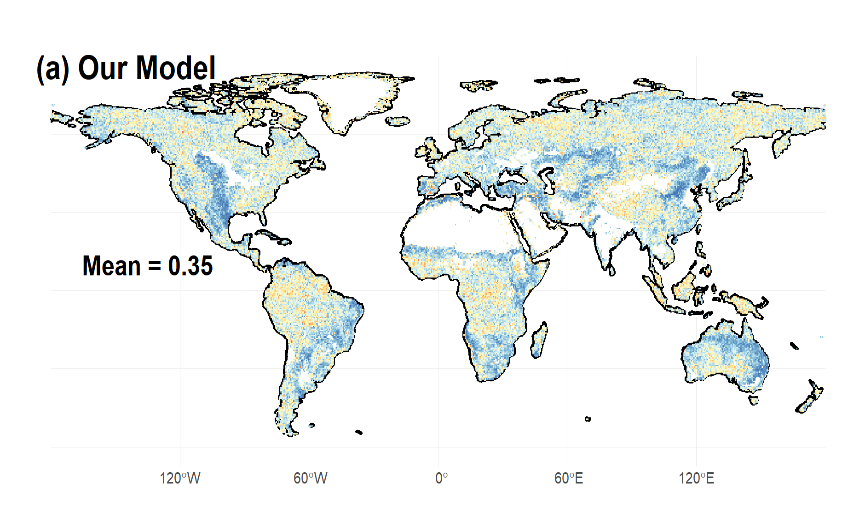

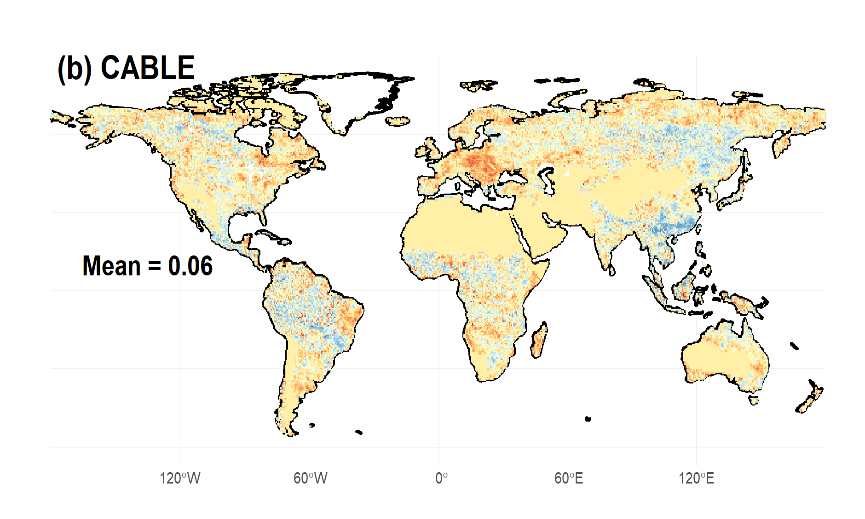


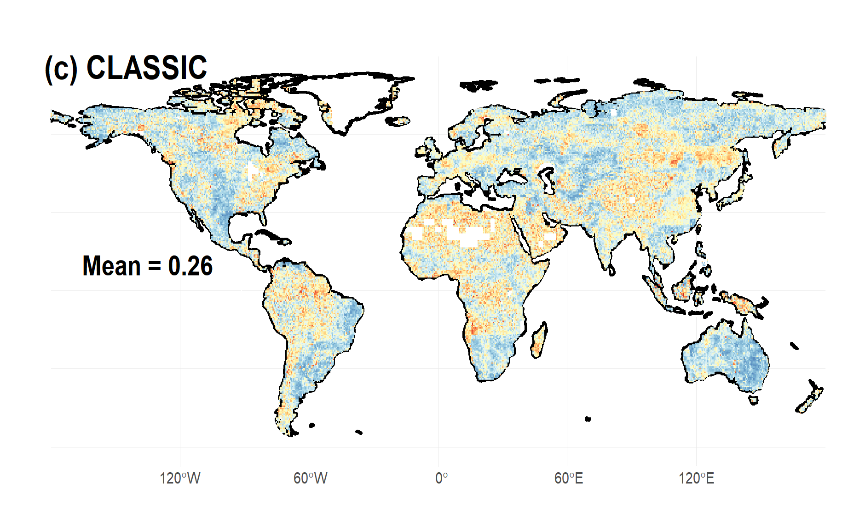

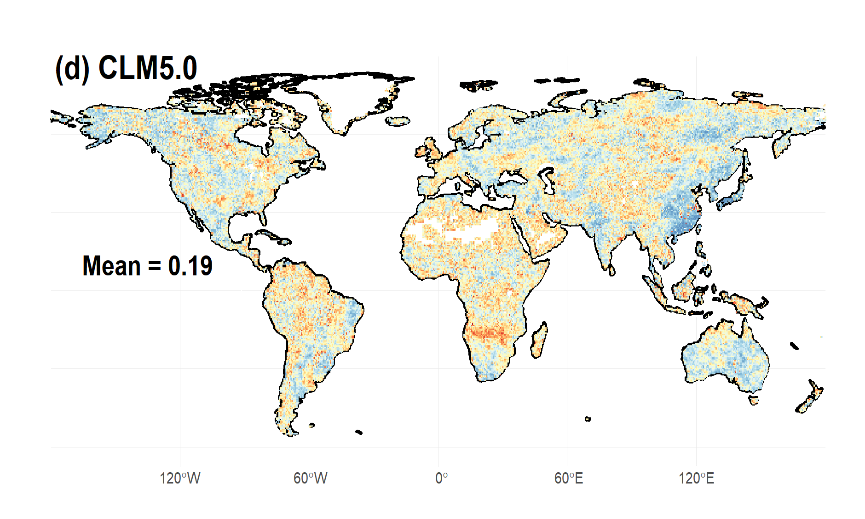


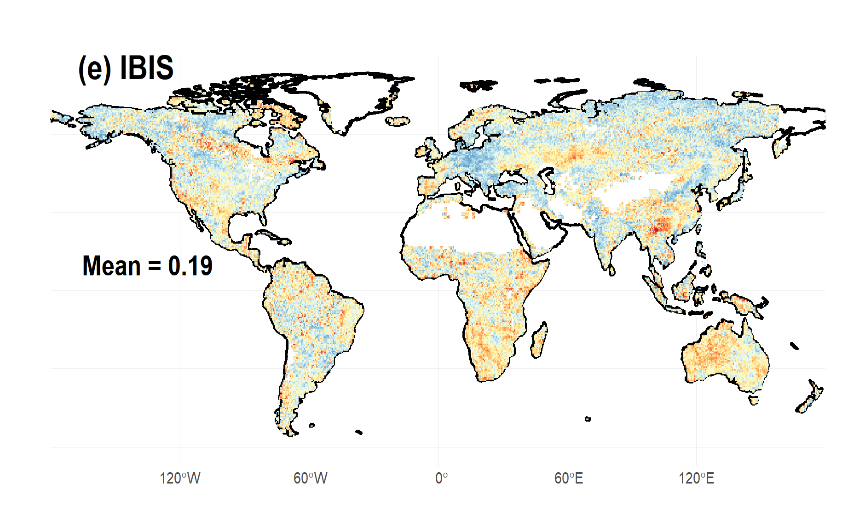

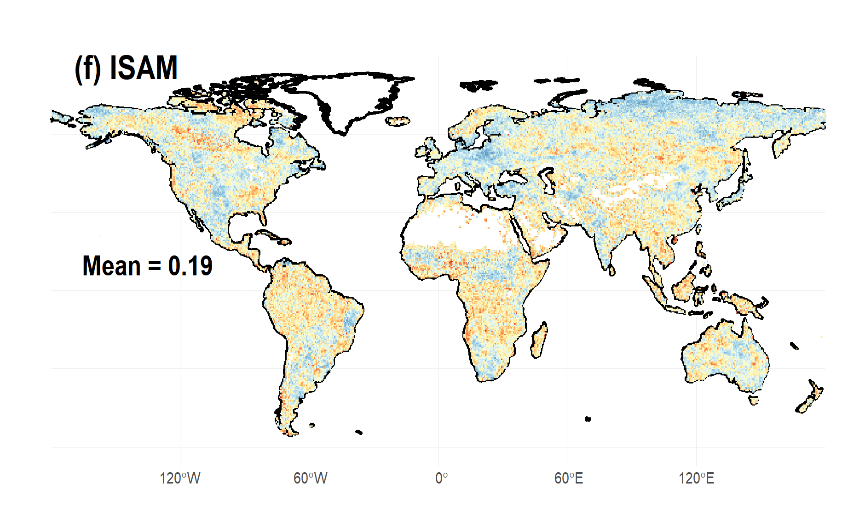


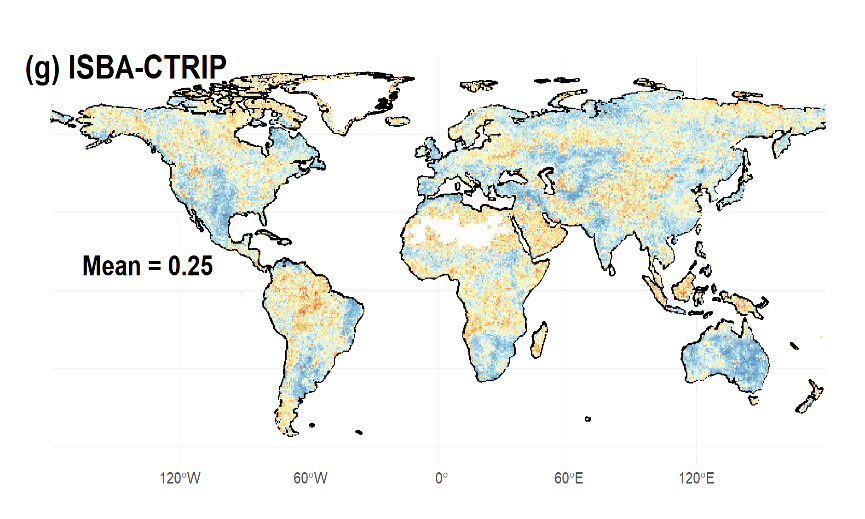

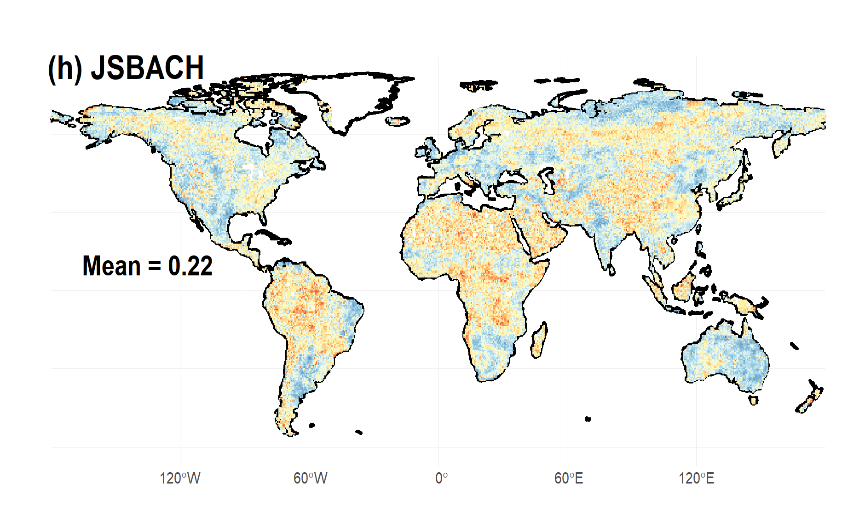


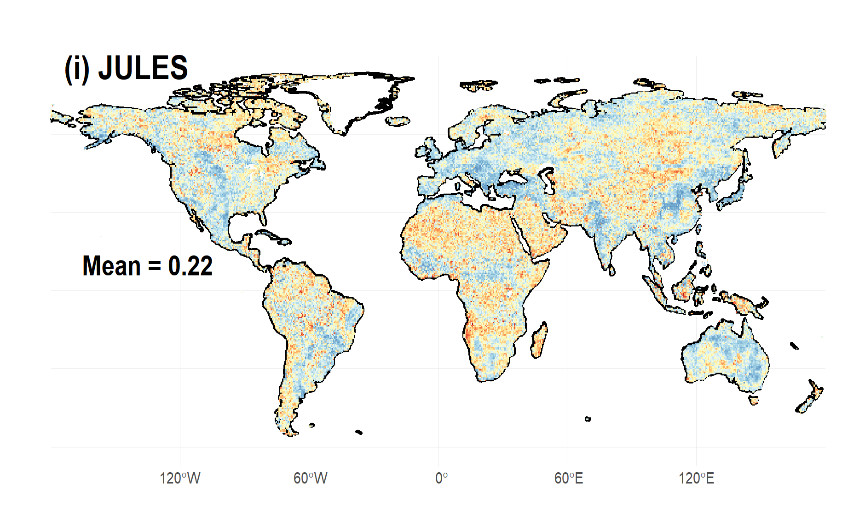

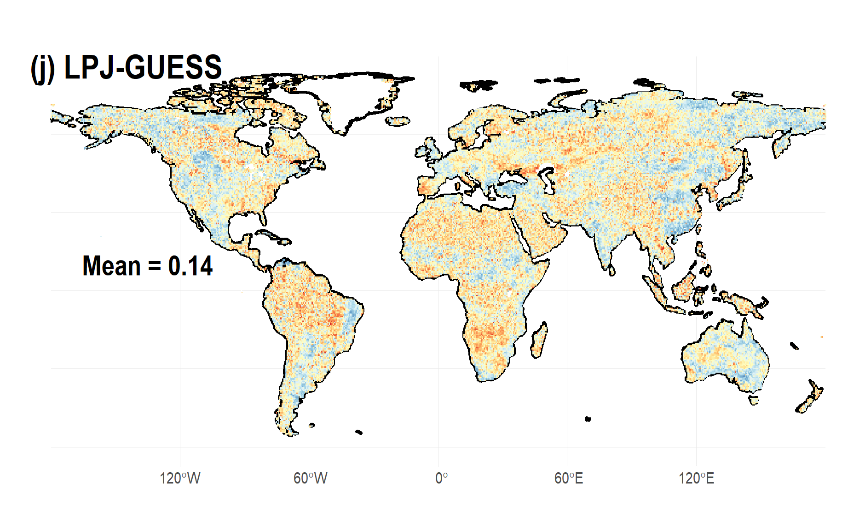


**Fig.S15** The spatial distribution of relative root mean squared error (RRMSE) between modelled and MODIS-derived monthly LAI time series in 2010. (a) is the RRMSE between our model and satellite products; (b)-(p) are the RRMSE between other 15 models of Trendy project and satellite products. Cropland, snow/ice, and non-vegetated areas are shown in white.

**Fig.S16** The spatial distribution of bias between modelled and MODIS-derived monthly LAI time series in 2010. (a) is the bias between our model and satellite products; (b)-(p) are the bias between other 15 models of Trendy project and satellite products. Cropland, snow/ice, and non-vegetated areas are shown in white.

**Fig.S17** The spatial distribution of bias between modelled and MODIS-derived monthly LAI time series in 2010. (a) is the bias between our model and satellite products; (b)-(p) are the bias between other 15 models of Trendy project and satellite products. N = 12 and r = 0.499 correspond to the 0.100 significance level. Cropland, snow/ice, and non-vegetated areas are shown in white.

**Figure S18** Differences between simulated and Copernicus-derived multi-year Leaf Area Index (LAI) mean values (2001-2019) (Simulated LAI – Copernicus-derived LAI). The insert histogram showed the distribution of differences between intervals. The blue dashed line indicates ± 0.56 intervals, while red dashed line indicates ± 1.12 intervals. Cropland, snow/ice, and non-vegetated areas are shown in white.

**Figure S19** The seasonal differences between simulated and Copernicus-derived Leaf Area Index (LAI) values (2001-2019) (Simulated LAI – Copernicus-derived LAI) on a global scale. Blue means underestimation of monthly mean LAI, red colour means overestimation of monthly mean LAI. Cropland, snow/ice, and non-vegetated areas are shown in white.

**Figure S20** Regression analysis of multi-year average of simulated LAI and observed LAI from Copernicus (2001 to 2019). (a) is the regression between our model and observed LAI from Copernicus; (b)-(p) are the regression between other 15 models of Trendy project and observed LAI from Copernicus. The red solid line is 1:1 line; the black dashed line is the regression line. RMSE: root-mean-squared error of prediction; R^2^: proportion of observed variance accounted for by the prediction.

**Figure** **S21** The spatial distribution of relative root mean squared error (RRMSE) between modelled and Copernicus-derived annual average LAI time series (from 2001 to 2019). (a) is the RRMSE between our model and satellite products; (b)-(p) are the RRMSE between other 15 models of Trendy project and satellite products. Cropland, snow/ice, and non-vegetated areas are shown in white.

**Figure** **S22** The spatial distribution of bias between modelled and Copernicus-derived annual average LAI time series (from 2001 to 2019). (a) is the bias between our model and satellite products; (b)-(p) are the bias between other 15 models of Trendy project and satellite products. Cropland, snow/ice, and non-vegetated areas are shown in white.

**Figure** **S23** The spatial distribution of Pearson correlation coefficient (r) between modelled and Copernicus-derived annual average LAI time series (from 2001 to 2019). (a) is the Pearson correlation coefficient between our model and satellite products; (b)-(p) are the Pearson correlation coefficient between other 15 models of Trendy project and satellite products. N = 12 and r = 0.37 correspond to the 0.100 significance level. Cropland, snow/ice, and non-vegetated areas are shown in white.

**Figure S24** The spatial distribution of relative root mean squared error (RRMSE) between modelled and Copernicus-derived monthly LAI time series in 2010. (a) is the RRMSE between our model and satellite products; (b)-(p) are the RRMSE between other 15 models of Trendy project and satellite products. Cropland, snow/ice, and non-vegetated areas are shown in white.

**Figure S25** The spatial distribution of bias between modelled and Copernicus-derived monthly LAI time series in 2010. (a) is the bias between our model and satellite products; (b)-(p) are the bias between other 15 models of Trendy project and satellite products. Cropland, snow/ice, and non-vegetated areas are shown in white.

**Figure S26** The spatial distribution of bias between modelled and Copernicus-derived monthly LAI time series in 2010. (a) is the bias between our model and satellite products; (b)-(p) are the bias between other 15 models of Trendy project and satellite products. N = 12 and r = 0.499 correspond to the 0.100 significance level. Cropland, snow/ice, and non-vegetated areas are shown in white.

**References**

Acosta, M., Pavelka, M., Montagnani, L., Kutsch, W., Lindroth, A., Juszczak, R., and Janouš, D.(2013) Soil surface CO_2_ efflux measurements in Norway spruce forests: Comparison between four different sites across Europe from boreal to alpine forest, *Geoderma*, **192**, 295–303, <https://doi.org/10.1016/j.geoderma.2012.08.027>.

Allison, V. J., Miller, R. M., Jastrow, J. D., Matamala, R., Zak, D. R.(2005) Changes in soil microbial community structure in a tallgrass prairie chronosequence, *Soil Science Society Of America Journal*, **69(5),** 1412-1421, 2005.

Ammann, C., Spirig, C., Leifeld, J., and Neftel, A.(2009) Assessment of the nitrogen and carbon budget of two managed temperate grassland fields, *Agr. Ecosyst. Environ*, **133**, 150–162, <https://doi.org/10.1016/j.agee.2009.05.006>.

Andrykanus, R. (2012) Howard Springs Understory_old_20131128 OzFlux: Australian and New Zealand flux research and monitoring, <https://102.100.100/14224>.

Arain, M. A. and Restrepo-Coupe, N.(2005) Net ecosystem production in a temperate pine plantation In southeastern Canada, *Agric. For. Meteorol*., **128(3-4),** 223-241, 2005.

Archibald, S. A., Kirton, A., van der Merwe, M. R., Scholes, R. J., Williams, C. A., and Hanan, N.(2009) Drivers of interannual variability in Net Ecosystem Exchange in a semiarid savanna ecosystem, South Africa, *Biogeosci*., **6,** 251–266, <https://doi.org/10.5194/bg-6-251-2009>.

Ardo, J., Molder, M., El-Tahir, B. A., and Elkhidir, H. A. M.(2008) Seasonal variation of carbon fluxes in a sparse savanna in semi-arid Sudan, *Carb. Bal. Manage*., **3**, 7, <https://doi.org/10.1186/1750-0680-3-7>.

Aubinet, M., Chermanne, B., Vandenhaute, M., Longdoz, B., Yernaux, M., and Laitat, E.(2001) Long term carbon dioxide exchange above a mixed forest in the Belgian Ardennes, *Agr. Forest Meteorol*., **108**, 293–315, <https://doi.org/10.1016/s0168-1923(01)00244-1>.

Baldocchi, D., Chen, Q., Chen, X., Ma, S., Miller, G., Ryu, Y., Xiao, J., Wenk, R., and Battles, J.(2010) The dynamics of energy, water, and carbon fluxes in a blue oak (Quercus douglasii) savanna in California, in: ecosystem function in savannas, **135–151**, CR Press, <https://doi.org/10.1201/b10275-10>.

Belelli Marchesini, L., Papale, D., Reichstein, M., Vuichard, N., Tchebakova, N., and Valentini, R.(2007) Carbon balance assessment of a natural steppe of southern Siberia by multiple constraint approach, *Biogeosciences*, **4,** 581–595, <https://doi.org/10.5194/bg4-581-2007>.

Berbigier, P., Bonnefond, J.-M., and Mellmann, P.(2001) CO_2_ and water vapour fluxes for 2 years above Euroflux forest site, *Agr Forest Meteorol*., **108**, 183–197, <https://doi.org/10.1016/s0168-1923(01)00240-4>.

Bergeron, O., Margolis, H. A., Black, T. A., Coursolle, C., Dunn, A. L., Barr, A. G., and Wofsy, S. C.(2007) Comparison of carbon dioxide fluxes over three boreal black spruce forests in Canada, *Global Change Biol.*, **13**, 89–107, <https://doi.org/10.1111/j.1365-2486.2006.01281.x>.

Beringer, J., Hacker, J., Hutley, L. B., Leuning, R., Arndt,S. K., Amiri, R., Bannehr, L., Cernusak, L. A., Grover, S., Hensley, C., Hocking, D., Isaac, P., Jamali, H., Kanniah, K., Livesley, S., Neininger, B., U, K. T. P., Sea, W., Straten, D., Tapper, N., Weinmann, R., Wood, S., and Zegelin, S.(2011a) SPECIAL–Savanna patterns of energy and carbon integrated across the landscape, *B. Am. Meteorol. Soc*., **92**, 1467–1485, <https://doi.org/10.1175/2011bams2948.1>.

Beringer, J., Hutley, L. B., Hacker, J. M., Neininger, B., and U, K. T. P.(2011b) Patterns and processes of carbon, water and energy cycles across northern Australian landscapes: From point to region, *Agr. Forest Meteorol.*, **151**, 1409–1416, <https://doi.org/10.1016/j.agrformet.2011.05.003>.

Beringer, J., Hutley, L. B., Hacker, J. M., Neininger, B., and U, K. T. P.(2011c) Patterns and processes of carbon, water and energy cycles across northern Australian landscapes: From point to region, *Agr. Forest Meteorol*., **151**, 1409–1416, <https://doi.org/10.1016/j.agrformet.2011.05.003>.

Beringer, J., Hutley, L. B., McHugh, I., Arndt, S. K., Campbell, D., Cleugh, H. A., Cleverly, J., Resco de Dios, V., Eamus, D., Evans, B., Ewenz, C., Grace, P., Griebel, A., Haverd, V., HinkoNajera, N., Huete, A., Isaac, P., Kanniah, K., Leuning, R., Liddell, M. J., Macfarlane, C., Meyer, W., Moore, C., Pendall, E., Phillips, A., Phillips, R. L., Prober, S. M., Restrepo-Coupe, N., Rutledge, S., Schroder, I., Silberstein, R., Southall, P., Yee, M.S., Tapper, N. J., van Gorsel, E., Vote, C., Walker, J., and Wardlaw, T.(2016) An introduction to the Australian and New Zealand flux tower network – OzFlux, *Biogeosciences,* **13**, 5895–5916, <https://doi.org/10.5194/bg-13-5895-2016>.

Bonal, D., Bosc, A., Ponton, S., Goret, J.-Y., Burban, B., Gross,P., Bonnefond, J.-M., Elbers, J., Longdoz, B., Epron, D., Guehl, J.-M., and Granier, A.(2008) Impact of severe dry season on net ecosystem exchange in the Neotropical rainforest of French Guiana, *Global Change Biol*., **14**, 1917–1933, <https://doi.org/10.1111/j.1365-2486.2008.01610.x>.

Bowling, D. R., Bethers-Marchetti, S., Lunch, C. K., Grote, E. E., and Belnap, J.(2010) Carbon, water, and energy fluxes in a semiarid cold desert grassland during and following multiyear drought, *J. Geophys. Res*., **115**, G4, <https://doi.org/10.1029/2010jg001322>.

Bristow, M., Hutley, L. B., Beringer, J., Livesley, S. J., Edwards, A. C., and Arndt, S. K.(2016) Quantifying the relative importance of greenhouse gas emissions from current and future savanna land use change across northern Australia, *Biogeosciences*, **13**, 6285– 6303, <https://doi.org/10.5194/bg-13-6285-2016>.

Cai, W., Zhu, Z.Q., Harrison, S.P., Ryu, Y., Wang, H., Zhou, B.Y., and Prentice, I.C. (2024). A unifying principle for global greenness patterns and trends. BioRXiv doi: https://doi.org/10.1101/2023.02.25.529932

Cernusak, L. A., Hutley, L. B., Beringer, J., Holtum, J. A.,and Turner, B. L.(2011) Photosynthetic physiology of eucalypts along a sub-continental rainfall gradient in northern Australia, *Agr. Forest Meteorol*., **151**, 1462–1470, <https://doi.org/10.1016/j.agrformet.2011.01.006>.

Chen, S., Chen, J., Lin, G., Zhang, W., Miao, H., Wei, L., Huang, J., and Han, X.(2009) Energy balance and partition in Inner Mongolia steppe ecosystems with different land use types, *Agr. Forest Meteorol*., **149**, 1800–1809, <https://doi.org/10.1016/j.agrformet.2009.06.009>.

Chiesi, M., Maselli, F., Bindi, M., Fibbi, L., Cherubini, P., Arlotta, E., Tirone, G., Matteucci, G., and Seufert, G.(2005) Modelling carbon budget of Mediterranean forests using ground and remote sensing measurements, *Agr. Forest Meteorol*., **135**, 22–34, <https://doi.org/10.1016/j.agrformet.2005.09.011>.

Cleverly, J., Boulain, N., Villalobos-Vega, R., Grant, N., Faux, R., Wood, C., Cook, P. G., Yu, Q., Leigh, A., and Eamus, D.(2013) Dynamics of component carbon fluxes in a semi-arid Acacia woodland, central Australia, *J. Geophys. Res.-Biogeosci*., **118**, 1168–1185, <https://doi.org/10.1002/jgrg.20101>.

Cleverly, J., Eamus, D., Van Gorsel, E., Chen, C., Rumman, R., Luo, Q., Coupe, N. R., Li, L., Kljun, N., Faux, R., Yu, Q., and Huete, A.(2016) Productivity and evapotranspiration of two contrasting semiarid ecosystems following the 2011 global carbon land sink anomaly, *Agr. Forest Meteorol*., **220**, 151–159, <https://doi.org/10.1016/j.agrformet.2016.01.086>.

Cook, B. D., Davis, K. J., Wang, W., Desai, A., Berger, B. W., Teclaw, R. M., Martin, J. G., Bolstad, P. V., Bakwin, P. S., Yi, C., and Heilman, W.(2004) Carbon exchange and venting anomalies in an upland deciduous forest in northern Wisconsin, USA, *Agr. Forest Meteorol*., **126**, 271–295, <https://doi.org/10.1016/j.agrformet.2004.06.008>.

Davis, T.W., Prentice, I.C., Stocker, B.D., Thomas, R.T., Whitley, R.J., Wang, H., Evans, B.J., Gallego-Sala, A.V., Sykes, M.T., and Cramer, W.(2017) Simple process-led algorithms for simulating habitats (SPLASH v.1.0): robust indices of radiation, evapotranspiration and plant-available moisture, *Geosci. Model Develop*., **10**, 689–708. Zenodo. <https://doi.org/10.5281/zenodo.376293>.

Delpierre, N., Berveiller, D., Granda, E., and Dufrêne, E.(2015) Wood phenology, not carbon input, controls the interannual variability of wood growth in a temperate oak forest, *New Phytol*., **210**, 459–470, <https://doi.org/10.1111/nph.13771>.

Desai, A. R., Bolstad, P. V., Cook, B. D., Davis, K. J., and Carey, E. V.(2005) Comparing net ecosystem exchange of carbon dioxide between an old-growth and mature forest in the upper Midwest, USA*, Agr. Forest Meteorol*., **128**, 33–55, <https://doi.org/10.1016/j.agrformet.2004.09.005>.

Desai, A. R., Xu, K., Tian, H., Weishampel, P., Thom, J., Baumann, D., Andrews, A. E., Cook, B. D., King, J. Y., and Kolka, R.(2015) Landscape-level terrestrial methane flux observed from a very tall tower, *Agr. Forest Meteorol*., **201**, 61–75, <https://doi.org/10.1016/j.agrformet.2014.10.017>.

Dragoni, D., Schmid, H. P., Wayson, C. A., Potter, H., Grimmond, C. S. B., and Randolph, J. C.(2011) Evidence of increased net ecosystem productivity associated with a longer vegetated season in a deciduous forest in south-central Indiana, USA, *Global Change Biol*., **17**, 886–897, <https://doi.org/10.1111/j.1365-2486.2010.02281.x>.

Dunn, A. L., Barford, C. C., Wofsy, S. C., Goulden, M. L., and Daube, B. C.(2007) A long-term record of carbon exchange in a boreal black spruce forest: means, responses to interannual variability, and decadal trends, *Global Change Biol.*, **13**, 577–590, <https://doi.org/10.1111/j.1365-2486.2006.01221.x>.

Etzold, S., Ruehr, N. K., Zweifel, R., Dobbertin, M., Zingg, A., Pluess, P., Häsler, R., Eugster, W., and Buchmann, N.(2011) The carbon balance of two contrasting mountain forest ecosystems in switzerland: similar annual trends, but seasonal differences, *Ecosystems*, **14**, 1289–1309, https://doi.org/10.1007/s10021-011-9481-3, 2011.

Fares, S., Savi, F., Muller, J., Matteucci, G., and Paoletti, E.(2014) Simultaneous measurements of above and below canopy ozone fluxes help partitioning ozone deposition between its various sinks in a Mediterranean Oak *Forest, Agr. Forest Meteorol*., **198-199**, 181–191, <https://doi.org/10.1016/j.agrformet.2014.08.014>.

Frank, J. M., Massman, W. J., Ewers, B. E., Huckaby, L. S., and Negrón, J. F.(2014) Ecosystem CO_2_/H_2_O fluxes are explained by hydraulically limited gas exchange during tree mortality from spruce bark beetles, *J. Geophys. Res.-Biogeosci*., **119**, 1195–1215, <https://doi.org/10.1002/2013jg002597>.

Galvagno, M., Wohlfahrt, G., Cremonese, E., Rossini, M., Colombo, R., Filippa, G., Julitta, T., Manca, G., Siniscalco, C.,di Cella, U. M., and Migliavacca, M.(2013) Phenology and carbon dioxide source/sink strength of a subalpine grassland in response to an exceptionally short snow season, *Environ. Res. Lett.*, **8**, 025008, <https://doi.org/10.1088/1748-9326/8/2/025008>.

Good, S.P., Moore, G.W., Miralles, D.G.(2017) A mesic maximum in biological water use demarcates biome sensitivity to aridity shifts. *Nat Ecol Evol.* **1,** 1883–1888. <https://doi.org/10.1038/s41559-017-0371-8>.

Gough, C. M., Hardiman, B. S., Nave, L. E., Bohrer, G., Maurer, K. D., Vogel, C. S., Nadelhoffer, K. J., and Curtis, P. S.(2013) Sustained carbon uptake and storage following moderate disturbance in a Great Lakes forest, *Ecol. Appl*., **23**, 1202–1215, <https://doi.org/10.1890/12-1554.1>.

Grünwald, T. and Bernhofer, C.(2007) A decade of carbon, water and energy flux measurements of an old spruce forest at the Anchor Station Tharandt, Tellus B, **59**, 387–396, <https://doi.org/10.3402/tellusb.v59i3.17000>.

Guan, D.-X., Wu, J.-B., Zhao, X.-S., Han, S.-J., Yu, G.-R., Sun,X.-M., and Jin, C.-J.(2006) CO_2_ fluxes over an old, temperate mixed forest in northeastern China, *Agr. Forest Meteorol*., **137**, 138–149, <https://doi.org/10.1016/j.agrformet.2006.02.003>.

Hinko-Najera, N., Isaac, P., Beringer, J., van Gorsel, E., Ewenz, C., McHugh, I., Exbrayat, J.-F., Livesley, S. J., and Arndt, S. K.(2017) Net ecosystem carbon exchange of a dry temperate eucalypt forest, *Biogeosciences*, **14**, 3781–3800, <https://doi.org/10.5194/bg-14-3781-2017>.

Hutley, L. B., Beringer, J., Isaac, P. R., Hacker, J. M., and Cernusak, L. A.(2011) A sub-continental scale living laboratory: Spatial patterns of savanna vegetation over a rainfall gradient in northern Australia, *Agr. Forest Meteorol*., **151**, 1417–1428, <https://doi.org/10.1016/j.agrformet.2011.03.002>.

Imer, D., Merbold, L., Eugster, W., and Buchmann, N.(2013) Temporal and spatial variations of soil CO_2_, CH_4_ and N_2_O fluxes at three differently managed grasslands, *Biogeosciences*, **10,** 5931–5945, <https://doi.org/10.5194/bg-10-5931-2013>.

Irvine, J., Law, B. E., and Hibbard, K. A.(2007) Postfire carbon pools and fluxes in semiarid ponderosa pine in Central Oregon, *Global Change Biol*., **13**, 1748–1760, <https://doi.org/10.1111/j.1365-> 2486.2007.01368.x, 2007.

Jacobs, C. M. J., Jacobs, A. F. G., Bosveld, F. C., Hendriks, D.M. D., Hensen, A., Kroon, P. S., Moors, E. J., Nol, L., SchrierUijl, A., and Veenendaal, E. M.(2007) Variability of annual CO_2_ exchange from Dutch grasslands, *Biogeosciences*, **4,** 803–816, <https://doi.org/10.5194/bg-4-803-2007>.

Kato, T., Tang, Y., Gu, S., Hirota, M., Du, M., Li, Y., and Zhao, X.(2006) Temperature and biomass influences on interannual changes in CO_2_ exchange in an alpine meadow on the Qinghai-Tibetan Plateau, *Global Change Biol*., **12,** 1285–1298, <https://doi.org/10.1111/j.1365-2486.2006.01153.x>.

Kilinc, M., Beringer, J., Hutley, L. B., Tapper, N. J., and McGuire, D. A.(2013) Carbon and water exchange of the world’s tallest angiosperm forest, *Agr. Forest Meteorol*., **182–183**, 215–224, <https://doi.org/10.1016/j.agrformet.2013.07.003>.

Knohl, A., Schulze, E.-D., Kolle, O., and Buchmann, N.(2003) Large carbon uptake by an unmanaged 250-year-old deciduous forest in Central Germany, *Agr. Forest Meteorol*., **118**, 151–167, <https://doi.org/10.1016/s0168-1923(03)00115-1>.

Kurbatova, J., Li, C., Varlagin, A., Xiao, X., and Vygodskaya, N.(2008) Modeling carbon dynamics in two adjacent spruce forests with different soil conditions in Russia, *Biogeosciences*, **5**, 969–980, <https://doi.org/10.5194/bg-5-969-2008>.

Leuning, R.(1995) A critical appraisal of a combined stomatal photosynthesis model for C3 plants, *Plant Cell Environ*., **18**, 339–355.

Lindauer, M., Schmid, H., Grote, R., Mauder, M., Steinbrecher, R., and Wolpert, B.(2014) Net ecosystem exchange over a non-cleared wind-throw-disturbed upland spruce forest–Measurements and simulations, *Agr. Forest Meteorol*., **197**, 219–234, <https://doi.org/10.1016/j.agrformet.2014.07.005>.

Ma, S., Baldocchi, D. D., Xu, L., and Hehn, T.(2007) Inter-annual variability in carbon dioxide exchange of an oak/grass savanna and open grassland in California, *Agr. Forest Meteorol*., **147**, 157–171, <https://doi.org/10.1016/j.agrformet.2007.07.008>.

Marcolla, B., Cescatti, A., Manca, G., Zorer, R., Cavagna, M., Fiora, A., Gianelle, D., Rodeghiero, M., Sottocornola, M., and Zampedri, R.(2011) Climatic controls and ecosystem responses drive the inter-annual variability of the net ecosystem exchange of an alpine meadow, *Agr. Forest Meteorol*., **151**, 1233–1243, <https://doi.org/10.1016/j.agrformet.2011.04.015>.

Marcolla, B., Pitacco, A., and Cescatti, A.(2003) Canopy architecture and turbulence structure in a coniferous forest, bound.-layer *Meteorol.,* **108**, 39–59, <https://doi.org/10.1023/a:1023027709805>.

Matsumoto, K., Ohta, T., Nakai, T., Kuwada, T., Daikoku, K., Iida, S., Yabuki, H., Kononov, A. V., van der Molen, M. K., Kodama,Y., Maximov, T. C., Dolman, A. J., and Hattori, S.(2008) Energy consumption and evapotranspiration at several boreal and temperate forests in the Far East, *Agr. Forest Meteorol.*, **148**, 1978–1989, <https://doi.org/10.1016/j.agrformet.2008.09.008>.

McHugh, I. D., Beringer, J., Cunningham, S. C., Baker, P. J., Cavagnaro, T. R., Mac Nally, R., and Thompson, R. M.(2017) Interactions between nocturnal turbulent flux, storage and advection at an “deal” eucalypt woodland site, *Biogeosciences*, **14**, 3027–3050, <https://doi.org/10.5194/bg-14-3027-2017>.

Mengoli, G., Agustí-Panareda, A., Boussetta, S., Harrison, S.P., Trotta, C., and Prentice, I.C.(2022) Ecosystem photosynthesis in land-surface models: a first-principles approach incorporating acclimation. *Journal of Advances in Modeling Earth Systems*., **14**, e2021MS002767, <https://doi.org/10.1029/2021MS002767>.

Merbold, L., Ardö, J., Arneth, A., Scholes, R. J., Nouvellon, Y., de Grandcourt, A., Archibald, S., Bonnefond, J. M., Boulain, N., Brueggemann, N., Bruemmer, C., Cappelaere, B., Ceschia, E., El-Khidir, H. A. M., El-Tahir, B. A., Falk, U., Lloyd, J., Kergoat, L., Le Dantec, V., Mougin, E., Muchinda, M., Mukelabai, M. M., Ramier, D., Roupsard, O., Timouk, F., Veenendaal, E. M., and Kutsch, W. L.(2009) Precipitation as driver of carbon fluxes in 11 African ecosystems, *Biogeosciences,* **6,** 1027–1041, <https://doi.org/10.5194/bg-6-1027-2009>.

Merbold, L., Eugster, W., Stieger, J., Zahniser, M., Nelson, D., and Buchmann, N.(2014) Greenhouse gas budget (CO_2_, CH_4_ and N_2_O) of intensively managed grassland following restoration, *Global Change Biol*., **20**, 1913–1928, <https://doi.org/10.1111/gcb.12518>.

Meyer, W. S., Kondrlovà, E., and Koerber, G. R.(2015) Evaporation of perennial semi-arid woodland in southeastern Australia is adapted for irregular but common dry periods, *Hydrol. Process*., **29**, 3714–3726, <https://doi.org/10.1002/hyp.10467>.

Migliavacca, M., Meroni, M., Busetto, L., Colombo, R., Zenone, T., Matteucci, G., Manca, G., and Seufert, G.(2009) Modelling gross primary production of Agro-Forestry ecosystems by assimilation of satellite-derived information in a process-based model, sensors, **9**, 922–942, <https://doi.org/10.3390/s90200922>.

Monson, R. K., Turnipseed, A. A., Sparks, J. P., Harley, P. C., ScottDenton, L. E., Sparks, K., and Huxman, T. E.(2002) Carbon sequestration in a high-elevation, subalpine forest, *Global Change Biol*.,**8**, 459–478, <https://doi.org/10.1046/j.1365-2486.2002.00480.x>.

Montagnani, L., Manca, G., Canepa, E., Georgieva, E., Acosta, M., Feigenwinter, C., Janous, D., Kerschbaumer, G., Lindroth, A., Minach, L., Minerbi, S., Mölder, M., Pavelka, M., Seufert, G., Zeri, M., and Ziegler, W.(2009) A new mass conservation approach to the study of CO_2_ advection in an alpine forest, *J. Geophys.* *Res*., **114**, D07306, <https://doi.org/10.1029/2008jd010650>.

Moors, E.(2012) Water use of forests in the Netherlands, Ph.D. thesis, Vrije Universiteit Amsterdam.

Noormets, A., Chen, J., and Crow, T. R.(2007) Age-dependent changes in ecosystem carbon fluxes in managed forests in northern wisconsin, USA, *Ecosystems*, **10**, 187–203, <https://doi.org/10.1007/s10021-007-9018-y>.

Pilegaard, K., Ibrom, A., Courtney, M. S., Hummelshøj, P., and Jensen, N. O.(2011) Increasing net CO_2_ uptake by a Danish beech forest during the period from 1996 to 2009, *Agr. Forest Meteorol.*, **151**, 934–946, <https://doi.org/10.1016/j.agrformet.2011.02.013>.

Posse, G., Lewczuk, N., Richter, K., and Cristiano, P.(2016) Carbon and water vapor balance in a subtropical pine plantation*, iForest –Biogeosci. Forest*., **9**, 736–742, <https://doi.org/10.3832/ifor1815-009>.

Post, H., Hendricks Franssen, H. J., Graf, A., Schmidt, M., and Vereecken, H.(2015) Uncertainty analysis of eddy covariance CO_2_ flux measurements for different EC tower distances using an extended two-tower approach, *Biogeosciences*, 12, 1205–1221, <https://doi.org/10.5194/bg-12-1205-2015>.

Powell, T. L., Bracho, R., Li, J., Dore, S., Hinkle, C. R., and Drake, B. G.(2006) Environmental controls over net ecosystem carbon exchange of scrub oak in central Florida, *Agr. Forest Meteorol.*, **141**, 19–34, <https://doi.org/10.1016/j.agrformet.2006.09.002>.

Prescher, A.-K., Grünwald, T., and Bernhofer, C.(2010) Land use regulates carbon budgets in eastern Germany: From NEE to NBP, *Agr. Forest Meteorol*., **150**, 1016–1025, <https://doi.org/10.1016/j.agrformet.2010.03.008>.

Rambal, S., Joffre, R., Ourcival, J. M., Cavender-Bares, J., and Rocheteau, A.(2004) The growth respiration component in eddy CO_2_ flux from a Quercus ilex mediterranean forest, *Global Change Biol*., **10**, 1460–1469, <https://doi.org/10.1111/j.1365-2486.2004.00819.x>.

Reichstein, M., Rey, A., Freibauer, A., Tenhunen, J., Valentini, R., Banza, J., Casals, P., Cheng, Y., Grünzweig, J. M., Irvine, J., Joffre, R., Law, B. E., Loustau, D., Miglietta, F., Oechel, W., Ourcival, J.-M., Pereira, J. S., Peressotti, A., Ponti, F., Qi, Y., Rambal, S., Rayment, M., Romanya, J., Rossi, F., Tedeschi, V., Tirone, G., Xu, M. and Yakir, D.(2003) Modelling temporal and large-scale spatial variability of soil respiration from soil water availability, temperature and vegetation productivity indices, *Global Biogeochem. Cycles*, **17(4),** doi:10.1029/2003GB002035.

Reverter, B. R., Sánchez-Cañete, E. P., Resco, V., Serrano-Ortiz, P., Oyonarte, C., and Kowalski, A. S.(2010) Analyzing the major drivers of NEE in a Mediterranean alpine shrubland, *Biogeosciences*, **7,** 2601–2611, <https://doi.org/10.5194/bg-7-2601-2010>.

Ruehr, N. K., Martin, J. G., and Law, B. E.(2012) Effects of water availability on carbon and water exchange in a young ponderosa pine forest: Above- and belowground responses, *Agr. Forest Meteorol*., **164**, 136–148, <https://doi.org/10.1016/j.agrformet.2012.05.015>.

Sabbatini, S., Arriga, N., Bertolini, T., Castaldi, S., Chiti, T., Consalvo, C., Njakou Djomo, S., Gioli, B., Matteucci, G., and Papale, D.(2016) Greenhouse gas balance of cropland conversion to bioenergy poplar short-rotation coppice, *Biogeosciences*, **13**, 95–113, <https://doi.org/10.5194/bg-13-95-2016>.

Saleska, S.R., H.R. da Rocha, A.R. Huete, A.D. Nobre, P. Artaxo, and Y.E. Shimabukuro. (2013) LBA-ECO CD-32 Flux Tower Network Data Compilation, Brazilian Amazon: 1999-2006. Data set. Available on-line [http://daac.ornl.gov] from Oak Ridge National Laboratory Distributed Active Archive Center, Oak Ridge, Tennessee, USA, doi:10.3334/ORNLDAAC/1174.

Schroder, I., Kuske, T., and Zegelin, S. (2014) Eddy Covariance Dataset for Arcturus (2011–2013), *Geoscience Australia, Canberra*, Tech. rep., <https://doi.org/102.100.100/14249>.

Scott, R. L., Biederman, J. A., Hamerlynck, E. P., and BarronGafford, G. A.(2015a) The carbon balance pivot point of southwestern U.S. semiarid ecosystems: Insights from the 21st century drought, *J. Geophys. Res.-Biogeosc*i., **120**, 2612–2624, <https://doi.org/10.1002/2015jg003181>.

Scott, R. L., Hamerlynck, E. P., Jenerette, G. D., Moran, M. S., and Barron-Gafford, G. A.(2010) Carbon dioxide exchange in a semidesert grassland through drought-induced vegetation change, *J. Geo phys. Res.*, **115,** G3, <https://doi.org/10.1029/2010jg001348>.

Scott, R. L., Jenerette, G. D., Potts, D. L., and Huxman, T. E.(2009) Effects of seasonal drought on net carbon dioxide exchange from a woody-plant-encroached semiarid grassland, *J. Geophys. Res*.,**114**, G4, <https://doi.org/10.1029/2008jg000900>.

Shao, C., Chen, J., Li, L., Dong, G., Han, J., Abraha, M., and John, R.(2017) Grazing effects on surface energy fluxes in a desert steppe on the Mongolian Plateau:, *Ecol. Appl*., **27**, 485–502, <https://doi.org/10.1002/eap.1459>.

Stoy, P. C., Mauder, M., Foken, T., Marcolla, B., Boegh, E., Ibrom, A., Arain, M. A., Arneth, A., Aurela, M., Bernhofer, C., Cescatti, A., Dellwik, E., Duce, P., Gianelle, D., van Gorsel, E., Kiely, G., Knohl, A., Margolis, H., Mccaughey, H., Merbold, L., Montagnani, L., Papale, D., Reichstein, M., Saunders, M., Serrano-Ortiz, P., Sottocornola, M., Spano, D., Vaccari, F. and Varlagin, A.(2013) A data driven analysis of energy balance closure across FLUXNET research sites: The role of landscape scale heterogeneity, *Agric. For. Meteorol*., **171–172**, 137–152, doi:10.1016/j.agrformet.2012.11.004.

Tagesson, T., Fensholt, R., Guiro, I., Rasmussen, M. O., Huber, S., Mbow, C., Garcia, M., Horion, S., Sandholt, I., HolmRasmussen, B., Göttsche, F. M., Ridler, M.-E., Olén, N., Olsen,J. L., Ehammer, A., Madsen, M., Olesen, F. S., and Ardö, J.(2014) Ecosystem properties of semiarid savanna grassland in West Africa and its relationship with environmental variability, *Global Change Biol*., **21**, 250–264, <https://doi.org/10.1111/gcb.12734>.

Tedeschi, V., Rey, A., Manca, G., Valentini, R., Jarvis, P. G., and Borghetti, M.(2006) Soil respiration in a Mediterranean oak forest at different developmental stages after coppicing, *Global Change Biol*., **12**, 110–121, <https://doi.org/10.1111/j.1365-> 2486.2005.01081.x.

Ulke, A. G., Gattinoni, N. N., and Posse, G.(2015) Analysis and modelling of turbulent fluxes in two different ecosystems in Argentina, *International J. Environ. Pollut*., **58**, 52, <https://doi.org/10.1504/ijep.2015.076583>.

Urbanski, S., Barford, C., Wofsy, S., Kucharik, C., Pyle, E., Budney, J., McKain, K., Fitzjarrald, D., Czikowsky, M., and Munger, J. W.(2007a) Factors controlling CO2 exchange on timescales from hourly to decadal at Harvard Forest, *J. Geophys. Res.*, **112**, <https://doi.org/10.1029/2006jg000293>.

Valentini, R., Angelis, P., Matteucci, G., Monaco, R., Dore, S., and Mucnozza, G. E. S.(1996) Seasonal net carbon dioxide exchange of a beech forest with the atmosphere, *Global Change Biol*., **2**, 199– 207, <https://doi.org/10.1111/j.1365-2486.1996.tb00072.x>.

Wei, S., Yi, C., Hendrey, G., Eaton, T., Rustic, G., Wang, S., Liu, H., Krakauer, N. Y., Wang, W., Desai, A. R., Montagnani, L., Tha Paw U, K., Falk, M., Black, A., Bernhofer, C., Grünwald, T., Laurila, T., Cescatti, A., Moors, E., Bracho, R. and Valentini, R.(2014) Data418 based perfect-deficit approach to understanding climate extremes and forest carbon assimilation capacity, *Environ. Res. Lett*., **9**, 065002, doi:10.1088/1748-9326/9/6/065002.

Wen, X.-F., Wang, H.-M., Wang, J.-L., Yu, G.-R., and Sun, X. M.(2010) Ecosystem carbon exchanges of a subtropical evergreen coniferous plantation subjected to seasonal drought, 2003–2007, *Biogeosciences*, **7**, 357–369, <https://doi.org/10.5194/bg-7-357-2010>.

Wick, B., Veldkamp, E., de Mello, W. Z., Keller, M., and Crill, P.(2005) Nitrous oxide fluxes and nitrogen cycling along a pasture chronosequence in Central Amazonia, Brazil, *Biogeosciences*, **2,** 175–187, <https://doi.org/10.5194/bg-2-175-2005>.

Wohlfahrt, G., Hammerle, A., Haslwanter, A., Bahn, M., Tappeiner, U., and Cernusca, A.(2008) Seasonal and inter-annual variability of the net ecosystem CO_2_ exchange of a temperate mountain grassland: Effects of weather and management, *J. Geophys. Res*., **113**, D8, <https://doi.org/10.1029/2007jd009286>.

Xin, Q., Zhou, X., Wei, N., Yuan, H., Ao, Z., and Dai, Y.(2020) A semiprognostic phenology model for simulating multidecadal dynamics of global vegetation Leaf Area Index. *Journal of Advances in Modeling Earth Systems.*, **12,** e2019MS001935, <https://doi.org/10.1029/2019MS001935>.

Yan, J., Zhang, Y., Yu, G., Zhou, G., Zhang, L., Li, K.,Tan, Z., and Sha, L.(2013) Seasonal and inter-annual variations in net ecosystem exchange of two old-growth forests in southern China, *Agr. Forest Meteorol.,* **182–183**, 257–265, <https://doi.org/10.1016/j.agrformet.2013.03.002>.

Yee, M. S., Pauwels, V. R., Daly, E., Beringer, J., Rüdiger, C.,McCabe, M. F., and Walker, J. P.(2015) A comparison of optical and microwave scintillometers with eddy covariance derived surface heat fluxes, *Agr. Forest Meteorol*., **213**, 226–239, <https://doi.org/10.1016/j.agrformet.2015.07.004>.

Zeller, K. and Nikolov, N.(2000) Quantifying simultaneous fluxes of ozone, carbon dioxide and water vapor above a subalpine forest ecosystem, *Environ. Pollut*., **107**, 1–20, <https://doi.org/10.1016/s0269-7491(99)00156-6>.

Zielis, S., Etzold, S., Zweifel, R., Eugster, W., Haeni, M., and Buchmann, N.(2014) NEP of a Swiss subalpine forest is significantly driven not only by current but also by previous year’s weather, *Biogeosciences*, **11**, 1627–1635, https://doi.org/10.5194/bg-11-1627-2014.
